# Global decarbonization potential of CO_2_ mineralization in concrete materials

**DOI:** 10.1073/pnas.2313475121

**Published:** 2024-07-08

**Authors:** Justin G. Driver, Ellina Bernard, Piera Patrizio, Paul S. Fennell, Karen Scrivener, Rupert J. Myers

**Affiliations:** ^a^Department of Chemical Engineering, Imperial College London, London SW7 2AZ, United Kingdom; ^b^Department of Civil and Environmental Engineering, Imperial College London, London SW7 2AZ, United Kingdom; ^c^Laboratory for Concrete & Construction Chemistry, Empa, Swiss Federal Laboratories for Materials Science and Technology, Dübendorf 8600, Switzerland; ^d^Centre for Environmental Policy, Imperial College London, London SW7 1NE, United Kingdom; ^e^Laboratory of Construction Materials, École Polytechnique Fédérale de Lausanne, Lausanne CH-1015, Switzerland

**Keywords:** life cycle assessment, cement, carbon capture, techno-economic analysis, CO_2_ utilization

## Abstract

CO_2_ mineralization products are often heralded as having outstanding potentials to reduce CO_2_-eq. emissions. However, these claims are generally undermined by incomplete consideration of the life cycle climate change impacts, material properties, supply and demand constraints, and economic viability of CO_2_ mineralization products. We investigate these factors in detail for ten concrete-related CO_2_ mineralization products to quantify their individual and global CO_2_-eq. emissions reduction potentials. Our results show that in 2020, 3.9 Gt of carbonatable solid materials were generated globally, with the dominant material being end-of-life cement paste in concrete and mortar (1.4 Gt y^–1^). All ten of the CO_2_ mineralization technologies investigated here reduce life cycle CO_2_-eq. emissions when used to substitute comparable conventional products. In 2020, the global CO_2_-eq. emissions reduction potential of economically competitive CO_2_ mineralization technologies was 0.39 Gt CO_2_-eq., i.e., 15% of that from cement production. This level of CO_2_-eq. emissions reduction is limited by the supply of end-of-life cement paste. The results also show that it is 2 to 5 times cheaper to reduce CO_2_-eq. emissions by producing cement from carbonated end-of-life cement paste than carbon capture and storage (CCS), demonstrating its superior decarbonization potential. On the other hand, it is currently much more expensive to reduce CO_2_-eq. emissions using some CO_2_ mineralization technologies, like carbonated normal weight aggregate production, than CCS. Technologies and policies that increase recovery of end-of-life cement paste from aged infrastructure are key to unlocking the potential of CO_2_ mineralization in reducing the CO_2_-eq. footprint of concrete materials.

## Significance

Many companies in the field of CO_2_ mineralization and utilization in concrete materials claim that their technologies have astonishingly large potentials to decrease carbon dioxide equivalent (CO_2_-eq.) emissions. However, these claims are frequently poorly evidenced in the scientific literature. Here, we enable objective benchmarking of these claims by presenting a thorough techno-economic-environmental assessment of ten such CO_2_ mineralization and utilization technologies. The results show that the potentials of multiple commercial CO_2_ mineralization and utilization technologies to decrease CO_2_-eq. emissions are overstated and that few are currently more economical than carbon capture and storage for reducing CO_2_-eq. emissions. We discuss the implications of our results on technology development and policymaking in the CO_2_ mineralization and utilization field.

The production of construction materials causes around 13% (~6 Gt CO_2_-eq. in 2015) of global anthropogenic greenhouse gas emissions, most of which come from concrete and steel manufacturing (1). Interest in decarbonizing these materials is growing, including in the application of CO_2_ mineralization to concrete materials (i.e. aggregates, cement, mortar, concrete). CO_2_ mineralization means the process of chemically binding CO_2_ (“carbonation”) in calcium carbonate (CaCO_3_) (Eq. **1**) or other metal carbonates, e.g. MgCO_3_, although CaCO_3_ is currently the most relevant for carbon capture and utilization (CCU) (2), which is what is analyzed here.

[1] $$\text{CaO}+{\text{CO}}_{2}\to \text{Ca}{\text{CO}}_{3}$$

Eq. **1** requires materials containing uncarbonated calcium (shown as CaO here). The most relevant carbonatable materials are solids (“carbonatable solid materials”), since quantities of anthropogenic liquid sources (e.g. domestic and industrial wastewater) are limited and the viability and environmental impacts of using seawater in this context are unclear (3–6). Concrete contains uncarbonated calcium in its “glue-like” cement paste [consisting of solid phases such as portlandite (Ca(OH)_2_), calcium silicate hydrate (C-S-H) gel, etc.]. Cement paste is the matrix that binds aggregate (sand, gravel) particles together in concrete.

A key benefit of CO_2_ mineralization is that calcium carbonate is highly stable at Earth surface conditions, which makes permanent CO_2_ storage possible with high traceability and without the complex technology and infrastructure for permanent underground CO_2_ storage that carbon capture and storage (CCS) requires. CCS refers to a suite of processes that aim to prevent the emission of CO_2_ to the atmosphere, by capturing and then storing CO_2_ from large point-sources in underground reservoirs. These processes include many capture technologies and critical downstream operations for purification, compression, liquefaction, intermediate storage, transport and handling, and eventual storage of CO_2_. The fundamental challenge facing CCS is to reduce its cost: CCS requires additional materials, energy, and infrastructure, which in many cases leads to prohibitive cost increases; because CCS does not produce a saleable product, the cost to abate CO_2_ must be offset by financial incentives (e.g. carbon price). The situation is different for CCU, where CO_2_ is captured and converted into valuable products, the sales of which contribute to offsetting costs (often in addition to financial incentives). Reducing cost is also a key challenge for CCU: The costs for CO_2_ capture and conversion are nonnegligible, meaning that CCU products often struggle to compete economically against conventional products. CO_2_ mineralization is a potentially promising CCUS approach (i.e. a combination of CCS and CCU) since it can result in both saleable products (unlike CCS) and permanent CO_2_ storage (unlike many other CCU products). Construction materials can store CO_2_ for hundreds of years in infrastructure and possibly much longer at end-of-life (e.g. if end-of-life concrete is used as backfill or aggregate, which are typical applications), which is much longer than the achievable CO_2_ storage durations in other CCU products, e.g., fuels (<1 y), chemicals (<10 y), and polymers (<100 y) (7, 8).

Concrete materials are additionally promising for CO_2_ mineralization since they have the largest total market size of any material except water (global production rates in 2020 are as follows: concrete, 17 to 22 Gt y^–1^; mortar, ~8 Gt y^–1^; total aggregates, ~46 Gt y^–1^; see Dataset S1, Tab 38), giving them a uniquely high potential for CO_2_ mineralization and utilization. The excellent potential of concrete for CO_2_ mineralization, i.e., its absorption of CO_2_ throughout its life cycle, is well known (9–11). It varies depending on the atmospheric exposure conditions of the materials in use and during end-of-life, and current estimates indicate that ~20% of the total CO_2_ emitted from cement production could be absorbed through this passive mechanism throughout its life cycle (10). CO_2_ mineralization is also an effective way to valorize solid wastes and industrial by-products since they often contain substantial quantities of uncarbonated calcium and are already used in concrete materials. Such wastes and by-products are generated by various processes, including coal fly ash from coal-fired electricity generation; end-of-life concrete from demolition of concrete structures; and blast furnace slag, bauxite residue, and concrete slurry waste from the production of pig iron, alumina, and concrete, respectively.

Today, many CO_2_ mineralization technologies for concrete materials are emerging, including: carbonated concrete products [e.g., bricks, blocks, pavers; Solidia (12, 13), CarbonBuilt (14)]; carbonated aggregates [e.g., Blue Planet Ltd. (15, 16), Carbon8 Systems (17), OCO Technology (18), Neustark (19)]; Portland cement (PC) clinker (hereafter “clinker”) substitutes [e.g., Fortera (20), HeidelbergCement (21, 22)]; accelerated curing of concrete using CO_2_ [“CO_2_ curing”; CarbonCure (23, 24)]; and biotechnological processes [e.g., Biomason (25)]. Some of these companies claim that their products are “carbon negative” (16) and/or can reduce global CO_2_ emissions from concrete [e.g., “by up to 70%” (12)]. However, few rigorous publicly available life cycle assessment (LCA) and economic analysis studies have interrogated these claims, which is of concern given the significant attention that they receive. LCA of CO_2_ mineralization remains underexplored compared to other decarbonization (i.e., CO_2_-eq. emissions mitigation) technologies like CCS (26).

Previous analyses of the decarbonization potential of CO_2_ mineralization have focused on storage and quantifying maximum theoretical values at the global scale (27–30). Renforth (27) and Pan et al. (30) estimated that CO_2_ mineralization could have reduced CO_2_ emissions by up to 1.5 to 3.4 Gt in 2020 [i.e., comparable to the total CO_2_-eq. emissions footprint of the European Union (27), 3.23 Gt in 2020 (31)] considering supply-side factors only, i.e., CO_2_ uptake capacities of carbonatable materials. This range is large and shows the need for more reliable estimates. These studies poorly address the CO_2_ utilization context because they do not include key demand-side factors such as market sizes of CO_2_ mineralization products, what they can be used for (i.e. their material properties), and the economic performance of their production processes. Consideration of these factors is essential because they significantly limit the commercial feasibility, scale of application, and thus decarbonization potential. Additionally, environmental impacts related to upstream processing of carbonatable materials and life cycle allocation issues (e.g. related to substituting conventional products with CO_2_ mineralization products) have also been poorly considered in previous studies despite their high significance.

This paper aims to assess the global decarbonization potentials of a wide range of CO_2_ mineralization technologies and feedstocks (Table 1), considering the feasibilities of CO_2_ mineralization products to substitute conventional products. Significantly, we report a thorough combined analysis of their i) climate change impacts (quantified using LCA), ii) material properties (e.g. 28 d compressive strength), iii) supply (generation rates of carbonatable materials) and iv) demand (market size) constraints, and v) economic viability (e.g. relative increase in production cost vs. comparable conventional product). We conduct our analysis for year 2020 to provide a reliable snapshot of the current climate change mitigation potential of CO_2_ mineralization in concrete materials. Our analysis covers the three main types of feedstocks for CO_2_ mineralization: 1) carbonatable solid wastes and by-products generated by industrial processes; 2) construction and demolition waste concrete; and 3) concrete products (including concrete blocks and ready-mix concrete, which is concrete that is mixed at plants and transported to site in concrete trucks), which can be directly treated by CO_2_.

**Table 1.** Functions of the CO_2_ mineralization products investigated here and their typical production processes

CO_2_ mineralization product	Function	Typical production process
Inert additives		
Carbonated lightweight aggregate	Lightweight aggregate	Wetting, granulation, carbonation
Carbonated normal aggregate	Normal aggregate	Wetting, shaping, carbonation
Carbonated recycled concrete aggregate	Recycled concrete aggregate	Recycled concrete aggregate production, carbonation
Reactive additives		
Precipitated calcium carbonate (PCC)	Supplementary cementitious material	Calcium extraction, filtration, carbonation/precipitation, filtration/drying
Cement from carbonated end-of-life cement paste (57% clinker)	Cement	Crushing, separation, carbonation, filtration/drying, blending
Composite PC with PCC (75% clinker)	Cement	Calcium extraction, filtration, carbonation/precipitation, filtration/drying, blending
CO_2_ curing		
Carbonate bonded compacts (unreinforced)	Concrete products, unreinforced	Wetting, shaping, carbonation
Carbonatable calcium silicate cement concrete block, unreinforced	Concrete products, unreinforced	Carbonatable calcium silicate cement concrete block production, carbonation
Carbonated PC (CEM I) concrete block, unreinforced	Concrete products, unreinforced	Concrete block production, carbonation
CO_2_-injected ready-mix composite PC concrete (80% clinker)	Ready-mix concrete	Ready-mix concrete production, carbonation

Further details are provided in *SI Appendix*, Supplementary Information S1. PC is Portland cement. CEM I is a standardized type of PC containing typically 95 wt.% clinker and 5 wt.% limestone, excluding calcium sulfate (typical dosage is 0.04 kg per kg cement).

## Results

### Generation Rates of CO_2_-Containing Flue Gases and Carbonatable Solid Materials.

A main constraint in applying CO_2_ mineralization is the supply of suitable feedstocks, including both CO_2_-containing flue gases and carbonatable solid materials (Table 2).

**Table 2.** Global generation rates of carbonatable solid materials

Material	Generation rate in 2020 (Gt/y)
Carbonatable solid materials	
End-of-life concrete	3.27
… of which is cement paste	0.74 ± 0.07
End-of-life mortar	2.73
… of which is cement paste	0.65 ± 0.07
Cement kiln dust	0.2 ± 0.13
Concrete slurry waste	0.22 ± 0.04
Cement bypass dust	0.02 ± 0.01
Blast furnace slag	0.38 ± 0.08
Basic oxygen furnace slag	0.18 ± 0.04
Electric arc furnace slag	0.09 ± 0.02
Coal ashes	0.68 ± 0.48
Air pollution control residues	0.01 ± 0.003
Incineration bottom ash	0.07 ± 0.01
Paper sludge incineration ash	0.02 ± 0.01
Phosphogypsum	0.4 ± 0.04
Glass powders	0.11 ± 0.01
Bauxite residue	0.15 ± 0.01
Incinerated sewage sludge ash	0.02 ± 0.01
Total, excluding non-cement paste fractions in end-of-life concrete and mortar	3.93 ± 1.18
CO_2_	
Total CO_2_ uptake potential of carbonatable solid materials in 2020 (Gt/y)	0.63 ± 0.18
CO_2_ emissions in flue gases from large point-source emitters in 2018 (Gt/y)	21.6

Their physical generation rates in year 2020 were calculated using data from refs. 32–35, for years 2015 to 2020. Their CO2 uptake potentials, and CO2 in flue gases from large point-source emitters in 2018 are shown for comparison. Full calculation details are provided in *SI Appendix*, Supplementary Information S1 and Dataset S1.

Complete conversion of the ~21.6 Gt CO_2_ that was emitted in flue gases from large point-sources in 2018 would require the equivalent of ~28 Gt of CaO and yield up to ~49 Gt of CaCO_3_ (determined using the molecular weights of CO_2_, CaO, and CaCO_3_). This calculation demonstrates that there is effectively an unlimited supply of CO_2_-containing flue gas for CO_2_ mineralization. While the supply of CO_2_-containing flue gases may decrease in the next decades if fossil fuel use is phased out in a consistent manner with 1.5 or 2 °C global temperature rise scenarios (36), it is unlikely to become constraining due to their very high generation rates compared to carbonatable solid materials (Table 2) and the emerging commercial application of CCS, which will increase production of concentrated CO_2_.

To quantitatively show this, we considered a wide range of carbonatable solid materials (Table 2) and estimated their total global generation rate in 2020 to be 3.9 Gt y^–1^ (full details including analysis of future generation rates are provided in *SI Appendix*, Supplementary Information S1). The carbonatable solid material with the highest potential generation rate is end-of-life cement paste (1.39 Gt y^–1^), which is a component of end-of-life concrete (0.74 Gt y^–1^) and mortar (0.65 Gt y^–1^). We expect the supply of this material to increase in the future, consistent with historic global growth in in-use cement stocks (37). Coal fly ash (0.68 Gt y^–1^) and blast furnace slag (0.38 Gt y^–1^) are also generated in significant volumes. We calculate that carbonation of all the materials considered here could have directly absorbed 0.63 Gt CO_2_ in 2020. We consider this to be an upper limit, since it requires: i) 100% recovery of end-of-life concrete and mortar, which is attained in many developed countries [e.g., The Netherlands (38)] but not in many developing countries [e.g., South Africa (39)]; and carbonation of ii) hazardous or iii) already used materials (e.g. blast furnace slag and coal fly ash as clinker substitutes). This result clearly shows that the supply-side constraint on CO_2_ mineralization technologies is the availability of carbonatable solid materials rather than CO_2_-containing flue gases. It also shows that CO_2_ curing technologies are needed to maximize the total uptake of CO_2_ in concrete materials.

### Product-Level Climate Change Impacts of CO_2_ Mineralization.

**Fig. 1.**
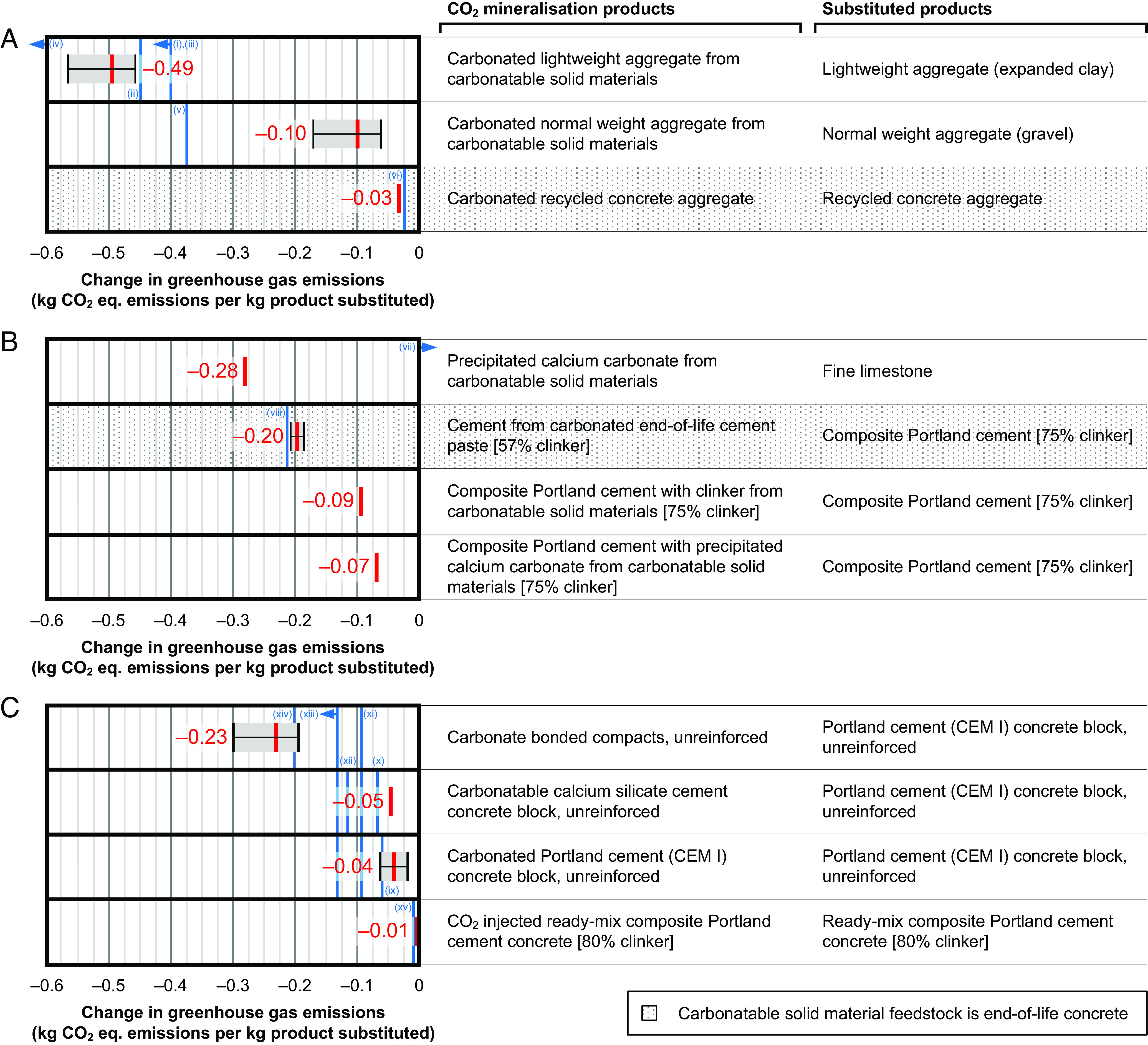
Changes in product-level CO_2_-eq. emissions associated with substitution of conventional products for CO_2_ mineralization products and estimated values for claims made by companies producing CO_2_ mineralization products (blue bars): (*A*) inert additives; (*B*) reactive additives; (*C*) CO_2_ curing. A negative value means that the substitution decreases CO_2_-eq. emissions. A positive value means that the substitution increases CO_2_-eq. emissions. The red values correspond to the thick red bars and represent typical values for the corresponding substitutions. Lower and upper changes in CO_2_-eq. emissions, determined through sensitivity analysis (*SI Appendix*, Supplementary Information S1 and Dataset S1) are shown as thin black bars, with the ranges between these values shaded gray. Commercial CO_2_ mineralization products corresponding to the blue bars are labeled *i* to *xv* and are (values are in kg CO_2_-eq. emissions per kg product substituted): (i) Carbon8 Systems, <–0.4; (ii) OCO Technologies, –0.45; (iii) Low Carbon Materials, <–0.4; (iv) Blue Planet, –0.77; (v) Blue Planet, –0.37; (vi) Neustark, –0.02; (vii) Fortera (supplementary cementitious material), +0.23; (viii) HeidelbergMaterials, –0.21; (ix) Carbonaide, –0.06; (x) Solidia, –0.07; (xi) CarbonBuilt, –0.09; (xii) Fortera (cement), –0.12; (xiii) Carbicrete, <–0.13; (xiv) Carbstone, –0.20; (xv) CarbonCure, –0.01. Full calculation details are shown in Dataset S1. CEM I is a standardized type of PC containing typically 95 wt.% clinker and 5 wt.% limestone, excluding calcium sulfate (typical dosage is 0.04 kg per kg cement).

Our results show that all the CO_2_ mineralization products considered here reduce CO_2_-eq. emissions by between 0.01 and ~0.49 CO_2_-eq. per kg product substituted when used to substitute conventional products of comparable functionality (Fig. 1). This wide range shows that the CO_2_ mineralization-conventional product substitution pairing greatly affects the environmental benefit associated with using the CO_2_ mineralization product.

Of the CO_2_ mineralization products considered here, the greatest CO_2_-eq. emissions reduction can be achieved through the production of carbonated lightweight aggregate since i) it is carbon negative (−0.06 to −0.17 kg CO_2_-eq. emissions per kg product), ii) it avoids relatively high amounts of CO_2_-eq. emissions when used to substitute conventional lightweight aggregate (expanded clay; +0.4 kg CO_2_-eq. per kg product), and iii) because a high fraction of its (carbonated) mass is CaCO_3_, it has relatively high-CO_2_ uptake. Similarly, using carbonatable solid materials to produce precipitated calcium carbonate (PCC) (–0.27 kg CO_2_-eq. emissions per kg product) and its application to substitute fine limestone (+0.01 kg CO_2_-eq. emissions per kg product) also has a high potential to reduce CO_2_-eq. emissions at the product-level. Conversely, the relatively low masses of CaCO_3_ in most CO_2_ curing type CO_2_ mineralization products (Fig. 1*C*) means that they typically have relatively small CO_2_-eq. emissions reduction potentials at the product scale. However, the effectively unlimited supply of emitted CO_2_ and reserves of primary raw materials for concrete manufacturing (e.g. limestone) indicate the larger theoretical decarbonization potentials of CO_2_ curing technologies at the market scale. Therefore, this analysis highlights the importance of comparing products with comparable functionality (i.e. within individual product classes) and considering both supply and demand constraints.

The CO_2_-eq. emissions reduction potentials of substituting conventional PC concrete products by either carbonated PC concrete products or carbonatable calcium silicate cement concrete products [e.g., Solidia (12) are similar (Fig. 1*C*)]. There are three main reasons for this, which somewhat cancel each other out: i) carbonation reabsorbs process-derived CO_2_-eq. emissions from limestone calcination, reducing process emissions; ii) carbonatable calcium silicate cement is produced at high temperatures [~1,200 °C (40); which is a modest reduction compared to clinker kiln temperatures of ~1,450 °C], limiting its reduction in fossil fuel–derived emissions; and iii) only a small fraction of the mass of concrete products is cement (typically 15%), diluting the difference in CO_2_-eq. emissions reductions for these CO_2_ mineralization technologies. This analysis is most relevant for dense “precast” concrete products, since porous concrete products, e.g., cinder blocks, will much more rapidly carbonate during use if they are exposed to the atmosphere, reducing the climate change benefit of CO_2_ mineralization in those products.

There is a greater CO_2_-eq. emissions reduction potential associated with using end-of-life concrete to produce cement from carbonated end-of-life cement paste (57% clinker, 38% carbonated end-of-life cement paste, 5% calcium sulfate; −0.20 kg CO_2_-eq. emissions per kg product) than composite PC with clinker from carbonatable solid materials (75% clinker, 15% ground granulated blast furnace slag, 5% fine limestone, 5% calcium sulfate; –0.09 kg CO_2_-eq. emissions per kg product). Cement from carbonated end-of-life cement paste also has a greater potential to reduce CO_2_-eq. emissions than composite PC with PCC from carbonatable solid materials (i.e., 75% clinker, 20% PCC from carbonatable solid materials, 5% calcium sulfate; –0.07 kg CO_2_-eq. emissions per kg product).

The lower CO_2_-eq. footprint for cement from carbonated end-of-life cement paste than composite PC with clinker from carbonatable solid materials, conventional composite PC (75% clinker), and composite PC with PCC from carbonatable solid materials (75% clinker) emphasizes the importance of the former material in developed countries with established building stocks and hence substantial generation rates of end-of-life concrete. The partial use of carbonatable solid materials to replace cement kiln feedstock in the production of composite PC with clinker from carbonatable solid materials reduces process emissions but not fossil fuel–derived emissions, which explains its moderate reduction in CO_2_-eq. emissions relative to conventional composite PC (75% clinker). The maximum amount of carbonatable solid materials that can be added into the cement kiln without compromising product quality (i.e., to achieve the target clinker composition) is typically 25 to 30% of the total feedstock mass (we use a value of 26% here, see Dataset S1, Tab 13). The moderate reduction in CO_2_-eq. emissions of composite PC with PCC from carbonatable solid materials (75% clinker) relative to conventional composite PC (75% clinker) is explained by the near-zero CO_2_-eq. emissions footprint of fine limestone production (0.008 kg CO_2_-eq. emissions per kg product), which is the material that PCC substitutes in this CO_2_ mineralization product.

Our results show the variable validity of claims made by companies regarding the climate change impacts of their CO_2_ mineralization products (Fig. 1, blue lines). Claims made by Carbon8 Systems (Fig. 1(i)), OCO Technologies (Fig. 1(ii)), Low Carbon Materials (Fig. 1(iii)), Neustark (Fig. 1(vi)), HeidelbergMaterials (Fig. 1(viii)), Carbonaide (Fig. 1(ix)), Solidia (Fig. 1(x)), Carbstone (Fig. 1(xiv)), and CarbonCure (Fig. 1(xv)) are generally consistent with our results (Fig. 1, blue lines are located near red lines) and thus they support their validity. These companies are more likely to have published peer-reviewed technical papers (41, 42), LCA studies (23, 43) or third-party commissioned LCA reports (44). Our results are inconsistent with claims made by Blue Planet (Fig. 1(iv and v)) and Fortera (Fig. 1(vii)) (Fig. 1, blue lines are not located near red lines), and so demonstrate the need for more independently verified LCA studies and complete reporting of those CO_2_ mineralization technologies. Such reporting is also needed to explain the intermediate locations of CarbonBuilt (Fig. 1(xi)) and Carbicrete (Fig. 1(xiii)) technologies between carbonate bonded compacts (unreinforced) and carbonated PC (CEM I) concrete blocks (unreinforced). The result for Fortera (Fig. 1(xii)) indicates its greater decarbonization potential than Solidia (Fig. 1(x). This analysis underscores the dominant influence that the baseline of comparison used in a claim has on its validity and thus the importance of verifiable and clear information about company products being available.

### Economic Analysis of CO_2_ Mineralization.

**Fig. 2.**
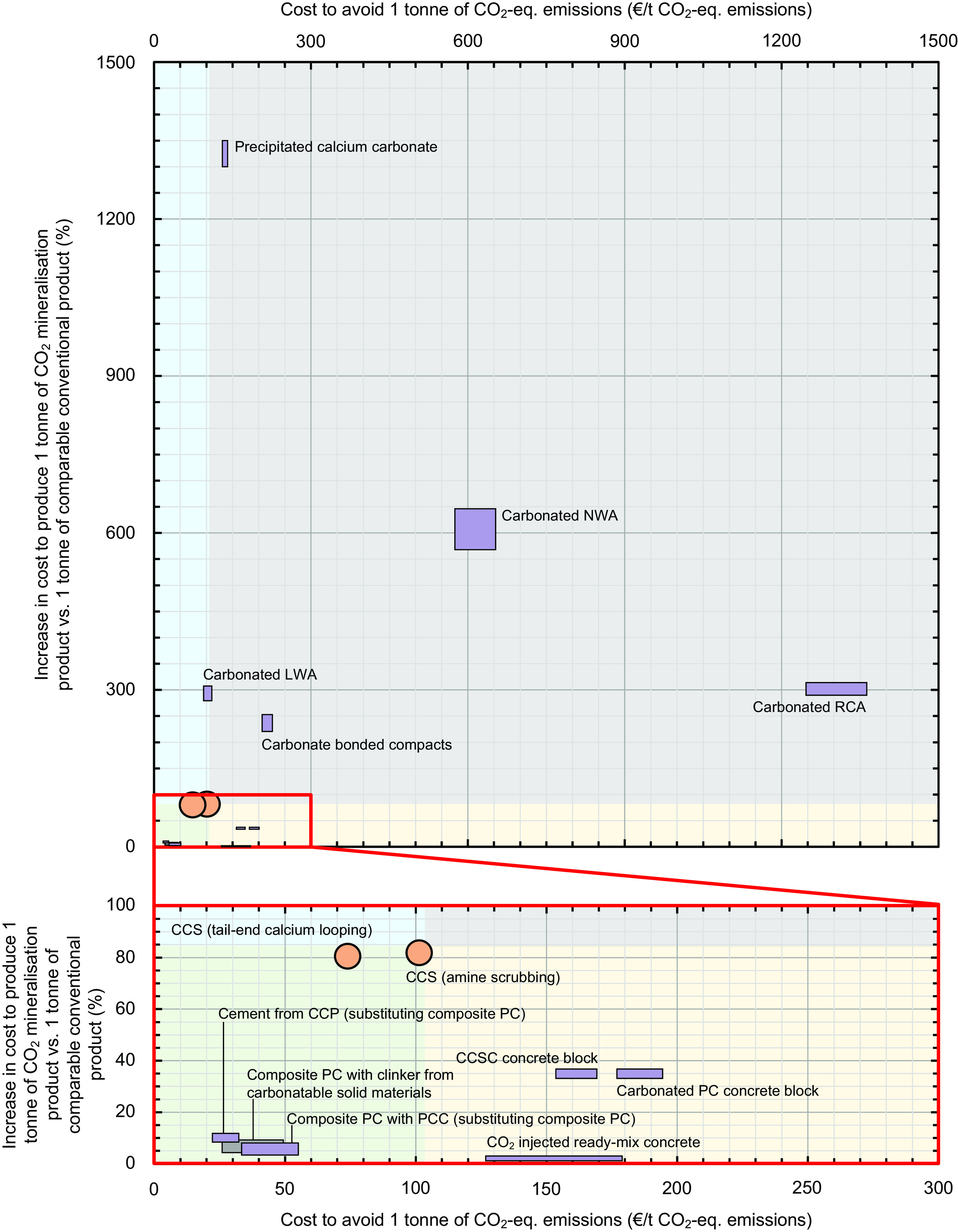
Comparative economic analysis of CO_2_ mineralization products vs. conventional products in terms of production and CO_2_-eq. emissions mitigation costs. PC: Portland cement. PCC is precipitated calcium carbonate produced from carbonatable solid materials. CCSC: carbonatable calcium silicate cement. LWA, NWA, RCA: lightweight, normal weight, and recycled concrete aggregates, respectively. Orange shaded circles refer to the abatement of CO_2_-eq. emissions from PC production via use of CCS in the cement plant vs. conventional cement production. Purple shaded rectangles refer to results for CO_2_ mineralization technologies. The gray shaded rectangle refers to results for composite PC with clinker from carbonatable solid materials, which is not a CO_2_ mineralization technology but competes for the same carbonatable solid materials so is included here. Rectangle size corresponds to the uncertainty in the results. The (i) green, (ii) yellow, (iii) blue, and (iv) gray shaded regions indicate envelopes of current economic performance for products that are i) relatively cheaper per unit of production and to avoid CO_2_-eq. emissions than CCS, “superior decarbonization technologies”; ii) cost-competitive per unit of production but more expensive to avoid CO_2_-eq. emissions than CCS, “technically viable technologies”; iii) more expensive per unit of production but cheaper to avoid CO_2_-eq. emissions than CCS, “viable decarbonization technologies”; and iv) more expensive per unit of production and to avoid CO_2_-eq. emissions than CCS, “uncompetitive technologies”. For full calculation details, see *SI Appendix*, Supplementary Information S1 and Dataset S1, including a sensitivity analysis on the production of cement from carbonated end-of-life cement paste since it is currently highly competitive both economically and environmentally.

Of all the CO_2_ mineralization technologies analyzed here, costs to avoid 1 t of CO_2_-eq. emissions are only lower than CCS (€80 to 100/t CO_2_-eq.) for cement from carbonated end-of-life cement paste (€22 to 33/t CO_2_-eq. emissions avoided) (Fig. 2). This technology costs slightly less to avoid CO_2_-eq. emissions than composite PC with clinker from carbonatable solid materials (€25 to 49/t CO_2_-eq. emissions avoided), which is also less expensive than CCS for reducing CO_2_-eq. emissions. Since CO_2_ prices in the European emissions trade scheme during 2022 ranged from €60 to 100/t CO_2_ (*SI Appendix*, Supplementary Information S1), our results underline the excellent current cost-competitiveness of these two technologies in Europe. Relative to the landscape of CO_2_ mineralization technologies, Fig. 2 shows that CCS [notably calcium looping (45)] is a promising method to reduce CO_2_-eq. emissions from cement production.

In Fig. 2, the most promising technology substitutions are located toward the bottom left [smallest increase in cost to produce 1 ton of CO_2_ mineralization product vs. 1 ton of comparable conventional product (%), and lowest cost to avoid 1 ton CO_2_-eq. emissions (€/t CO_2_-eq. emissions)]. Technology substitutions that cost more to produce and to avoid CO_2_-eq. emissions than their conventional counterparts are located toward the *Top Right* corner of Fig. 2. There is large variation in the locations of CO_2_ mineralization technologies in Fig. 2, and thus their environmental and economic performance.

This result demonstrates that research, business, and policy activities should target the individual CO_2_ mineralization technologies with high environmental and/or economic performance (Fig. 2, *Bottom Left*) rather than the general field, since some technologies are currently significantly uncompetitive (carbonated recycled concrete aggregate, carbonated normal weight aggregate, carbonate bonded compacts), and so expending effort on them may be counterproductive to addressing the urgent climate crisis.

In terms of production cost, i) CO_2_-injected ready-mix concrete (+2%), ii) composite PC with PCC (+4 to +7%), iii) composite PC with clinker from carbonatable solid materials (+5 to +9%), and iv) cement from carbonated end-of-life cement paste (+9 to +12%) are cost-competitive with their comparable substitute technologies. An additional benefit of composite PC with clinker from carbonatable solid materials relative to cement from carbonated end-of-life cement paste is that its capital cost requirement is lower, reducing implementation barriers including risk. PCC, carbonate bonded compacts, and carbonated recycled concrete, lightweight, and normal weight aggregates currently cost substantially more to produce than their comparable conventional products (Fig. 2, blue and gray shaded regions), which provides a significant barrier to their wider commercial adoption.

Therefore, cement from carbonated end-of-life cement paste, composite PC with clinker from carbonatable solid materials, and composite PC with PCC are the technologies of those analyzed here that are currently cost-competitive in terms of both production cost and cost to avoid CO_2_-eq. emissions and are the most promising. The increased costs of most of the other CO_2_ mineralization technologies to avoid CO_2_-eq. emissions compared to CCS in the present-day context indicates that they must offer significant technical benefit(s) relative to their conventional counterpart technologies to be cost-competitive and should not currently be classified as predominantly CO_2_-eq. emissions reduction technologies.

### Market-Level Decarbonization Potential of CO_2_ Mineralization.

**Fig. 3.**
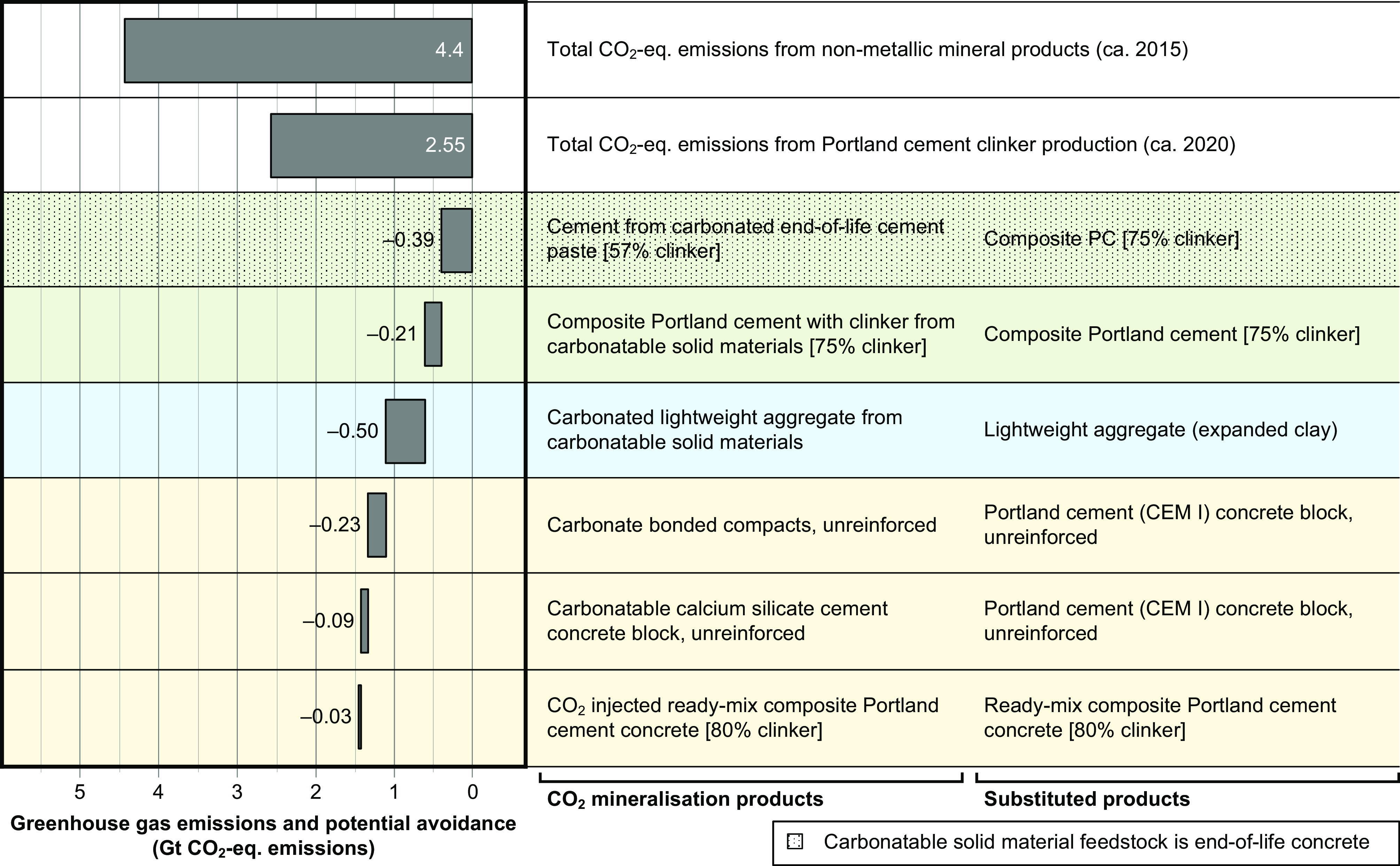
Potential market-level CO_2_-eq. emissions reductions associated with substitution of conventional products for CO_2_ mineralization products in a scenario that reduces the most associated CO_2_-eq. emissions at full-scale implementation. The time required to achieve full-scale implementation varies depending on the technology but is in the order of years. The scenario cascades the use of carbonatable solid materials, prioritizing technologies that have higher CO_2_-eq. emissions reduction efficiency per mass of carbonatable solid material (*SI Appendix*, Supplementary Information S1 and Fig. S13) and are more economically competitive (Fig. 2). PC is Portland cement. CEM I is a standardized type of PC containing typically 95 wt.% clinker and 5 wt.% limestone, excluding calcium sulfate (typical dosage is 0.04 kg per kg cement). Composite PC with clinker from carbonatable solid materials is included since it competes for the supply of carbonatable solid materials but is not a CO_2_ mineralization product. Current techno-economic-environmental performance is indicated using the same color scheme in Figs. 2 and 3: Cost-competitive technologies are shaded green; technically viable technologies are shaded yellow; viable decarbonization technologies are shaded blue; and uncompetitive technologies are shaded gray. For full calculation details, see *SI Appendix*, Supplementary Information S1, Section S6 and Dataset S1, Tab 28. The total CO_2_-eq. emissions from nonmetallic mineral products is from ref. 1.

We modeled a scenario that efficiently uses carbonatable solid materials to maximize the total reduction in CO_2_-eq. emissions at the market-level (Fig. 3), prioritizing technologies that increase production cost by less than CCS or cost less to avoid CO_2_-eq. emissions than by CCS (green, yellow, and blue shaded regions, Fig. 2). Our results (Fig. 3) show that the cumulative decarbonization potential of currently cost-competitive CO_2_ mineralization was 0.39 Gt CO_2_-eq. in 2020. It is achieved by producing 2 Gt of cement from carbonated end-of-life cement paste, which would have totally consumed the potential global supply of end-of-life cement paste in concrete and mortar. This decarbonization potential is equivalent to avoiding 15% of CO_2_-eq. emissions from cement production, or ~9% of CO_2_-eq. emissions from the production of nonmetallic minerals (i.e. cement, lime, plaster, glass, bricks) (46).

The cumulative decarbonization potential of currently cost-competitive CO_2_ mineralization related technologies increases to 0.60 Gt CO_2_-eq. in 2020 by including sufficient production of composite PC with clinker from carbonatable solid materials to meet the remaining global demand for cement (2.2 Gt). Its theoretical maximum value was 1.46 Gt CO_2_-eq. in 2020, which is achieved by additionally including carbonated lightweight aggregate from carbonatable solid materials, carbonate bonded compacts (unreinforced), carbonatable calcium silicate cement concrete products (unreinforced), and CO_2_-injected ready-mix concrete. This theoretical maximum is an upper value since it includes CO_2_ uptake by carbonatable calcium silicate cements in both dense and porous concrete products; porous concrete typically naturally carbonates within a few years, which limits the climate change benefit of applying CO_2_ mineralization to these products. Our calculated theoretical maximum decarbonization potential of CO_2_ mineralization (1.46 Gt CO_2_-eq. in 2020) is significantly lower than Blue Planet’s claim (47) that “*half of all anthropogenic CO_2_ can be captured & permanently stored using Blue Planet Systems’ geomimetic mineralization technology*” (17.5 Gt CO_2_-eq. emissions in 2020) since it is severely constrained by the limited total generation rate of carbonatable solid materials (3.93 Gt in 2020, Table 2).

We calculate a significantly higher market-level CO_2_-eq. emissions reduction potential for cement from carbonated end-of-life cement paste (0.39 Gt CO_2_-eq.) than composite PC with clinker from carbonatable solid materials (0.21 Gt CO_2_-eq.). This is because, in our scenario, the latter technology is constrained by the remaining market size for cement (2.2 Gt, down from 4.2 Gt) after exhaustion of the supply of end-of-life concrete and mortar that is used as feedstock for the former technology.

## Discussion

We quantified the maximum global potential of CO_2_ mineralization in concrete materials to reduce CO_2_-eq. emissions using economically competitive technologies to be 0.39 Gt CO_2_-eq. in 2020. This is an upper limit, e.g., considering complete recovery of end-of-life concrete and mortar. It is substantially lower than values from similar analyses [e.g., 4.02 Gt CO_2_-eq. per year (30), which confirms that supply- (carbonatable solid material generation rates) and demand-side (market segment sizes) limitations substantially affect the decarbonization potential of CO_2_ mineralization. Additionally, applying a LCA approach is important since the upstream source of carbonatable solid materials (e.g. primary limestone, industrial by-products) greatly influences both the supply of these feedstocks and the modeled climate change impacts of CO_2_ mineralization products.

Our results show that currently cost-competitive CO_2_ mineralization technology could have mitigated up to 15% of the “hard-to-abate” greenhouse gas emissions from cement production, or 9% of those from nonmetallic minerals production (i.e., cement, lime, plaster, other nonmetallic minerals), or 0.8% of global anthropogenic CO_2_-eq. emissions in 2020. A reason for this significant decarbonization potential is that CO_2_ mineralization products can be both carbon negative (i.e. result in a net decrease in atmospheric CO_2_ through their production) and substitute relatively high-CO_2_ products, e.g. clinker. Achieving this decarbonization potential only requires use of cement from carbonated end-of-life cement paste, which demonstrates its high feasibility since the technology is currently cost-competitive with conventional composite PC.

Several CO_2_ mineralization technologies are currently not economically competitive in terms of reducing CO_2_-eq. emissions relative to CCS. They are PCC (from carbonatable solid materials), carbonate bonded compacts, CO_2_-injected ready-mix concrete, carbonatable calcium silicate cement concrete products, carbonated unreinforced PC concrete products, carbonated normal weight aggregate, and carbonated recycled concrete aggregate. Although production of carbonated normal weight and recycled aggregate are carbon negative (Fig. 1), they are currently very economically uncompetitive and thus unpreferred relative to both conventional natural and recycled aggregate production, and CCS (Fig. 2).

Our market-level LCA results show that 1.8 Gt of carbonatable solid materials would have remained unutilized after production of both cement from carbonated end-of-life cement paste and composite PC with clinker from carbonatable solid materials if those technologies were prioritized for CO_2_ mineralization. CO_2_ mineralization can enable utilization of these carbonatable solid materials, reducing environmental impacts and risks associated with their disposal. Our results (Fig. 2) show that the CO_2_ mineralization technology with the most potential in this regard is carbonated lightweight aggregate production, but that there is currently a need to reduce its production cost by 50 to 67% to be competitive with conventional expanded clay aggregate production and be cheaper than CCS at the market scale. This would unlock the potential to reduce CO_2_-eq. emissions by 0.5 Gt (Fig. 3).

However, environmental issues other than climate change, e.g., human toxicity, ecotoxicity, naturally occurring radioactivity, must be considered in evaluating the utilization potential of many carbonatable solid materials, because they can be hazardous. An example here is carbonated aggregates produced from air pollution control residues, which can contain toxic dioxins and furans (48). These substances require treatment to be destroyed, e.g., at high temperatures (>871 °C) with good fuel/air mixing (49), so achieving these conditions during their conversion into carbonated aggregate is important to avoid their leaching into the environment and subsequent human exposure during use. Hence monitoring of hazardous substance contents in carbonatable solid materials and CO_2_ mineralization products, and regulation that prevents their unsafe use is needed. Policymakers should employ the precautionary principle (50) in regulating the (inherently dissipative) use of potentially hazardous carbonatable solid materials in concrete, since concrete is used extensively worldwide in buildings and infrastructure where human and environmental exposure risks are high.

Three CO_2_ curing technologies are currently marginally cost-competitive: carbonatable calcium silicate cement concrete blocks, carbonated PC concrete blocks, and CO_2_-injected ready-mix concrete. Improving their economic viability to become cheaper than CCS currently requires 25 to 60% reductions in production costs. Achieving this cost reduction has the potential to reduce CO_2_-eq. emissions by 0.12 Gt (Fig. 3), since practically infinite amounts of CO_2_-rich flues are available globally. Our economic analysis also shows that CO_2_ mineralization is not currently viable for producing low-cost products such as aggregate due to their much lower market prices than the typical CO_2_ mineralization process cost [costs to produce fine limestone, gravel, recycled concrete aggregate, and lightweight aggregate are €3 to 18/t product vs. ~€40/t product for CO_2_ mineralization of cement kiln dust (51)]. For these low-cost products, it is currently either significantly cheaper to reduce CO_2_-eq. emissions through CCS (Fig. 2, *x*-axis) or to produce the conventional products (Fig. 2, *y*-axis).

Policy can reduce production costs for CO_2_ mineralization technologies through a variety of direct (i.e., economic subsidies) and indirect (e.g., investing in research for process improvements) mechanisms. Our study provides evidence for CCUS policymaking including identifying CO_2_ mineralization technologies that are currently more economically feasible and increasing understanding of their production costs, relevant market sizes, and CO_2_-eq. emissions reduction potentials. Such information is important since poor understanding of these factors is cited as a key reason why CO_2_ utilization projects in the UK have historically received poorer policy support and access to public funds than CCS (52, 53). We note that CCUS policy support varies regionally, e.g., recent developments such as IRA 45Q in the US and the “Fit-for-55” package in the EU outline provisions specifically for CO_2_ utilization technologies (54, 55). We thus recommend country/region-specific studies of currently marginally competitive CO_2_ mineralization technologies (Fig. 3, blue and yellow regions) to establish their local economic performance and guide policy development in this context.

Globally, the bottlenecks to the application of CO_2_ mineralization are the limited supply of carbonatable solid materials and market demand for some product categories (e.g. lightweight aggregate). Importantly, the potential supply of the main carbonatable solid material, end-of-life cement paste, is expected to increase in the coming decades due to aging of growing building and infrastructure stocks, thereby increasing the future significance of CO_2_ mineralization in producing concrete materials. A key priority is thus to implement policies that encourage increased recovery of construction and demolition waste, end-of-life concrete, and end-of-life cement paste, especially in developing countries where recovery rates are lower (39). Clearly, the promotion of a circular economy involving recycling of end-of-life concrete should be an international priority to realize the potential of CO_2_ mineralization and decarbonize the production of concrete materials.

## Materials and Methods

The methodology and data used in this paper are described and presented comprehensively in the *SI Appendix*, Supplementary Information S1 and Dataset S1. We briefly present a summary of key methodological aspects here.

### Estimating CO_2_ Uptake Potentials of Carbonatable Solid Materials.

Values of CO_2_ uptake potentials for carbonatable solid materials were estimated from many reported chemical compositions, at least several for each material, considering their content of calcium (CaO) and loss on ignition (LOI, i.e., the mass lost by a material after firing to an elevated temperature, typically ~1,000 °C, due to the release of volatile components such as H_2_O and CO_2_, and oxidation). Our estimation is based on the assumption that half of the LOI is due to CO_2_ from CaCO_3_ (i.e. that which existed prior to a dedicated carbonation process), and the other half from other components (e.g. H_2_O). It also assumes that all of the CaO content in the material can be carbonated. Calculations showing the validity of this estimation are shown in *SI Appendix*, Supplementary Information S1.

We selected a wide range of carbonatable solid materials that have significant potentials to result in economically competitive CO_2_ mineralization and utilization. This criterion of positive economic competitiveness excludes many alkaline anthropogenic resources such as most mining residues because their applications (notably lightweight and normal weight aggregate production) are limited in the utilization context: The potential of CO_2_ mineralization in lightweight aggregate production is limited by market size (Dataset S1, Tab 38); and production of normal weight aggregate through CO_2_ mineralization is currently very uneconomic (Fig. 2). Additionally, the increased transportation requirements for utilization of mining residues relative to other carbonatable solid materials like end-of-life concrete (longer and/or more complex journeys to CO_2_ mineralization product manufacturing sites) can substantially reduce their economic viability further. Poorly economically competitive alkaline anthropogenic resources are rather relevant for CO_2_ storage (also known as accelerated weathering).

We also selected carbonatable solid materials based on those with the highest global generation rates, since our study aims to assess the global decarbonization potentials of CO_2_ mineralization and utilization. We note that since there is currently substantial industrial interest in using end-of-life cement paste (56, 57), and it is currently produced in much lower quantities than cement, we expect it to be mostly consumed in the region where it is generated rather than being transported internationally.

We estimated the generation rates of carbonatable solid materials for year 2020 since it was the most recent year that had all the data we needed for our analysis at the time that we conducted it, and since material production volumes (e.g. cement) are similar to current values. We also estimate and discuss future generation rates of various carbonatable solid materials (*SI Appendix*, Supplementary Information S1, Section S2.2).

### Product-Level Climate Change Impacts of CO_2_ Mineralization Technologies.

Product-level climate change impacts of CO_2_ mineralization products were assessed by LCA using OpenLCA (v.1.11.0). The functional unit was defined as 1 kg of material for both conventional and CO_2_ mineralization products, with the corresponding 100-y global warming potential calculated for each in units of kg CO_2_-eq./kg of material by application of the IPCC 2013 impact assessment methodology. Datasets from ecoinvent (v.3.8) and the literature were used; however, we also developed our own datasets for some products by using reported descriptions of their production processes and mass balances. The unit process data used in our LCA study are described in detail in *SI Appendix*, Supplementary Information S1 and shown in Dataset S1.

The literature datasets used describe pilot and industrial scale applications of CO_2_ mineralization technologies: PCC (58); cement from carbonated end-of-life cement paste (42, 59); carbonated lightweight aggregate (41); carbonated normal weight aggregate and carbonate bonded compacts (unreinforced) (60); CO_2_-injected ready-mix PC concrete [80% clinker] (23); and carbonatable calcium silicate cement concrete blocks (unreinforced) (61).

For the datasets developed here, we assumed that CO_2_ mineralization products, except carbonated lightweight aggregate, carbonated normal weight aggregate, carbonate bonded compacts (unreinforced), and carbonatable calcium silicate cement concrete blocks, are produced at a cement plant with a typical flue of concentrated CO_2_ (32 vol.%), meaning negligible costs and climate change impacts for transportation of CO_2_. A CO_2_ capture rate of 50% was used for producing carbonated aggregates and end-of-life cement paste (62). A CO_2_ capture rate of 85% was used for PCC production, which is typical for this process (63). We assumed a water/solids ratio of 0.13 w/w for processes employing wet carbonation, which is an averaged value within the range usually reported for rotary-type reactors (62, 64–66). For carbonated lightweight and normal weight aggregate production, carbonate bonded compacts (unreinforced), and production of carbonatable calcium silicate cement concrete blocks, we included environmental impacts associated with capturing, separating, and compressing CO_2_-rich flue gas into liquid CO_2_, since pure CO_2_ is generally needed for carbonation of carbonatable solid materials other than end-of-life cement paste (and end-of-life concrete and mortar is preferentially used for producing cement from carbonated end-of-life cement paste).

All the CO_2_ in flue gas that is absorbed by CO_2_ mineralization products was considered as avoided CO_2_ emissions, and the use of carbonatable solid materials was treated as avoided landfilling, i.e., both as environmental credits. CO_2_ in feedstock flue gas was treated as waste. Carbonatable solid materials were assumed to be burden free at their points of generation and have a fixed transportation distance by lorry of 300 km.

To evaluate the validity of claims made by companies about the climate change impacts of their CO_2_ mineralization products, we translated them into quantities that are directly comparable to our results (Fig. 1). We did this using the unit process datasets developed here for exemplar CO_2_ mineralization technologies in the major commercial product classes. Complete details of the calculations used are shown in Dataset S1. The comparisons should be interpreted as indicative rather than definitive due to the uncertainties associated with the commercial technologies and company claims. There are two main sources of uncertainty here: 1) (lack of) availability of transparent and reliable descriptions of commercial CO_2_ mineralization technologies; and 2) often poor reporting of methodologies used by companies to arrive at their claims (e.g., unclear allocation rules, claims are often relative to baseline products or systems that are poorly defined). To ensure comparable functionality of CO_2_ mineralization and conventional products, we treat Fortera ReAct^™^ Max Strength and Fortera ReAct™ Max Flow as being comparable with fine limestone for partial cement replacement (i.e., a supplementary cementitious material), and Fortera ReAct™ Pure as a cement that is applicable in concrete products but not ready-mix concrete due to its significantly different chemistry and properties than conventional PCs.

### Economic Analysis of CO_2_ Mineralization Technologies.

We first calculated the costs to produce CO_2_ mineralization and comparable conventional products (€/t product). We then derived corresponding costs to avoid CO_2_-eq. emissions (€/t CO_2_-eq. avoided) by combining these results with those from the product-level LCA study (kg CO_2_-eq. emissions avoided per kg product substituted). Costs and financial assumptions associated with each product were harmonized using the Euro average monetary value for year 2021.

Our techno-economic analysis study quantifies costs for Portland clinker production in a reference cement kiln, Portland clinker production in a reference cement kiln retrofitted with CCS, and a typical carbonation process for CO_2_ mineralization. The cost to produce Portland clinker in a reference cement kiln (€60.8/t, without CCS) was adopted to derive costs to produce related conventional products, based on typical mix designs (*SI Appendix*, Table S9 and Supplementary Information S1) and raw material costs (*SI Appendix*, Table S12 and Supplementary Information S1). Similarly, the cost of the carbonation process (€41.7/t) was adopted to calculate the costs to produce CO_2_ mineralization products except CEM I concrete blocks (unreinforced), for which the cost of CO_2_ curing was derived from the literature; and CO_2_-injected ready-mix composite PC concrete, for which we only account for the additional cost associated with CO_2_ separation. For Portland clinker production with CCS, we considered retrofitting the reference cement kiln with two archetypal technologies: amine scrubbing and tail-end calcium looping. In these cases, the costs of cement production (with CCS) were derived from the results of a detailed techno-economic analysis study (67).

Monte Carlo simulations were conducted to quantify the effects of key uncertainties on the costs to produce both CO_2_ mineralization and comparable conventional products, i.e., fuel and material inputs, as well as capital costs of carbonation and CCS processes. The results were used to derive the uncertainty (expressed as percentage errors) associated with the economic analysis results (Fig. 2 and *SI Appendix*, Table S13 and Supplementary Information S1).

### Market-Level Climate Change Impacts of CO_2_ Mineralization Technologies.

Potential changes in climate change impacts (Δ*I_j_*) were calculated using LCA results at the product-level and upscaled to the market-level using Eq. **2**.

[2] $$\Delta I_{j}=M_{j}\left(i_{j,{\mathrm{CO}}_{2}\;mineralization}-i_{j,conventional}\right)$$

Where greenhouse gas emissions are denoted by *i_j_* (kg CO_2_-eq. emissions) for a given product in market segment *j*, with subscripts “CO_2_ mineralization” and “conventional” referring to the respective product classes. The size of market segment *j* is represented by *M_j_* (kg/y).

We combined our product-level LCA (Fig. 1) and economic analysis (Fig. 2) results with global generation rates of carbonatable solid materials and market segment sizes for CO_2_ mineralization products to determine the (global) market-level decarbonization potential of CO_2_ mineralization in concrete materials (Fig. 3). These technologies are i) cement from carbonated end-of-life cement paste; ii) composite PC with clinker from carbonatable solid materials; iii) carbonated lightweight aggregate; iv) carbonate bonded compacts from carbonatable solid materials (unreinforced); v) carbonatable calcium silicate cement concrete; and vi) CO_2_-injected ready-mix concrete. This scenario prioritizes utilization of end-of-life concrete and mortar for the production of cement from carbonated end-of-life cement paste, since i) it typically has a higher CO_2_-eq. emissions reduction per mass of carbonatable solid materials used (0.23 to 0.63 kg CO_2_-eq. emissions avoided per kg carbonatable solid materials used) than composite PC with clinker from carbonatable solid materials (0.28 kg CO_2_-eq. emissions avoided per kg carbonatable solid materials used), and ii) the market size for lightweight aggregate is limited.

## Supplementary Material

Appendix 01 (PDF)

Dataset S01 (XLSX)

## Data Availability

All study data are included in the article and/or supporting information.
