# Supplementary material for: Global decarbonization potential of CO2 mineralization in concrete materials
Source: Proc Natl Acad Sci U S A. 2024 Jul 8;121(29):e2313475121. doi: 10.1073/pnas.2313475121 (PMC11260098; doi:10.1073/pnas.2313475121)
Supplement: Supplementary file 1 — Appendix 01 (PDF) [file pnas.2313475121.sapp.pdf]

Supplementary Information S1

Global decarbonisation potential of CO_2 mineralisation in concrete materials

Justin G. Driver [a,1,^], Ellina Bernard [b,c,2,^], Piera Patrizio [d,3,^], Paul S. Fennell [a,4], Karen Scrivener [e,5], Rupert J. Myers [b,6,*]

[a] Department of Chemical Engineering, Imperial College London, Bone Building, South Kensington Campus, London, SW7 2AZ, UK.

[b] Department of Civil and Environmental Engineering, Imperial College London, Skempton Building, South Kensington Campus, London, SW7 2AZ, UK.

[c] Empa, Swiss Federal Laboratories for Materials Science and Technology, Dübendorf, Switzerland.

[d] Centre for Environmental Policy, Imperial College London, Weeks Building, South Kensington Campus, London, SW7 2AZ, UK.

[e] École Polytechnique Fédérale de Lausanne (EPFL), Lausanne, Switzerland.

* Corresponding author. ^ Joint first authors. E-mail: [1] justin.driver@hotmail.com, [2] ellina.bernard@empa.ch; [3] p.patrizio@imperial.ac.uk, [4] p.fennell@imperial.ac.uk; [5] karen.scrivener@epfl.ch, [6] r.myers@imperial.ac.uk.

Table of contents

S1. Summary of methodological approach S4
S2. CO_2 mineralisation feedstock availability and compositions S5
S2.1. CO_2-containing flue gases S5
S2.2. Carbonatable solid materials S5
S2.2.1. Cement kiln dust S6
S2.2.2. Cement bypass dust S7
S2.2.3. Concrete slurry waste S7
S2.2.4. End-of-life concrete S7
S2.2.5. Coal ashes S8
S2.2.6. Iron and steelmaking slags S8
S2.2.7. Air pollution control residues S9
S2.2.8. Incinerated municipal solid waste bottom ash S9
S2.2.9. Paper sludge incineration ash S9
S2.2.10. Phosphogypsum S10
S2.2.11. Glass powder S10
S2.2.12. Bauxite residue S10
S2.2.13. Incinerated sewage sludge ash S10
S2.3. Compositions of carbonatable solid materials S10
S3. Conventional concrete materials S12
S3.1. Fine limestone S12
S3.2. Natural aggregate S12
S3.3. Lightweight aggregate S12
S3.4. Recycled concrete aggregate S13
S3.5. Manufactured aggregate S13
S3.6. Hydrated concrete and mortar S13
S4. CO_2 mineralisation technologies S15
S4.1. Reactive additives S16
S4.1.1. Precipitated calcium carbonate S16
S4.1.2. Cement from carbonated end-of-life cement paste S19
S4.1.3. Composite Portland cement containing clinker from carbonatable solid materials S21
S4.2. Inert additives S23
S4.2.1. Carbonated recycled concrete aggregate S23
S4.2.2. Carbonated lightweight aggregate S24
S4.2.3. Carbonated normal weight aggregate S26
S4.3. CO_2 curing S27
S4.3.1. CO_2 curing of Portland cement concrete (ready-mix) S28
S4.3.2. Carbonated Portland cement concrete products (unreinforced) S28
S4.3.3. Carbonatable calcium silicate cement (concrete products, unreinforced) S30
S4.3.4. Carbonate bonded compacts (concrete products, unreinforced) S31
S5. Demand for CO_2 mineralisation products S32
S5.1. Market segments for concrete materials S32
S6. Climate change impacts of CO_2 mineralisation products S34
S6.1. Goal and scope S34
S6.2. Sensitivity analysis S44

S6.3. Results and discussion S45
S7. Economics of CO_2 mineralisation products S48
S7.1. Scenarios and assumptions S48
S7.2. Economic analysis of cement from carbonated end-of-life cement paste S50
S8. References in this Supplementary Information file S53

S1. Summary of methodological approach

In this paper we combine analysis of material properties, life cycle climate change impacts, supply (generation rates) and demand (market size) constraints, and economic viability of CO_2 mineralisation products and comparable conventional products. Our approach is summarised in Fig. S1 and described comprehensively in this document.

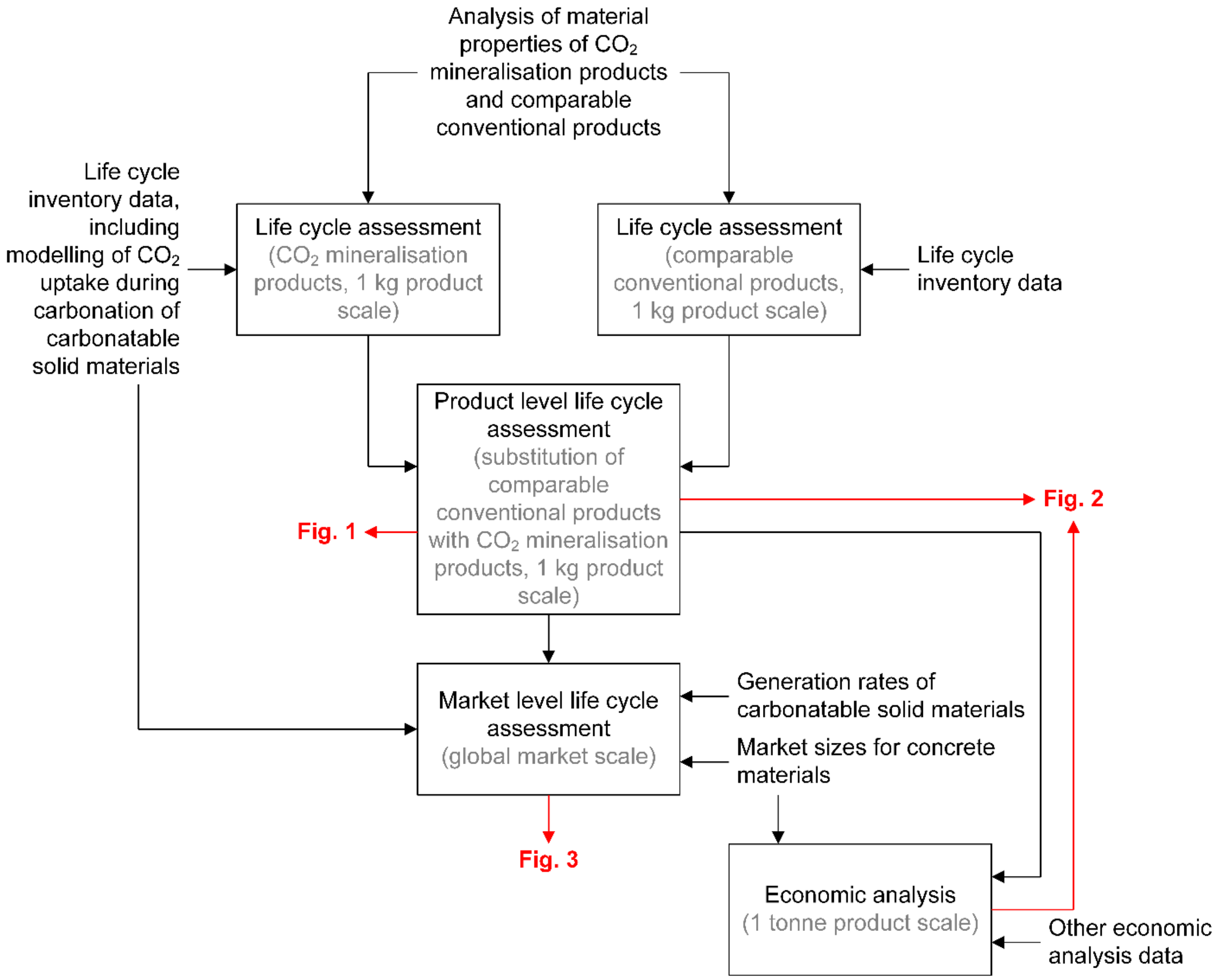

Fig. S1 | Summary of methodological approach used in this paper to investigate the techno-economic and environmental performance of CO_2 mineralisation in concrete materials. The red arrows and text refer to figures shown in the main text.

S2. CO_2 mineralisation feedstock availability and compositions

S2.1. CO_2-containing flue gases

Global emissions of CO_2 peaked in 2018 at 33.6 Gt CO_2, with the most significant fractions from power generation (14.2 Gt CO_2), industry (6.2 Gt CO_2), and transportation (8.2 Gt CO_2), when electricity and heat emissions are allocated to power generation rather than downstream sectors such as industry[1]. Assuming the proportion of emissions from coal (74%), gas (22%), and oil (4%)[1], allows the CO_2 emissions from coal-, gas- and oil-fired power generation to be estimated as 9.9, 2.9, and 0.6 Gt CO_2, respectively. These flues contain concentrated CO_2 that can be used as a feedstock for CO_2 mineralisation technologies[2]. Allocation of electricity and heat demanded by downstream sectors to these sectors results in the total CO_2 emitted by industry of 12.3 Gt CO_2, accounting for just under 40% of global emissions in 2018[1]. Direct emissions from industry reportedly totalled 8.5 Gt CO_2 in 2018 (indirect emissions were ~3.8 Gt CO_2), with significant contributions from the cement (2.3 Gt CO_2), steel (2.1 Gt CO_2), and chemicals (1.2 Gt CO_2) sectors[3-5]. These results are summarised in Table S1.

Table S1 | A selection of the sectors with highest CO_2 emissions in 2018, with typical concentrations of CO_2 in their exhausted flues. Calculated from [1,3-5], adapted from [2].

Sector	Sub-sector	CO_2 emissions (Gt in 2018)	Example processes	CO_2 concentration (vol.%)
Power generation	-	9.9	Coal-fired generation	12 to 15
Power generation	-	2.9	Gas-fired generation	3 to 10
Industrial	Cement	2.3	Cement production	14 to 33
Industrial	Iron & steel	2.1	Steel production	15
			Refineries	3 to 13
			Ethylene production	12
Industrial	Chemicals	1.2	Ammonia production	100
			Hydrogen production	70 to 90
			Natural gas production	5 to 70
Power generation	Oil-fired	0.6	Oil-fired generation	3 to 8
Industrial	Other	2.5	Coal-fired generation	Up to 100
Total (of those listed here)	-	21.6	-	-

The results show that concentrated CO_2-containing flue gases are generated in massive quantities (~21.6 Gt or 64% of global CO_2 emissions in 2018), mostly at large point-source emitters in the power generation and industrial sectors. The properties of these flue gases (e.g. temperature, CO_2 concentration, quantities and types of contaminants) are not expected to be problematic for most CO_2 mineralisation technologies.

S2.2. Carbonatable solid materials

The supply of carbonatable solid materials constrains the scale of CO_2 mineralisation. Since carbonatable CO_2 mineralisation feedstocks must have relatively low upstream CO_2-eq.

emissions to enable systemic reductions in CO_2-eq. emissions (i.e., when used to substitute conventional products), the most studied carbonatable solid materials are industrial by-products and wastes, which are usually allocated low greenhouse gas emissions. Such materials also usually cost less than primary resources and can have few competing uses.

We estimated generation rates for carbonatable solid materials globally using data published between 2015 and 2020, as summarised in Table S2. The calculations for each material are discussed in the respective sections herein, in the Methods Section in the main text, and in Dataset S1. The generation rate and CO_2 uptake potential for cement kiln dust shown in Table S2 are determined assuming no recycling of this material in the cement plant, hence should be considered as upper values.

Table S2 | Global generation rates and CO_2 uptake potentials of carbonatable solid materials in 2020.

Carbonatable solid materials	Global generation rate in 2020 (Gt)	CO_2 uptake potential in 2020 (Gt)
End-of-life concrete	3.27	
… of which is binder	0.74	0.049
End-of-life mortar	2.73	
… of which is binder	0.65	0.043
Cement kiln dust (CKD)	0.20	0.064
Concrete slurry waste (CSW)	0.22	0.054
Cement bypass dust (CBD)	0.02	0.0064
Blast furnace slag (BFS)	0.38	0.13
Basic oxygen furnace slag (BOFS)	0.18	0.059
Electric arc furnace slag (EAFS)	0.09	0.032
Coal ashes (*e.g.* FAs, BAs)	0.68	0.066
Air pollution control residues (APCRs)	0.01	0.0016
Incineration bottom ash (MSW-BA)	0.07	0.012
Paper sludge incineration ash (PSIA)	0.02	0.0095
Phosphogypsum (PG)	0.40	0.088
Glass powders (GPs)	0.11	0.0094
Bauxite residue (BR)	0.15	0.0045
Incinerated sewage sludge ash (ISSA)	0.02	0.0012
Total (excluding non-binder fractions in end-of-life concrete and mortar)	3.93	0.63

S2.2.1. Cement kiln dust

Cement kiln dust (CKD) is generated during the production of cement, and consists of partially calcined meal that is separated from exhaust gases (e.g. bag filters, electrostatic precipitators). It is either utilised on-site or landfilled. CKD is generated at a rate of ∼54-144 kg/tonne Portland clinker under compound operation (or ∼80-200 kg/tonne Portland clinker under direct operation)[6], which is generally reduced from ∼150-200 kg/tonne Portland clinker in recent decades[7]. Global Portland cement (PC) and Portland clinker production were 4.17 Gt and 3.09 Gt in 2020, respectively[4]. Assuming the median rate of generation from above (~127 kg/tonne of

Portland clinker), we estimate CKD to be ~392 Mt in 2020. Fresh CKD is often utilised on-site as recycled kiln feed due to relatively low contamination[6]. Conservatively assuming that 50% of fresh CKD is recycled (up to 100% can be recycled, depending on its chemical composition), we calculate that the amount available is ~196 Mt in 2020. Fresh CKD can be used as a supplementary cementitious material (SCM; i.e., a Portland clinker substitute) since it has not yet been exposed to significant moisture[8].

S2.2.2. Cement bypass dust

Cement bypass dust (CBD) is similarly generated during Portland clinker production, and consists of highly calcined meal that is lost whilst purging contaminants from the kiln bypass (containing mostly Cl, S, Na, and K)[6]. The CBD generation rate is estimated as 15-20 times lower than that for CKD, resulting in the formation of ~3-20 kg/tonne Portland clinker[8]. Using the median generation rate (~12 kg/tonne Portland clinker), we estimate that 35 Mt of CBD was generated globally in 2020. Similarly to CKD, on-site utilisation of CBD can occur but is crucially dependent on the nature and concentration of contaminants. If contamination is sufficiently low (particularly Cl), CBD can be used as recycled kiln feed or a SCM. We assume on-site utilisation of CBD is permissible at the same rate as CKD, reducing the amount available to 18 Mt in 2020.

S2.2.3. Concrete slurry waste

Wastes are also generated during preparation and use of fresh concrete (e.g. poor logistics, ordered surplus, incorrect formulation)[9,10]. In practise, this can mean ∼300 kg/truck of wasted concrete is returned to batching plants[11], where it is often washed to recover coarse aggregate for future batches. The washings containing hydrated cement and fine aggregates are concrete slurry waste (CSW). CSW is typically processed through sedimentation to recover water, which is often recycled as concrete mix water, and co-producing a more concentrated slurry that is in many cases landfilled.

CSW generation is estimated at ~0.8% of concrete production[12]. Using this value, cement production data, and assuming a mean value for the amount of cement in concrete of ~13.2%, we estimate the generation rate of CSW of 254 Mt in 2020.

The concentrated CSW by-product is considered a hazardous waste due to its basicity (pH ≥ 11.5)[10] and is typically poorly unutilised. Although there is significant hydration of Portland clinker in CSW from washing[9,12], we expect that carbonation of this material could generate a SCM suitable for Portland clinker substitution.

S2.2.4. End-of-life concrete

Concrete has a lifetime that can span many decades depending on its service conditions. Eventually however, buildings and infrastructures are demolished, generating ‘end-of-life’ concrete and mortar. At the time of generation, end-of-life mortar is likely to be highly carbonated since it is porous, whereas end-of-life concrete in typical applications is dense and mostly uncarbonated. End-of-life concrete forms a major fraction (~58%) of construction and

demolition waste (CDW)[13], alongside other building materials (e.g. asphalt, bricks, masonry) and excavated soils[14]. Of particular interest is the cementitious binder (i.e., cement paste) in end-of-life concrete since it can be processed into a Portland clinker substitute material[15].

We used data from [16] to calculate the potential generation rate of end-of-life cement in 2018 (0.82 Gt). Assuming the global average consumption of cement in concrete (60%) and mortar (40%) is approximated by cement use in Brazil[17], and typical water-to-cement mass ratios of 0.5 and 1 for concrete and mortar respectively, generation rates of end-of-life binder in concrete (0.74 Gt/year) and mortar (0.65 Gt/year) were calculated. These values were then combined with typical cement fractions of 15% and 12% in concrete and mortar to obtain generation rates of end-of-life concrete (3.27 Gt/year) and mortar (2.73 Gt/year) in 2018, which we use here.

The recycling rate of CDW (and the end-of-life concrete therein) varies globally, with some countries recycling the majority of wastes (*e.g.* 95-98% in USA, Canada, Japan, South Korea, many EU countries)[18] and others without significant recycling (*e.g.* Mexico, South Africa, and a few EU countries)[14,18]. We assume 100% recovery of end-of-life concrete and mortar and of end-of-life cement paste from those materials, meaning the global generation rates shown here are upper limits. Industrial separation of end-of-life concrete into cement paste and aggregate is emerging[19,20]. At present, end-of-life concrete is mainly recycled into ‘loose’ construction applications (e.g. structural fills, backfills, embankments, etc.)[14], and increasingly as ‘recycled concrete aggregate’ (RCA) in concrete. Although complete replacement of conventional normal weight aggregates (NWA; e.g. gravel) with RCA is unlikely due to the superior physical properties of the former, RCA is nonetheless suitable for carbonation into carbonated recycled concrete aggregate (CRCA) and/or a wide range of mainly non-structural applications.

S2.2.5. Coal ashes

Demand for coal was 5.45 Gt in 2018[21]. The generation rate of ash varies greatly based on the quality of the coal, but is generally between ~50-200 kg/tonne coal[22]. The majority (70-80%) of this ash is transformed into fly ash (FA), and a minority (10-20%) into bottom ash (BA)[22]. Here, we do not distinguish between coal FAs and BAs as this does not affect the calculation of their total generation and CO_2 uptake (especially since they have broadly similar oxide compositions[23]). Assuming a median ash content (~125 kg/tonne ash) indicates that global production of coal ashes was 681 Mt in 2018.

S2.2.6. Iron and steelmaking slags

A variety of slags are generated in iron and steelmaking processes. Global production of iron is reported at 1.40 Gt in 2019[24]. Blast furnaces (BF) account for 91% of iron production in 2020, however this value is forecasted to decrease to 52% of production in 2050 due to adoption of direct reduced iron (DRI) (from 8 to 32%) and smelting reduction (SR) (from 1 to 16%)[24]. Slags are generated from BFs at ~300 kg/tonne iron[25], suggesting the production of blast furnace slag (BFS) will decrease from 383 Mt in 2019 to 199 Mt in 2050. Currently, much of the blast furnace slag that is generated is used as a SCM in composite PC (estimated at >90% utilisation).

Globally, 1.9 Gt of steel was produced in 2019[5]. Basic oxygen furnaces (BOF) account for 71% of production in 2019, with predictions that this will fall to 43% in 2050, with corresponding increases in electric arc furnace (EAF) based production (from 29% to 57%)[5]. Basic oxygen furnace slag (BOFS) is generated at ~130 kg/tonne of crude steel, whereas this value is ~170 kg/tonne for electric arc furnace slags (EAFS)[25]. Assuming these rates remain constant over the period, we calculate the generation rate of BOFS as 175 and 112 Mt in 2019 and 2050, respectively, with the corresponding values for EAFS as 94 and 194 Mt. We note that this calculation neglects nuanced changes to the production route (e.g. increased slag generation in EAFs as a direct result of no slag removal from DRI) and the feed composition (e.g. decreased slag generation due to higher input of scrap steel)[5].

S2.2.7. Air pollution control residues

A variety of acidic gases (e.g. SO_x, HCl, HF) may be generated during combustion which require removal by flue gas treatment (FGT)[26]. This often involves injection of CaO or $Ca(OH)_2$ which neutralise these species by forming solids (e.g. $CaSO_4$, $CaCl_2$, CaF_2) that are separated from the flue[26]. These solids are broadly referred to as air pollution control residues (APCRs), and typically result from fuels with relatively high contamination (e.g. waste incineration). We estimated the global generation of APCRs from the corresponding amount of incinerated municipal solid waste (MSW), which has been reported as 2.0 Gt in 2016 and projected to be 3.4 Gt in 2050 [27].

In some countries, incineration is the major disposal route for MSW (e.g. 50-70% in Japan, Denmark, Sweden, Switzerland) whereas others engage in less incineration (e.g. 0-12% in Canada, Australia, Turkey)[28]. Globally, an average of only ~11% of MSW undergoes incineration[27], with the generation of APCRs at ~15-40 kg/tonne of incinerated MSW[26]. Assuming disposal by incineration remains constant over this period and taking the median rate (~28 kg/tonne of incinerated MSW) suggests global generation rates of 6.1 and 10.3 Mt of APCRs in 2016 and 2050, respectively.

S2.2.8. Incinerated municipal solid waste bottom ash

Incineration of MSW also generates considerable amounts of bottom ash (MSW-BA), at up to ~300 kg/tonne of incinerated MSW[26]. Applying the same assumptions as used for APCRs (Section S1.2.7)[27], we calculate that the global production of MSW-BA was 67 Mt in 2016 and increases to 112 Mt in 2050.

S2.2.9. Paper sludge incineration ash

Large amounts of sludge are generated during paper production, a fraction of which is often combusted to produce paper sludge incineration ash (PSIA). Paper consumption has been reported as 399 Mt for 2020 and projected as 461 Mt in 2030[29]. Paper sludge is reportedly generated at ~40-50 kg/tonne of paper[30], with the PSIA produced at 8-16 kg/tonne of incinerated sludge[31,32]. Taking the median values for the paper sludge (~45 kg/tonne) and ash (~12 kg/tonne) rates respectively, suggests the amount of PSIA generated in 2020 was 22 Mt.

S2.2.10. Phosphogypsum

Phosphogypsum (PG) is generated from phosphate rocks during the production of phosphoric acid and phosphate fertilisers[33], at a rate of 4-5 tonnes/tonne of phosphoric acid[33]. Given global phosphoric acid production was 87 Mt in 2019[34], and assuming the median rate (~4.5 tonnes/tonne phosphoric acid) we estimate that ~395 Mt of PG was generated in 2020. We note that long-term depletion of naturally occurring sources will require greater recovery of phosphate from alternative sources (*e.g.* manure, excreta, residues), which will not necessarily generate PG, so we expect its generation rate to decrease in the future[35].

S2.2.11. Glass powder

Glass powders (GP) are generated during the manufacture of glass, typically from wastage during recycling of cullet and/or end-of-life glass. Total glass consumption was reportedly 128 Mt in 2018[36]. The recycling rate varies greatly by region and product-type but a global average of ~21% was reported in 2018[36], which implies a current upper limit of ~79% is available for processing into GP. We thus estimate that 109 Mt of GP was generated in 2020. This is likely an overestimate since glass is easily recyclable and certain products can be made almost entirely from cullet (~90% for green container glass)[37], further evidenced by high recycling rates in some regions (*e.g.* >70% in much of the EU). Since the CaO content in glass is low, overestimating its generation rate will not significantly affect the potential overall CO_2 uptake by carbonatable solid materials that we quantify here.

S2.2.12. Bauxite residue

Refining of bauxite ores into alumina is an initial step toward the production of aluminium metal. Some estimates give global alumina production as 124 Mt in 2020[38]. During refining, bauxite residue are generated in significant quantities often between 0.9-1.5 tonnes/tonne of refined alumina[39,40]. Applying the median value (~1.2 tonnes/tonne) that global generation of bauxite residue was 149 Mt in 2020.

S2.2.13. Incinerated sewage sludge ash

Incinerated sewage sludge ash (ISSA) is generated from the disposal of hazardous sludges produced in wastewater treatment plants. Estimated global water consumption projections suggest values of 1,487 and 1,847 Gt for 2020 and 2050 (calculated from linear extrapolation of predictions for 2025 and 2040 in 2014[41]). Assuming only ~18% of this water is recovered for treatment (i.e. the cumulative fraction from municipal, industrial and power generation sources[41]) reduces the maximum quantity of wastewater processed. The generation rate for dewatered sludge has been estimated at ~0.182 kg/tonne of wastewater influent[42], with the formation of ISSA between 230-470 kg/tonne of dewatered sludge (assuming a median of ~350 kg/tonne)[43-45]. Altogether, this estimates the global production of ISSA at 17 and 21 Mt in 2020 and 2050, respectively. ISSA can be used as a feedstock for carbonation, however this might compete with its relatively high value as an agricultural fertiliser due to its significant phosphorus content [35].

S2.3. Compositions of carbonatable solid materials

Compositions of carbonatable solid materials were reviewed from literature to estimate their CO_2 uptake capacities (see Dataset S1). Compositions were given as the principal oxide for each major element, namely for calcium (CaO), silicon (SiO_2), aluminium (Al_2O_3), iron (Fe_2O_3), magnesium (MgO), potassium (K_2O), sodium (Na_2O), phosphorus (P_2O_5), and sulphur (SO_3), in addition to chlorine (Cl) and loss on ignition (LOI). We estimated the oxide compositions of end-of-life in concrete and mortar using those for recycled concrete aggregate due to the lack of reported data for the former materials.

We estimated the potential CO_2 uptake for each carbonatable solid material considering CO_2 uptake in CaO only, and assuming half of the loss on ignition is CO_2 (the remainder is mainly H_2O). We exclude carbonation of (i) SO_3, since carbonation of $CaSO_4$ would lead to SO_x emissions; (ii) Na_2O and K_2O since their carbonate salts are highly soluble; and (iii) MgO since it is more difficult to carbonate MgO than CaO at room temperature. We made this estimation to reduce the complexity of calculations in this paper.

We compared the potential CO_2 uptake (g) estimated using the assumption that half of the loss on ignition is CO_2 to data reported in the literature (Fig. S2). The calculations are shown in full in Dataset S1. The results show that the assumption that half of the loss on ignition is CO_2 is valid since it yields comparable potential CO_2 uptake values to those reported in the literature for many carbonatable solid materials.

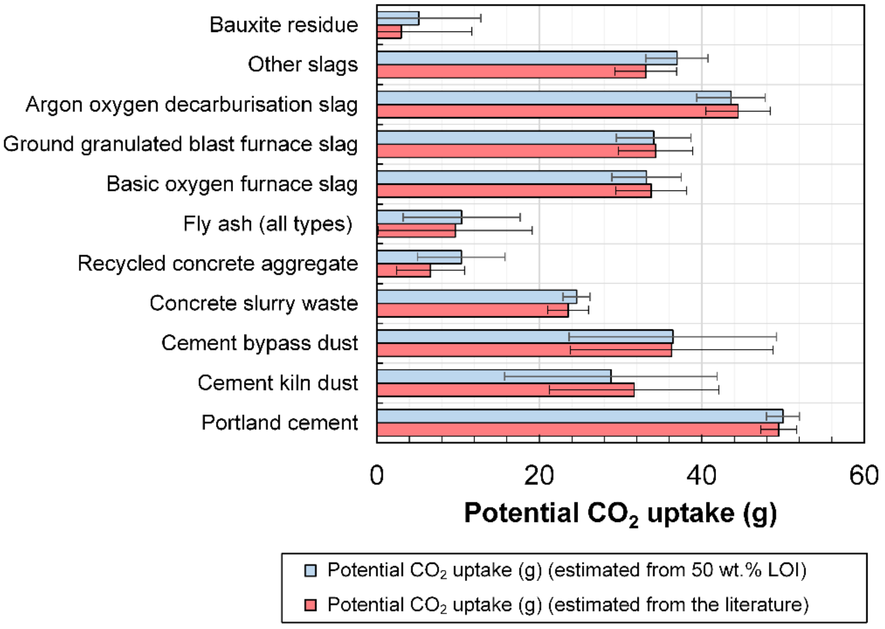

Fig. S2 | Potential CO_2 uptake (g) estimated assuming that the half of the loss on ignition is CO_2 (this study) and using data reported in the literature.

S3. Conventional concrete materials

This section describes conventional concrete materials that CO_2 mineralisation technologies can substitute.

S3.1. Fine limestone

In cement production, primary limestone (calcium carbonate sedimentary rock) is usually ground to produce 'fine limestone', which is then blended with Portland clinker in cement.

According to European standard (EN 197-1)[46], limestone used as additive in cement production shall contain between 75-95 wt.% $CaCO_3$, which is a relatively low purity that allows the presence of clay and other impurities (e.g. quartz). The standard additionally requires such limestone to have a low moisture content, and the following chemical composition: SO_3 <1 wt.%; MgO, <3 wt.%; P_2O_5, <1 wt.%; and $Na_2O + K_2O$ <1 wt.%. The limiting content of MgO (<3 wt.%) was set to avoid the portlandite-dolomite reaction that potentially induces expansion; however, recent studies indicate that this reaction is not necessarily expansive[47,48].

Limestone ($CaCO_3$) can be used as the sole SCM in CEM II/L or CEM II/LL (substituting PC clinker by up to 35 wt.% in the latter case), or in addition to aluminosilicate SCMs (e.g., coal fly ash, granulated blast furnace slag) in composite cements (CEM II and CEM IV)[49]. Since in small quantities limestone is reactive in cement binders, it is considered to be a component of the binder (i.e. the cement 'glue' that sticks aggregates together in mortar and concrete). The reactivity of fine limestone depends on the alumina content of the binder and the fineness of the limestone used[50], with more alumina rich binders and finer particles being more reactive[51-53]. Fine limestone additions are typically beneficial (or at least not detrimental) to the mechanical properties of the concrete when it replaces less than 15 wt.% of the PC clinker[54-58]. Additions of fine limestone above 15 wt.% of PC clinker effectively act as PC clinker diluents.

S3.2. Natural aggregate

Most aggregates used in concrete are quarried from primary resources. Such aggregates are called natural aggregate. They usually have a 'normal' density of 2.4-2.8 g/cm^3. Fine aggregates (sand) correspond to particles with diameter ≤4 mm while coarse aggregate (gravel) are particles with diameter ≥4 mm. The composition of the aggregates varies between quarries but their use is standardised in concrete based on their size, density, alkali-aggregate reactivity, and water uptake[59]. Aggregate compositions can vary from siliceous (e.g. presenting large amount of quartz, feldspar, mica) to calcareous (e.g. presenting calcite and dolomite), although can often contain both siliceous and calcareous fractions. Natural aggregate also refers to crushed natural rocks, and here crushing can produce coarse and/or fine aggregates.

S3.3. Lightweight aggregate

Natural aggregates can also have low density (classified as 'lightweight'). Natural lightweight aggregate exists in the form of volcanic pumice, however, the majority of lightweight aggregate is produced from natural dense clays, rocks, or wastes by a granulation, pelletization, and sinter-

hardening method (>1,000 °C). Prior to pelletization, the wastes are soaked to produce a slurry after which they can be formed into more regularly shaped particles/pellets suitable for sintering. The sintering method consists in fusing particles of fresh pellets together by increasing the temperature[60]. Generally, lightweight aggregates have a density of 0.8-2.0 g/cm^3 and can provide good thermal and acoustic insulation, and fire resistance. Therefore, lightweight aggregate is suitable for use in concrete blocks and lightweight (including structural) concrete, but cannot completely replace natural aggregate in structural concrete due to their lower density and compressive strength.

S3.4. Recycled concrete aggregate

Due to the large annual demand of aggregate, there is increasing pressure to recycle end-of-life concrete into (secondary) aggregates, called recycled concrete aggregate (RCA), which can be produced using crushing. Recycling rates and available applications for RCA vary widely around the world. Crushed concrete contains natural aggregate mixed with aggregate/mortar and has an overall density between 2.0-2.4 g/cm^3.

Usually, the fine fraction of RCA (mainly composed of sand and cement paste) is not recommended for production of concrete due high water absorption[61]. This high water absorption decreases workability, which in turn increases water demand, or cement or superplasticiser content, in RCA-containing concrete. For the same reasons, the replacement of coarse natural aggregate by coarse RCA in structural concrete is still limited due to its inherent content of cement paste (hydrated and partially carbonated).

Natural and recycled concrete aggregates are usually compared by the performance of their respective concretes. At equivalent transportation and similar compressive strengths, Marinkovic et al. (2010) reported 100-year global warming potentials of 308 and 343 kg CO_2-eq. for the production of 1 m^3 PC concrete containing natural and recycled concrete aggregates, respectively[62]. Consistent with this result, it has been reported that significantly lower transport distances (e.g. ~170 km [17]) are required for RCA-containing concrete to have a lower CO_2 footprint than conventional natural aggregate concrete.

S3.5. Manufactured aggregate

Another type of aggregate is manufactured aggregate, which refers to aggregates produced from industrial wastes/by-products. These materials can be derived from carbonatable solid materials like coal fly ash. Their properties depend mainly on the manufacturing process and the industrial wastes/by-products used and can be produced to have lower density (lightweight aggregates, LWA) or ‘normal’ density (normal weight aggregates, NWA). Manufactured aggregate produced from carbonatable solid materials can be carbonated, which can improve their material properties. We focus on these technologies (carbonation processes) to produce carbonated manufactured aggregates in this paper.

S3.6. Hydrated concrete and mortar

Concrete and mortar are usually mixed with water (‘hydrated’) and then cured under an ambient environment (room temperature, atmospheric pressure, and exposed to air). This converts the clinker phases (i.e. alite, belite, etc.) into hydrated phases, which serve as the binding (‘glue like’) phases in concrete. Alternatively, CO_2 can be used to cure concrete (‘CO_2 curing’), resulting in the formation of carbonated binding phases (both in the presence and absence of water), and we explore these technologies here.

S4. CO_2 mineralisation technologies

We consider a wide range of CO_2 mineralisation products in our analysis (Fig. S3), including:

i. Reactive additives (within concrete materials), which are fine particulate carbonated minerals and can be used to substitute clinker;
ii. Inert additives, which are coarse or fine aggregates, including non-reactive fillers, and can be used to substitute aggregate in mortar and concrete; and
iii. CO_2 curing, whereby CO_2 is directly added into fresh concrete and functions as a strength accelerator or as an intrinsic component of the binding phase.

Additionally, we include composite PC with clinker from carbonatable solid materials since this technology is a straightforward way to exploit these materials (as substitutes for primary limestone) and could limit their supplies for other forms of CO_2 mineralisation. We exclude reinforced concrete (except CO_2 injection into ready-mix concrete) owing to the increased risk that carbonation of the concrete cover layer induces in corrosion of embedded steel reinforcing bar, which is problematic for structural applications.

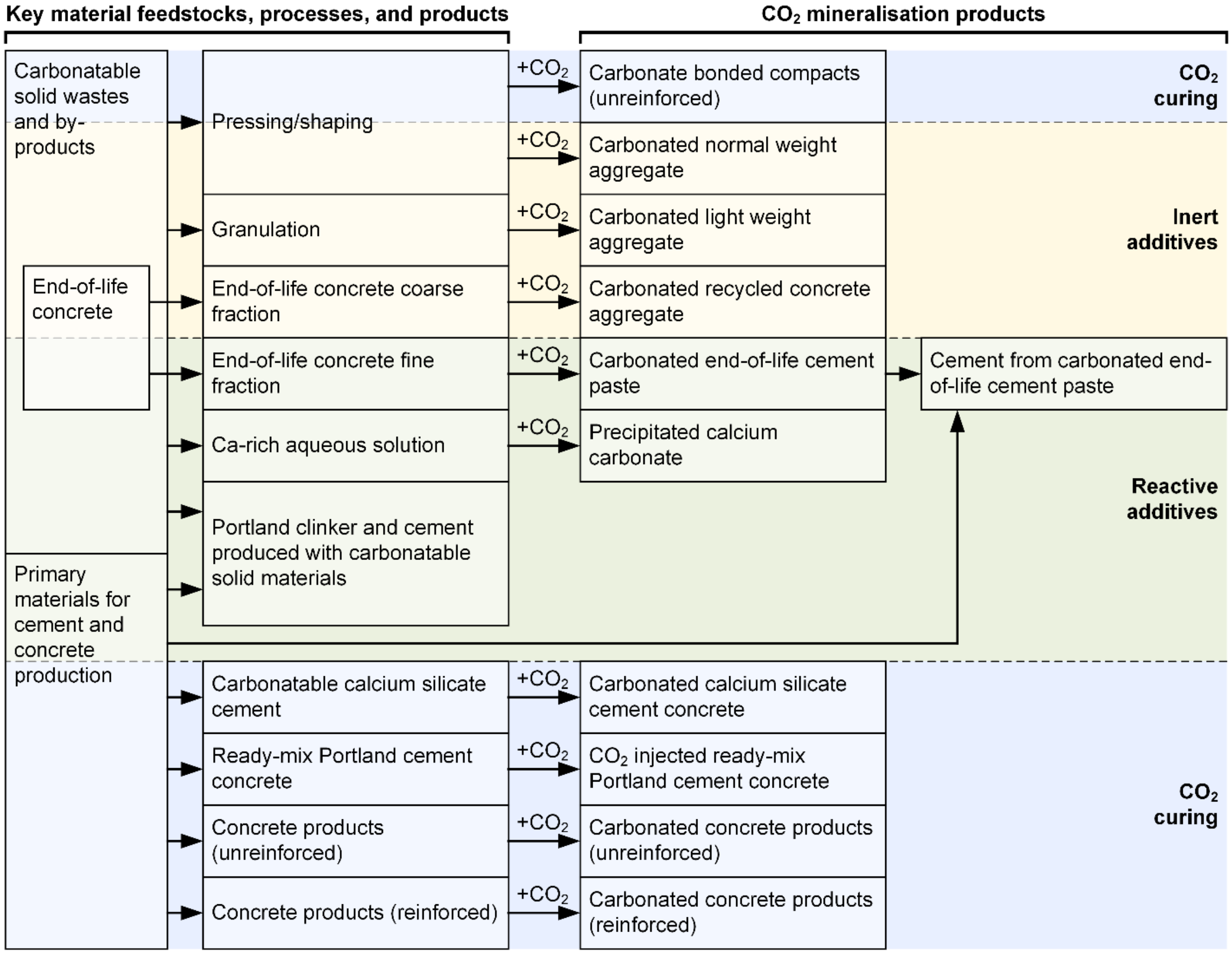

Fig. S3 | Material feedstocks, processes, and products for CO_2 mineralisation in concrete materials. The CO_2 mineralisation products shown are analysed in this paper. Clinker and cement produced with carbonatable solid materials is not a CO_2 mineralisation product but

competes for use of the same feedstocks, so is also considered here. The CO_2 mineralisation products are classified as either inert additives, reactive additives, or CO_2 curing depending on their vertical position in the figure. The arrows represent distribution and transformation of carbonatable solid materials through processes and into products.

S4.1. Reactive additives

S4.1.1. Precipitated calcium carbonate

S4.1.1.1. Production process

Precipitated calcium carbonate (PCC) is produced from pure CaO, $Ca(OH)_2$, or other CaO sources [63]. Since naturally occurring CaO sources are extremely rare at the Earth's surface, most CaO for PCC production is usually an intermediate product produced from rocks (e.g., limestone or calcium silicate rocks [64], although the availability of the latter is low compared to the former). Alternatively, CaO can be sourced from industrial by-products/wastes [65]. For the extraction of CaO or $Ca(OH)_2$ from these materials, acid treatment is usually applied as the first step, followed by a basic treatment, and then precipitation of high purity $CaCO_3$ (PCC) [66]. Production of PCC from Ca-containing alkaline wastes can be achieved using 'wet' and 'dry' processes.

In the LCA study conducted in this paper, we model PCC production using the wet process (Fig. S4). We chose an optimistic case where the PCC is produced from carbide sludge which is 70 mass% CaO and 25 mass% LOI – few industrial by-products have as high CaO content. This process involves dissolving Ca from the Ca-containing alkaline wastes (here they are the 'carbonatable solid materials', e.g. carbide sludge as in [67]) into an acidic Ca-containing solution (e.g. using NH_4Cl as the acidic extraction agent); separating this solution from the solid residues (e.g. using filtration); dissolving waste CO_2 (using ammonium salts [67,68]); precipitation of calcium carbonate; and additional filtration/drying steps. The extracting agent is recycled with a yield of 95%. The total energy demand is estimated to be 3.9 MJ/kg [67,68]. We assume that this energy demand, which is for mechanical processing and drying, can be met with electricity (1.1 kWh).

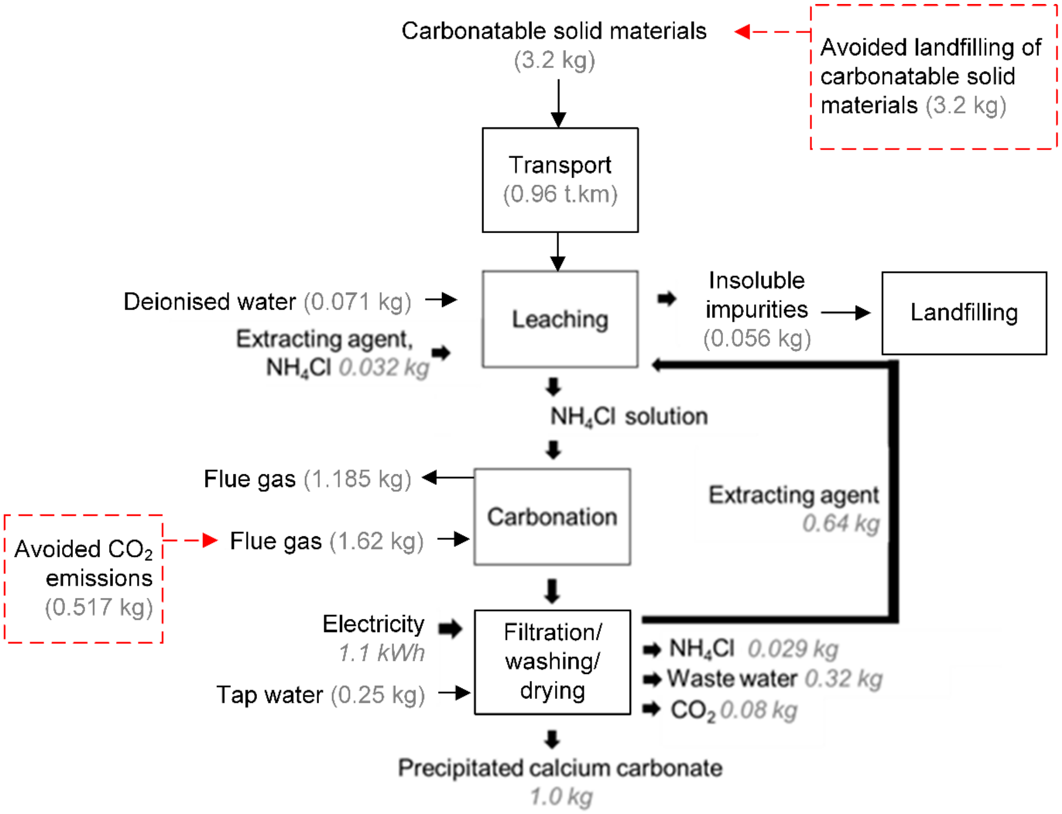

Fig. S4 | Flowchart for precipitated calcium carbonate production from carbonatable solid materials and CO_2-containing flue gas. Environmental credits modelled in this study are indicated by red dashed lines: We model carbonatable solid materials as burden free at their points of generation, the use of carbonatable solid materials as avoided landfilling, CO_2 in feedstock flue gas as waste, and all the CO_2 in feedstock flue gas that is absorbed by CO_2 mineralisation products as avoided CO_2.

S4.1.1.2. Material properties

PCC usually has high purity (>97 wt.%) but contains traces of $MgCO_3$, Al_2O_3, Fe_2O_3, SiO_2, and sulphates. The impurity content depends on their concentrations in the feedstock materials used and/or the process used to dissolve calcium into solution (Table S3). For PCC produced from carbonatable solid materials to be used in concrete, it is important that these feedstock materials have low heavy metal content so that the produced concrete is non-hazardous.

Table S3 | Properties of PCC and processes used to produce PCC from various Ca-containing by-products/wastes, adapted from [69]. n.d. = not defined.

Raw materials	Extraction agent	CO_2 concentration/ temperature/ pressure	PCC characteristic	Ref.
Basic oxygen furnace slag	NH_4Cl aqueous solution	No data / 40-80 °C / 10-40 bar	- 95% $CaCO_3$,<br>- Impurities: crystallite $MgCO_3$, Al_2O_3, Fe_2O_3, SiO_2,<br>- Formation of agglomerates,	70

			- Increase of the carbonation temperature results in changes of morphology	
Steel converter slag	Aqueous acetic acid solution (1M)	10-100 % / 30 & 70 °C / 1 bar	- Calcite forms,<br>- Very high ISO-brightness (98.7%),<br>- Mean particle size of 0.6 um,<br>- Rhombohedral shape.	71
Steel converter slag	Ammonium salt solution	10-100 % / 20 & 70 °C / 1 bar	- Formed product is a 98.7% pure $CaCO_3$,<br>- Formation of vaterite.	72
End-of-life concrete	NH_4Cl, HCl, CH_3COOH, water	100% / 25 °C / 1 bar	- Mixture of rhombohedral calcite and spherical vaterite,<br>- Agent extraction type does not affect $CaCO_3$ precipitation.	73
Concrete sludge (waste cement)	Water	6-13 % / 25 °C / 1 bar	- High-purity $CaCO_3$ (>97%),<br>- Contaminants: SO_3 (1.2% wt.), Al_2O_3 (0.09% wt.), SiO_2 (0.08% wt.), Fe_2O_3 (0.04% wt.), MgO (<0.01% wt.).	74
Concrete sludge (waste cement)	Water	8-13 % / 25 °C / 1 bar	- Calcite form,<br>- High-purity $CaCO_3$ (>99%),<br>- Contaminants: silicon, magnesium<br>- Particles size in the range of 3–30 um,<br>- Average particle size of 10 um.	75
Concrete sludge (waste cement)	Carbonic acid solution (pressurized CO_2)	No data / 30-70 °C / 1 bar	- With addition of seed crystals: high-purity $CaCO_3$ (>98%),<br>- Without addition of seed crystals: $CaCO_3$ with 80% purity.	76

The main difference in the PCC materials produced via this route is its crystal structure and thus the stability of the $CaCO_3$ polymorph formed (i.e. vaterite, calcite, or aragonite). The type of polymorph formed depends on both the concentration of CO_2 in the flue gas and the reaction temperature. Low CO_2 concentrations and near ambient temperatures result in calcite as the main reaction product, while higher CO_2 concentrations and temperatures promote vaterite and aragonite formation. Since vaterite is a thermodynamically metastable calcium carbonate phase relative to calcite and aragonite (depending on the CO_2 content in the gas phase), it is an especially reactive additive. Depending on the production process, the mean particle size of PCC can be smaller (<1 µm)[71], or larger (1-30 µm)[75].

In general, we expect PPC to have similar or improved reactivity relative to fine limestone as a SCM since it will typically be purer and have a finer particle size distribution, resulting in concrete with at least similar or improved compressive strength development. We expect vaterite-containing PCC to be somewhat more reactive than fine limestone, and thus its utilization to give improved compressive strength development.

PCC represented 25% of the calcium carbonate products market in 2010-2012 [64], mainly being used as a high purity feedstock for production of materials such as paper and plastic, but excluding building and construction where natural carbonated rocks are usually used. Despite its

suitability as a SCM, a major drawback from using PCC rather than ground limestone is its higher production cost.

S4.1.2. Cement from carbonated end-of-life cement paste

S4.1.2.1. Production process

End-of-life concrete contains aggregate and end-of-life cement binder (equivalently 'paste'). Carbonation of end-of-life cement paste can produce a reactive material that can be used as a SCM. We refer to this SCM as 'carbonated end-of-life cement paste' (CCP). For end-of-life concrete containing CEM I, complete carbonation of the end-of-life cement paste leads to CCP containing ~70 wt.% calcite, ~20-25 wt.% of an aluminosilicate phase, and ~5-10 wt.% of poorly reactive phases. CCP cement is produced by mixing CCP (38 mass%) with Portland clinker (57 mass%) and gypsum (5 mass%) [77]. Both the calcite and aluminosilicate phases are potentially reactive in CCP and therefore in CCP cement (i.e. containing Portland clinker, CCP, and calcium sulfate).

There are two main types of processes used to carbonate end-of-life cement paste: dry and wet. Here, we model CCP cement production using the wet (1 step) process, which has been shown to achieve high conversion relatively quickly (30 minutes to 2 hours) at ambient temperature and pressure, independent of the CO_2 concentration in the gas phase (i.e. a CO_2 concentration of 10-30% is suitable, which is what is present in cement plant flue gas) [78-80]. In wet carbonation, the CO_2 concentration also has a limited influence on the reaction mechanism and on the products (i.e. it results mainly in calcite and aluminosilicate products [78]). This contrasts with the dry process, where carbonation is enhanced at higher CO_2 concentrations.

In this study, we model the wet process as presented in Fig. S5. To obtain 1 kg of CCP, 5.65 kg end-of-life concrete and 1.03-1.96 kg of flue gas are required. We consider the end-of-life cement paste in feedstock end-of-life concrete material to be partially carbonated (we assume that 18% of the end-of-life cement paste carbonates during the lifetime of a building and 15% during demolition and storage = 33% total carbonation extent), and that end-of-life concrete contains 13.2 mass% end-of-life cement paste, 60 mass% of the end-of-life cement paste is CaO, and the carbonation process converts 100% of the uncarbonated CaO in the feedstock material to $CaCO_3$. This feedstock material to the carbonation reactor, which is the finest fraction output by grinding end-of-life concrete (at least one coarser fraction is also produced), is assumed to contain 67 mass% end-of-life cement paste and 33 mass% fine aggregate. Overall, this process involves crushing (end-of-life concrete), separation (of the ground product based on particle size), carbonation (of the residual solids via the wet process), filtration/drying (to extract the carbonated solids), and blending with Portland clinker and gypsum to produce cement from CCP.

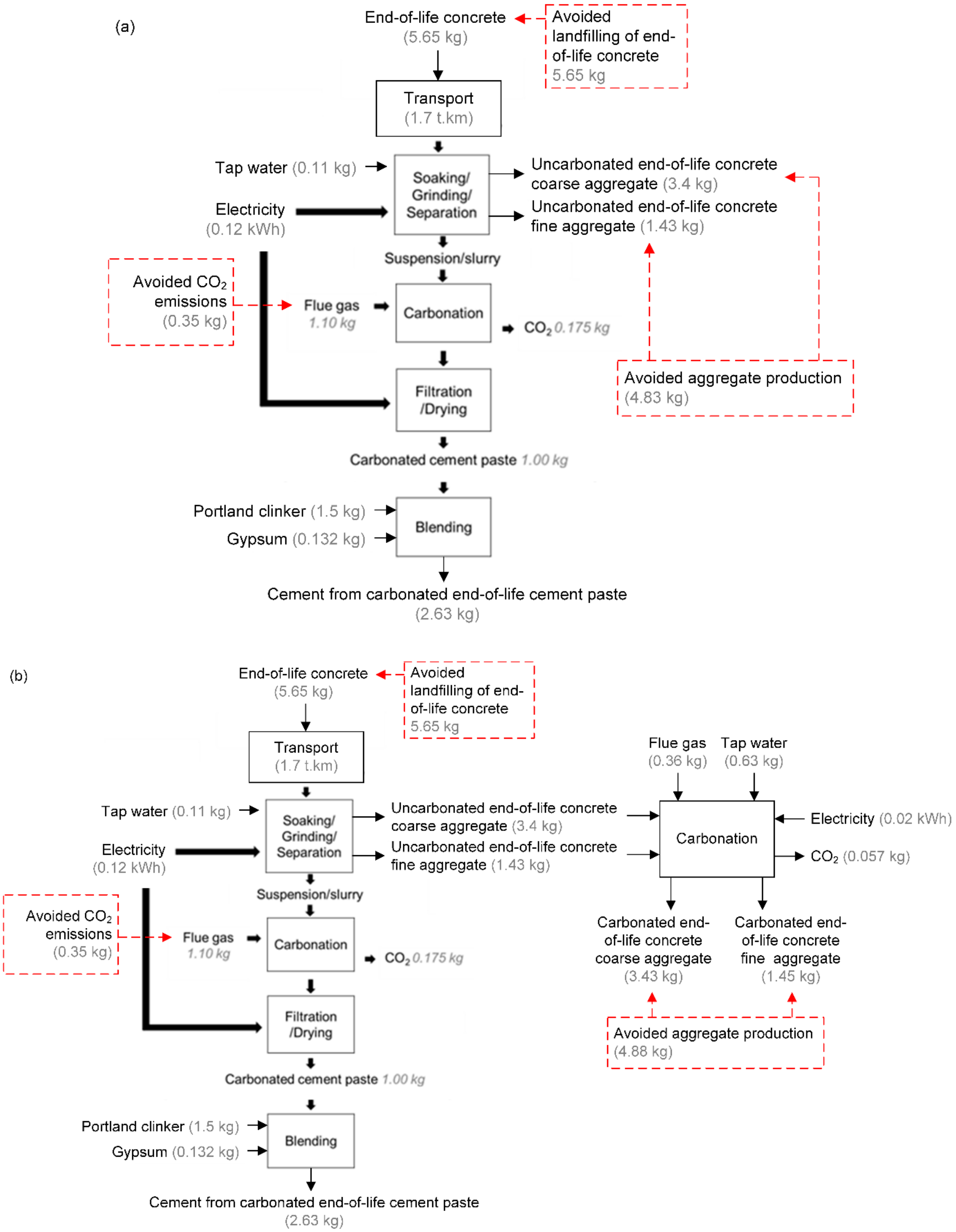

Fig. S5 | Flowcharts of wet (1-step) processes for the production of cement from carbonated end-of-life cement paste (CCP), (a) excluding and (b) including carbonation of aggregate by-products. Electricity is used to power fans, drive assembly and mills. Environmental credits modelled in this study are indicated by red dashed lines: We model carbonatable solid materials

as burden free at their points of generation, the use of carbonatable solid materials as avoided landfilling, CO_2 in feedstock flue gas as waste, all the CO_2 in feedstock flue gas that is absorbed by CO_2 mineralisation products as avoided CO_2, and the use of carbonated aggregates (CO_2 mineralisation co-products) as avoided aggregate production.

We perform a sensitivity analysis on cement from CCP by considering carbonation of the separated aggregates from end-of-life concrete, which contain some adhered cement paste (Fig. S5b).

S4.1.2.2. Material properties

Recent studies have shown that the reactive fractions (calcite and the aluminosilicate phase) in CCP have small particle sizes and the aluminosilicate phase has high pozzolanic reactivity [15,78,81]. Carbonation at high pH and alkali concentration precipitates larger calcite crystals and incorporates calcium and alkali in the aluminosilicate phase.[82] This aluminosilicate phase is reported to be amorphous and very fine, indicating a higher reactivity than other common SCMs (e.g. coal fly ash). The compressive strength of mortar made with cement from CCP has been reported to have similar or increased compressive strength than a cement similar to CEM II/B-L (57 wt.% PC clinker, 38 wt.% limestone, and 4 wt.% anhydrite) and CEM II/B-M (57 wt.% PC clinker, 29 wt.% coal fly ash, 10 wt.% limestone, 4 wt.% anhydrite) between 1 and 180 days of curing [77]. Therefore, relatively high Portland clinker substitution extents are possible using CCP, and at least comparable performance to conventional composite PC (35% clinker) is possible.

Further studies on CCP are required to account for the evolution of concrete compositions over time. For example, end-of-life cement paste (as a constituent of end-of-life concrete) is currently generated from buildings constructed many decades ago (~70 years). This material is similar to hydrated PC, and its processing yields CCP containing mainly calcite and pozzolanic silicate. However, end-of-life cement paste will contain additional aluminate in the future from the widespread use of SCMs, which may change its chemistry and thus reactivity.

S4.1.3. Composite Portland cement containing clinker from carbonatable solid materials

S4.1.3.1. Production process

Carbonatable solid materials can also be used as clinker feedstock materials to produce Portland clinker and composite PC. Although this technology does not utilise waste CO_2, we consider it here since it competes with CO_2 mineralisation technologies for carbonatable solid materials and reduces the amount of limestone fed into the cement kiln (and subsequently the associated CO_2 emissions). This technology involves partial substitution of the clinker raw materials, e.g. calcareous marl (~70 wt.% limestone and ~ 30 wt.% clays), since similarly to marl, carbonatable solid materials have significant SiO_2 (and potentially also Al_2O_3 and Fe_2O_3) content.

Fig. S6 shows the production process for composite PC containing clinker from carbonatable solid materials. We modified the ecoinvent v.3.8 unit process for clinker production (clinker production, CH; UUID be6ec015-2bd3-3199-9bbb-9555a2f7c046) to derive the unit process data for this technology. We assumed that the carbonatable solid materials contain negligible loss on ignition, i.e., no CO_2 or H_2O, and minimal heavy metal content, which is the case for a number

of these materials e.g. coal fly ash. Hence we calculate that the total content of calcareous marl (0.39 kg) can be substituted by 0.25 kg carbonatable solid materials (which thus avoids the CO_2 that would have been emitted from pyroprocessing this substituted calcareous marl), which represents a substitution of 26 wt.% of the clinker feedstock. This is approximately the maximum possible substitution extent of clinker feedstock to achieve the target clinker composition. To this, we model the addition of bauxite (0.003 kg), limestone (1.16 kg), and other inputs (see Dataset S1) to the kiln to produce Portland clinker.

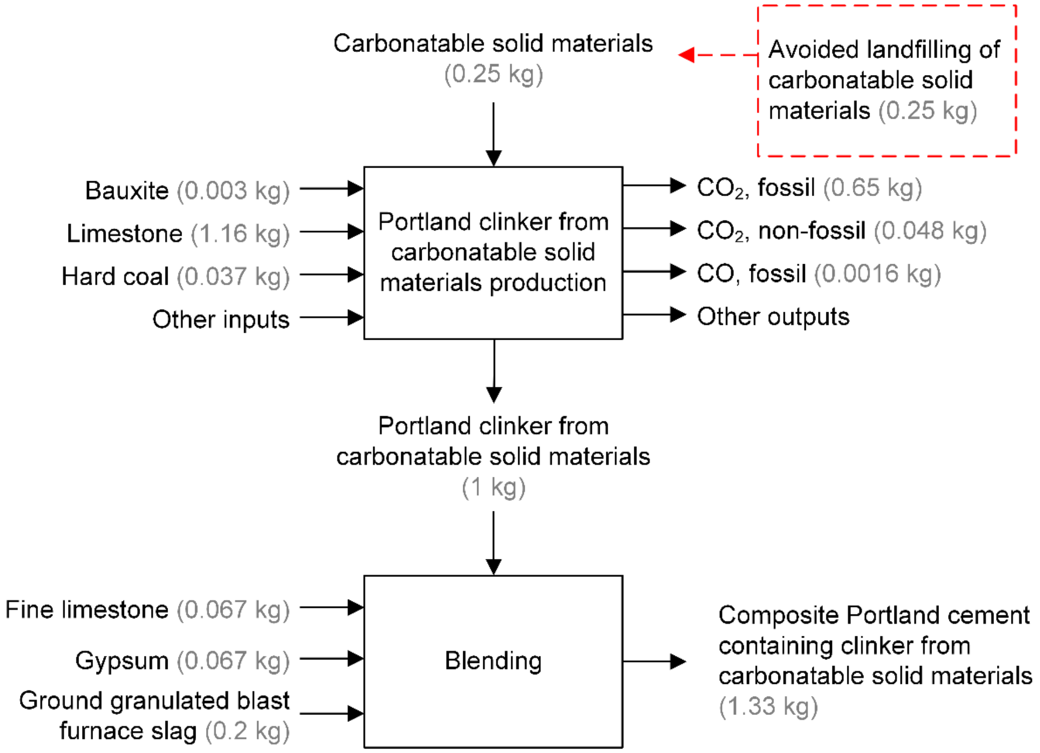

Fig. S6 | Flowchart of the production process for composite Portland cement containing clinker from carbonatable solid materials. ‘Other’ inputs and outputs to Portland clinker from carbonatable solid materials production are shown in full in Dataset S1, and are based on the ecoinvent v.3.8 unit process for clinker production (clinker production, CH; UUID be6ec015-2bd3-3199-9bbb-9555a2f7c046). Environmental credits modelled in this study are indicated by dashed red lines: We model carbonatable solid materials as burden free at their points of generation, and the use of carbonatable solid materials as avoided landfilling.

We derived a ‘blending’ unit process for composite PC containing clinker from carbonatable solid materials assuming a typical European composite cement composition (75 wt.% PC clinker, 15 wt.% ground granulated blast furnace slag, 5 wt.% fine limestone, 5 wt.% gypsum) (Fig. S6).

S4.1.3.2. Material properties

We assume that the material properties of composite PC containing clinker from carbonatable solid materials are similar to those for conventional composite PC, since the clinkers in both materials have the same target chemical composition and mineralogy. A key assumption here is

that the carbonatable solid materials contain low amounts of heavy metals and thus conform to international standards for clinker quality.

S4.2. Inert additives

S4.2.1. Carbonated recycled concrete aggregate

S4.2.1.1. Production process

Similarly to CCP, there are two types of processes to carbonate RCA: wet and dry [83-88]. Both carbonation processes affect the end-of-life cement paste but not the aggregates. Here, we model the wet process to carbonate RCA (Fig. S7). It is similar to the wet process to produce CCP but requires a larger carbonation reactor. The grinding to reduce particle size is already taken into account upstream during RCA production. Only the carbonation in a rather wet/humid environment and a slight drying step is considered here. We estimate that 0.96 kg of RCA, 0.25 kg of flue gas, and 0.13 kg of water are necessary to produce 1 kg of carbonated recycled concrete aggregate (CRCA).

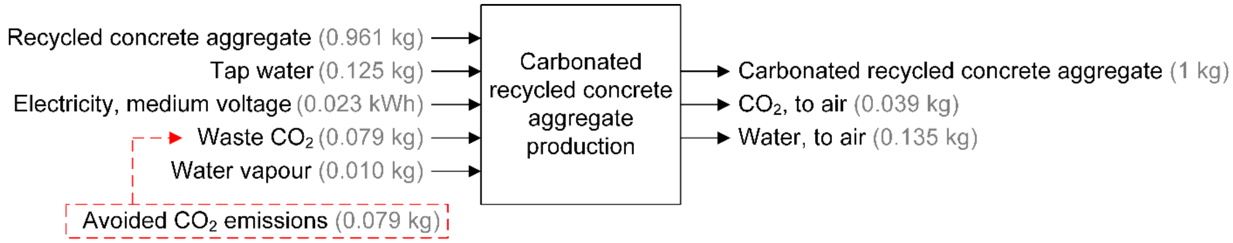

Fig. S7 | Flowchart of the (wet) production process for the carbonated recycled concrete aggregate. Environmental credits modelled in this study are indicated by red dashed lines: We model CO_2 in feedstock flue gas as waste, and all the CO_2 in feedstock flue gas that is absorbed by CO_2 mineralisation products as avoided CO_2.

Natural carbonation of concrete during use and at end-of-life limits the potential CO_2 uptake of recycled concrete aggregate. It has been reported that 10-20% of CO_2 emissions from concrete production are absorbed over its use phase, and an additional 5-35% are absorbed during the demolition/recycling phase, depending on the carbonation conditions and the exposure times [89,90].

S4.2.1.2. Material properties

After carbonation, CRCA contains natural aggregate and adhered CCP. Carbonation of RCA strengthens the adhered CCP and produces calcium carbonate, which increases the density of the material. The material properties of natural aggregate, RCA, and CRCA are shown in Table S4.

Table S4 | Reported material properties of natural, recycled concrete, and carbonated recycled concrete aggregate.

Properties	Recycled concrete aggregate compared to natural aggregate	Carbonated recycled concrete aggregate	Carbonated recycled concrete aggregate

		compared to recycled concrete aggregate	compared to natural aggregate
Crushing strength	Strongly decreased [91,92]	Increased [93,94]	Decreased [94]
Leaching resistance	Decreased [62]	In the same range [94]	Decreased [94]
Water adsorption	Strongly increased [62,94]	Decreased [93,94]	Increased [94]

The use of CRCA in concrete results in better mechanical properties than concrete containing (uncarbonated) RCA, but they remain slightly worse than concrete produced with natural aggregate (Tables S4-S5). In particular, carbonation of RCA reduces its water absorption, although it is still increased compared to concrete containing natural aggregate only (by up to 15%).

Table S5 | Reported properties of concrete containing natural, recycled concrete, and carbonated recycled concrete aggregate, adapted and extended from [62].

Properties	Recycled concrete aggregate compared to natural aggregate concrete	Carbonated recycled concrete aggregate compared to recycled concrete aggregate concrete	Carbonated recycled concrete aggregate compared to natural aggregate concrete
Compressive strength	Decreased up to 25 %[61,95-99]	Increased to 20 %[94,95,99,100]	Decreased up to 10 %[95,99,100]
Flexural tensile strength	Decreased up to 20 %[61,95,96,98]	Increased to 30 %[94,95,99,100]	Decreased up to 10 %[95,99,100]
Drying shrinkage	Increased up to 50 %[61,101,102]	In the range of the natural aggregate concrete[94]	Within range of natural aggregate concrete[94]
Creep	Increased up to 50 %[61,102]		
Water adsorption	Increased up to 50 %[101]	Decreased to 20 %[94,99]	Increased to 15 %[99]
Carbonation depth	≈[103,104]	-	-
Chloride penetration	≈ /or increased[104-106]	Decreased up to 35 %[94]	-

To conclude, CRCA has slightly improved material properties than (uncarbonated) RCA, but still at a poorer level than that for natural aggregate. However, regulations in some countries require or will require to use RCA as partial replacement of natural aggregates and carbonation generally improves its properties.

S4.2.2. Carbonated lightweight aggregate

S4.2.2.1. Production process

Carbonated (manufactured) lightweight aggregate (CLWA) is produced by carbonating instead of sintering pellets. We choose a generic production process based on several studies mainly using slags as the carbonatable solid material feedstock [107-112]. Its production process is shown in Fig. S8. It includes an initial soaking process (similar to that used for uncarbonated lightweight aggregate [LWA]), granulation (which is carried out in a drum under a stream of flue gas), the humidified CO_2 is then dried separately, and then the dry CO_2 stream is reintroduced into a curing chamber to carbonate the wet pellets [107,108]. We model CLWA as having comparable material properties to LWA in our LCA study.

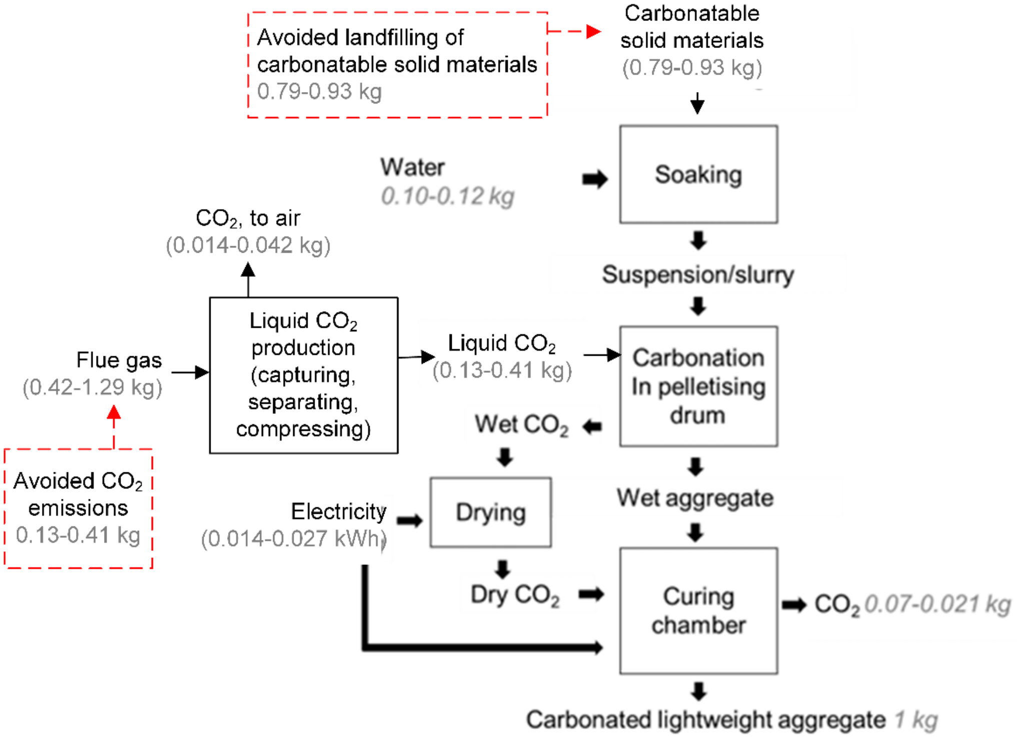

Fig. S8 | Flowchart of production processes for carbonated lightweight aggregate, adapted from [108] and [107]. CaO containing solids with 'low' (Fly Ash (Class F), conversion = 100%), 'typical' (CBD, conversion = 50%), and 'high' (CBD, conversion = 100%) CO_2 uptake potential were modelled in the LCA study to assess the effect of feedstock type on climate change impact. Environmental credits modelled in this study are indicated by red dashed lines: We model carbonatable solid materials as burden free at their points of generation, the use of carbonatable solid materials as avoided landfilling, CO_2 in feedstock flue gas as waste, and all the CO_2 in feedstock flue gas that is absorbed by CO_2 mineralisation products as avoided CO_2.

S4.2.2.2. Material properties

CLWA has lower strength and higher water absorption compared to natural aggregate, RCA, and CRCA. Typical values of compressive strength and water absorption are 3.0 MPa and 6.3%, respectively [112]. CLWA can valorise various industrial wastes/by-products that would otherwise be unutilised. However, the carbonation method is less adequate than sintering for the

encapsulation of heavy metals, chlorides, and sulphates that these materials often contain, so feedstock selection for CLWA needs to take this with respect to the intended application (e.g. in lightweight concrete blocks) into account. For feedstock materials with low concentrations of such impurities, CLWA can directly substitute conventional LWA.

S4.2.3. Carbonated normal weight aggregate

S4.2.3.1. Production process

Carbonated normal weight (manufactured) aggregate (CNWA) with similar density to natural aggregate can be produced using a similar process to that for CLWA. Overall, this process (Fig. S9) involves wetting the feedstock to ensure complete hydration, immediately followed by a shaping step (e.g. with a hydraulic press) that yields much higher densities relative to granulation. The formed (compressed) material is subsequently sent for carbonation [60,113]. Most studies in literature produce CNWA from industrial slags (e.g. basic oxygen furnace, electric arc furnace). Here, we model CNWA to have comparable material properties to normal weight aggregate (e.g. gravel) in our LCA study.

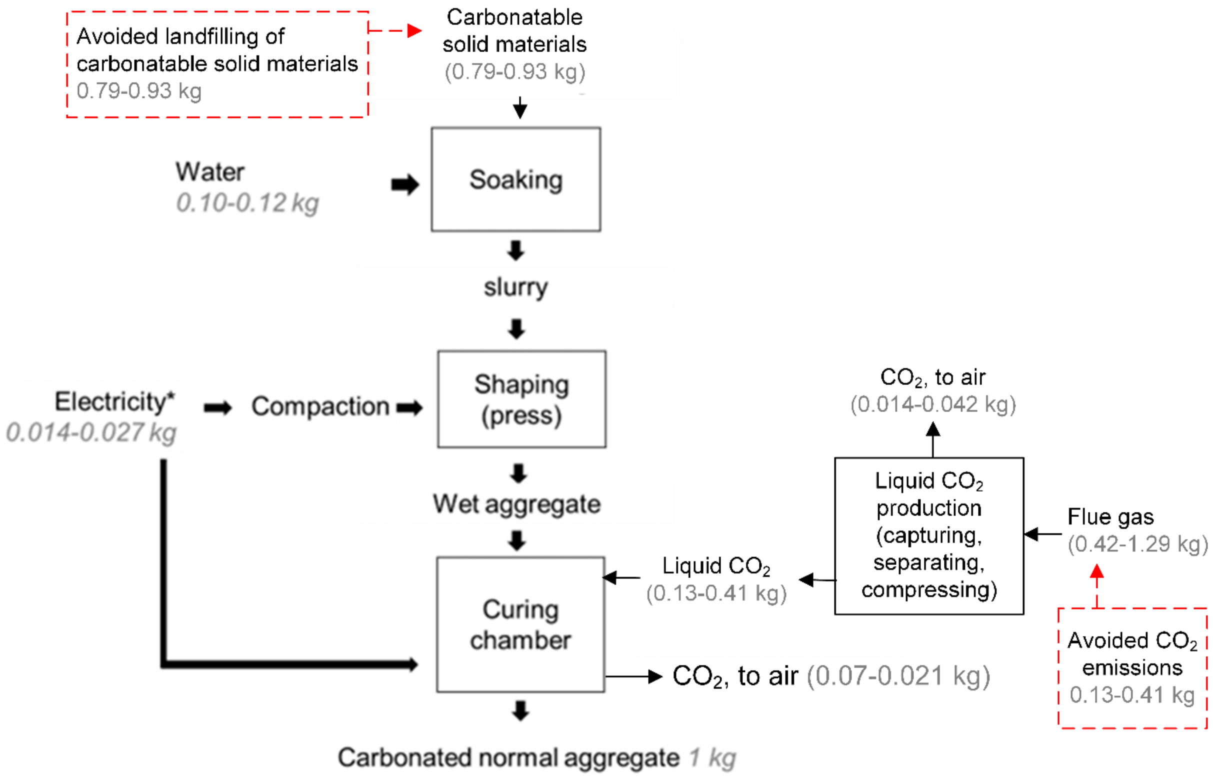

Fig. S9 | Flowchart of carbonated normal weight aggregate production (wet process). Environmental credits modelled in this study are indicated by red dashed lines: We model carbonatable solid materials as burden free at their points of generation, the use of carbonatable solid materials as avoided landfilling, CO_2 in feedstock flue gas as waste, and all the CO_2 in feedstock flue gas that is absorbed by CO_2 mineralisation products as avoided CO_2.

S4.2.3.2. Material properties

Most studies in this area have investigated the material properties of carbonated blocks (‘compacts’) instead of aggregates, specifically testing compressive strength, so we base our discussion here on those studies. Generally, CNWA is expected to have slightly higher water absorption than natural aggregates (Table S6). The compressive strengths and environmental properties (e.g. leaching of heavy metals) of CNWA is related to its density, which in turn depends on the compaction force [108,109]. Leaching is typically irrelevant for natural aggregates since they have insignificant heavy metal content, however this property is relevant for CNWA and CLWA derived from industrial by-products, which often have elevated heavy metal content. It has been shown that carbonation of industrial by-products at elevated temperatures (~100-140°C) can decrease leaching from the produced carbonated (manufactured) aggregates, although we do not consider this here.

Table S6 | Reported properties of carbonated normal weight aggregate derived from industrial by-products compared to natural aggregate (NA). BOFS = basic oxygen furnace slag, EAFS = electric arc furnace slag, SSS = stainless steel slag.

Carbonated normal weight aggregate	Material Properties				
	Initial Density (g/cm^3)	Water adsorption	Strength of blocks (MPa)	Leaching resistance	Freeze-thaw resistance
Based on BOFS [109]	1.9	-	10-12 (poorer than NA)	-	Poorer than NA
Based on BOFS [109]	2.2	-	35-45	-	Poorer than NA
Based on EAFS [114]	~2.4	-	40-50	-	-
Based on SSS[109]	1.9	Poorer than NA (6.9 wt%)	35-45	-	-
Based on SSS[109]	2.1	Poorer than NA (6.9 wt%)	50-60	-	-

In summary, CNWA can be produced with similar density and compressive strength to natural aggregates, but with somewhat poorer water absorption and freeze-thaw resistance, and with some risk of leaching of toxic substances (e.g. heavy metals).

S4.3. CO_2 curing

There are two predominant CO_2 curing approaches, both of which we analyse here. They are (i) CO_2 curing in presence of significant water, where binding relies on both hydrated and carbonated phases; or (ii) CO_2 curing with high CO_2 concentrations and insignificant water, where binding is mainly by carbonated phases. The former approach is more ubiquitous since it is relevant to conventional concrete, whereas the latter is usually applied on non-PC materials such as carbonatable calcium silicate cements.

S4.3.1. CO_2 curing of Portland cement concrete (ready-mix)

S4.3.1.1. Production process

CO_2 curing in ready-mix concrete involves injection of CO_2 into concrete trucks at batching plants, from a compressed CO_2 source. This process is shown in Fig. S10. This process includes production of conventional ready-mix concrete containing PC, ground granulated blast furnace slag, coal fly ash, sand, gravel, admixtures, and water, and additionally the capture and compression of CO_2 and its addition into fresh concrete. Here, we model ready-mix concrete with and without CO_2 curing as detailed in [115] to have comparable products in our LCA study.

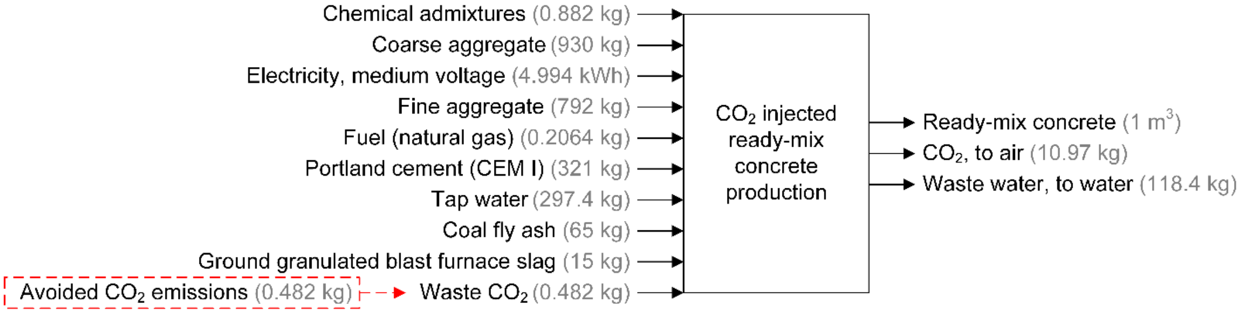

Fig. S10 | Flowchart of the production of CO_2 injected ready-mix concrete. The dataset is adapted from [115]. Environmental credits modelled in this study are indicated by red dashed lines: We model CO_2 in feedstock flue gas as waste, and all the CO_2 in feedstock flue gas that is absorbed by CO_2 mineralisation products as avoided CO_2.

S4.3.1.2. Material properties

CO_2 injected ready-mix concrete has similar material properties to comparable PC concrete (e.g. CEM I, CEM II; Table S7), although shows slightly accelerated setting[116,117]. However, in Europe, most of the cements available already contain significant quantities of limestone (>5 wt.%), which limits the potential of injected CO_2 to improve mechanical properties in the long term.

Table S7 | Summary of reported material properties of CO_2 injected ready-mix PC concrete compared to conventional hydrated ready-mix PC concrete. The changes in material properties shown are based on the cited reported data.

	Portland cement concrete	Portland cement/coal fly ash concrete	Portland cement/limestone concrete
Mechanical properties	Improved [118-120]	Improved [118,119]	decreased
Porosity	Improved [118,119]	Improved [118,119]	≈
Transport properties	Improved [118,119]	Improved [118,119]	No data
Durability	Improved [118,119]	Improved [118,119]	No data

S4.3.2. Carbonated Portland cement concrete products (unreinforced)

S4.3.2.1. Production process

Curing of PC concrete products can also be accelerated using CO_2 (‘forced carbonation’). This approach can be applied by exposing the concrete products to a high CO_2 atmosphere (e.g. in a carbonation chamber), with the depth of the carbonation depending notably on the porosity of the material (which is primarily a function of the water-to-binder ratio initially chosen) and humidity to which it is exposed. The relative humidity within these chambers should be relatively high (e.g. 70%) but not saturated (100%) to achieve fast carbonation rates. We model the process to carbonate concrete products (unreinforced) as shown in Fig. S11.

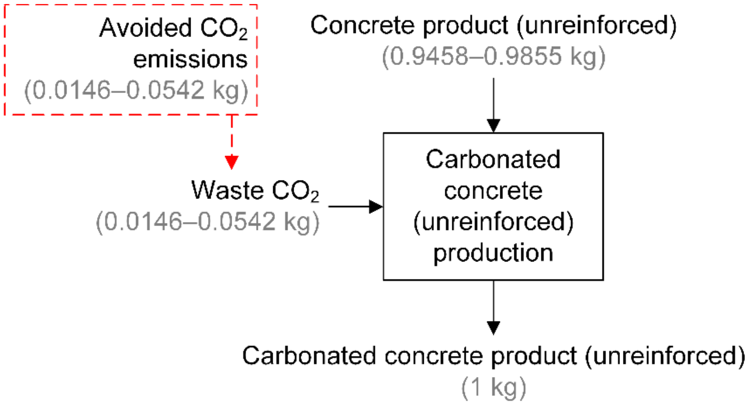

Fig. S11 | Flowchart for the production of carbonated concrete products (unreinforced). Environmental credits modelled in this study are indicated by red dashed lines: We model CO_2 in feedstock flue gas as waste, and all the CO_2 in feedstock flue gas that is absorbed by CO_2 mineralisation products as avoided CO_2.

Since the carbonation rate depends greatly on material porosity and the surface exposure condition (e.g. CO_2 concentration), we determine lower and upper boundaries for the carbonation extent of a typical concrete product, which is an unreinforced concrete block (0.4064 m length, 0.232 m width, 0.232 m depth):

1. Lower carbonation extent. This scenario models carbonation of 1 cm thickness on all sides of the concrete block, for a CO_2 uptake of 0.015 kg CO_2/kg carbonated concrete product. This scenario approximates carbonation at low (e.g. near ambient) CO_2 concentration with poor penetration of CO_2 into the concrete product; and
2. Higher carbonation extent. This scenario models carbonation of 80% of the concrete block, for a CO_2 uptake of 0.054 kg CO_2/kg carbonated concrete product. This scenario represents very high penetration of CO_2 into the concrete product, which would likely be challenging and require elevated CO_2 concentrations to achieve.

In this calculation we assume that the concrete product has a typical density of 2,300 kg/m^3 and cement content of 350 kg/m^3. Full details are provided in Dataset S1.

Since unreinforced concrete products do not contain steel reinforcement, there is no concern about pH reduction from carbonation, and theoretically carbonation can progress to completion (100% carbonated). Our high carbonation case is for 80% rather than 100% carbonation because we expect the hydration degree of typical concrete blocks and the practical design of the chambers to be somewhat limiting in this regard. We model carbonation of dense concrete

products here (Fig. S11, hence the large range of carbonation extents between 'lower' and 'higher' scenarios), since porous concrete products would naturally carbonate relatively quickly, typically within a few years, and are thus less relevant for CO_2 emissions reduction via CO_2 mineralisation.

S4.3.2.2. Material properties

Carbonation of conventional PC concrete products generally gives improved or similar material properties to the uncarbonated materials. For example, it has been shown that carbonation curing of a PC/FA system (30 wt.%) improves the mechanical properties after 2 days and gives similar mechanical properties after 28 days compared to PC concrete products for the same design [118,119]. Here, we model concrete products (unreinforced) with and without forced carbonation to have comparable material properties in our LCA study.

S4.3.3. Carbonatable calcium silicate cement (concrete products, unreinforced)

S4.3.3.1. Production process

The processes to produce carbonated concrete products and carbonatable calcium silicate concrete (CCSC) products are similar despite the cement compositions being different. We model the production of CCSC concrete using a typical mix design for unreinforced PC concrete blocks with a 1:1 mass replacement of CCSC for PC (i.e. cement : fine aggregate : coarse aggregate mass ratios of 1 : 1.3 : 4.5), consistent with its use in research trials and the comparable nature of these products[121]. Therefore, we model a 1 m^3 CCSC concrete block to contain 389 kg CCSC binder (including CCSC and CO_2 for curing), 405 kg fine aggregate, and 1,440 kg coarse aggregate (Fig. S12). A smaller amount of water (118 kg per m^3 CCSC concrete product here, which excludes mixing water) is used to produce CCSC concrete relative to PC concrete production since the main hardening reaction occurs via CO_2 curing. The modelled process includes the capture and compression of CO_2 for curing. We assume that CCSC production has a CO_2 footprint of 0.42 kg CO_2-eq. per kg CCSC binder, which is slightly lower than the reported estimate of 0.46 kg CO_2-eq. per kg CCSC binder for production in California (United States)[122].

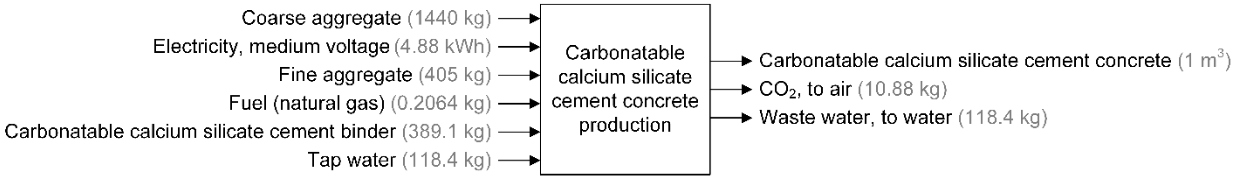

Fig. S12 | Flowchart for the production of carbonatable calcium silicate cement (CCSC) based concrete products (unreinforced). The CCSC binder shown is carbonated and has a CO_2 footprint of 0.42 kg CO_2-eq. per kg CCSC binder.

S4.3.3.2. Material properties

It takes ~1 day to produce (i.e. carbonate in a CO_2 chamber) concrete blocks based on CCSC[123,124]. Relatively little information is available in the literature regarding their mechanical properties, but they are claimed to have similar or enhanced compared to similar PC concrete

masonry units[125] and can be produced under ambient pressure [123,124]. Carbonation also improves other material properties compared to hydrated PC concrete, such as improved resistance to salt degradation [126]. Similarly to carbonated PC concrete, extensive forced carbonation (i.e. throughout the material) is incompatible with the use of reinforcing steel bars, since the pH of the carbonated cement binder is relatively low (pH < 10) and does not provide steel corrosion passivating conditions[124].

S4.3.4. Carbonate bonded compacts (concrete products, unreinforced)

S4.3.4.1. Production process

Carbonate bonded compacts can be produced similarly to carbonated normal weight (manufactured) aggregate (CNWA), by wetting, shaping, and carbonation (Section S4.2.3; Fig. S9). The main difference between these materials is product size. The size and porosity of the compacts influences the production rate since CO_2 transport is slower through less porous materials.[108] We use the same product system (Fig. S9) to model carbonate bonded compacts and CNWA. This means that our results are relevant for dense compacts (low porosity) and indicate the maximum utilisation of carbonatable solid materials and CO_2 uptake possible in carbonate bonded compacts.

S4.3.4.2. Material properties

Porous carbonate bonded compacts made from steel slags have been produced with sizes up to 1 m^3 and compressive strengths of 18 ± 3 MPa[108], and with densities of ~2 g cm^{-3} and compressive strengths of 10-134 MPa[109]. These values are comparable to or greater than the compressive strengths of conventional PC concrete products, and hence demonstrate their potential to achieve equivalent functional performance.

S5. Demand for CO_2 mineralisation products

S5.1. Market segments for concrete materials

Cement is mixed with fine aggregates (sand), coarse aggregates (e.g. gravel) and water to produce concrete, and with fine aggregates and water (without coarse aggregates) to make mortar. Mixture proportioning of cementitious products typically requires the adjustment of several mixture ingredients to achieve specified fresh and hardened properties. Table S8 shows the concrete and mortar mixture proportions adopted in our analysis to derive consumption levels of concrete materials globally. In the case of PC, a clinker-to-cement ratio of 0.75 is assumed, based on current European practice[127].

Table S8 | Cement, mortar, and concrete mix designs used in this study.

%	Clinker	Cement	Lime	Sand	Coarse aggregates	Total aggregates
Cement	75%					
Mortar	9.0%	12%	4%	73%		73%
Concrete	11.3%	15%		30%	46%	76%

Concrete can be produced in three main ways:

(1) Ready-mix, where the wet mixture is poured 'in-situ' from concrete trucks;
(2) Concrete products, or precast, where concrete products are made in a factory then assembled on-site; or
(3) From bagged cement, which is then mixed and placed at a small scale 'in-situ', including in artisanal uses.

As Table S9 shows, the highest single market for cement globally is bagged cement (which is eventually applied in mortar and concrete), due to the relatively low levels of industrialised construction in developing countries. Significant amounts of cement are used in mortar and concrete, and due to the relatively small masses of cement in these materials, their production volumes are substantially larger than for cement. We estimate that 16.7-21.7 Gt of concrete was produced in 2020 by assuming 60% (lower bound) and 100% (upper bound) of bagged cement is used to make concrete at a concentration of 15 wt.% (and adding this mass of concrete to that for ready-mix concrete and concrete products). We also estimate that 7.62 Gt mortar was produced in 2020 globally, which represents a significant end-use of cement.

Table S9 | Summary of global market segments for concrete materials in 2020. SCMs = supplementary cementitious materials.

Concrete material	Global demand (Gt in 2020)
Aggregates (total)	46.4
…as normal weight aggregate (coarse)	29.1
…as normal weight aggregate (fine)	10.5
…as recycled concrete aggregate	4.93
…as lightweight aggregate	1.02
Ready-mix concrete	6.09
Concrete products	3.05
Mortar	7.62

Cement (total)	4.16
…as bagged cement	1.88
…in mortar	0.91
…in ready-mix concrete	0.91
…in precast concrete	0.46
SCMs excluding limestone	1.04
Fine limestone	0.21

In 2019, around 140 Mt of Portland clinker was used to make up the 182 Mt of cement consumed in Europe[128]. Of all the cement sold in Europe, 47% contains fine limestone[129]. CEM II represents ~50% of the market and ~50% of the CEM II are CEM II/A-L, CEM II/A-LL, CEM II/B-L, and CEM II/B-LL [130], with ~15 wt.% replacement of Portland clinker by in CEM II/A and ~25 wt.% in CEM II/B.

S6. Climate change impacts of CO_2 mineralisation products

S6.1. Goal and scope

We conducted a life cycle assessment (LCA) study to estimate the potential of CO_2 mineralisation products to reduce greenhouse gas (GHG) emissions. We investigated a range of CO_2 mineralisation products that have varying functionalities, technological readiness, market segments, and material properties (Table S10). In addition, some of these products have not been studied by LCA previously. Therefore, the goal of our LCA study is to establish the first reliable quantitative comparison of the climate change impacts of a wide range of CO_2 mineralisation products. To achieve this, we analyse substitutions of conventional products for CO_2 mineralisation products that are at least functionally equivalent, meaning that the CO_2 mineralisation product has comparable key material property values (e.g. 20 MPa 2 days compressive strength) or the CO_2 mineralisation product performs better. For example, one substitution analysed in our LCA study is carbonated concrete products for conventional concrete products (unreinforced). In Table S10 we show qualitatively that the substitutions analysed maintain this condition of functional equivalence.

Accordingly, our LCA study uses 1 kg material as the functional unit for each CO_2 mineralisation-conventional product pair, including for reactive additives and inert additives (e.g. 1 kg material for precipitated calcium carbonate and fine limestone), and for CO_2 curing (including ready-mix concrete and concrete products). This functional unit is appropriate for the modelled ready-mix concrete products here since the masses of 1 m^3 ready-mix concrete with (2,303 kg) and without (2,306 kg) CO_2 injection are similar. We also consider this functional unit to be appropriate for concrete products here, since we focus on products with similar high density (e.g. 'precast' slabs or dense concrete masonry units) rather than cement type; our LCA study aims to provide representative results for product types rather than for specific products. Overall, our LCA study tends towards providing lower estimates for greenhouse gas emissions reductions since CO_2 mineralisation products with both better and comparable performance are treated to be functionally equivalent to conventional products.

Table S10 | Summary of CO_2 mineralisation products considered here. Their key functions and technical properties are listed; the latter obtained through literature review.

CCU technology	Function	Substitutes for (conventional technology)	Key material properties	Material properties relative to comparable conventional technology	Carbonatable raw materials required[a]
**Reactive additives**	-		-	-	-
Precipitated calcium carbonate	Partial cement substitute	Fine limestone	Reactivity and 2 and 28 days compressive strength of the concrete produced	Equivalent, potential higher reactivity ≈	Industrial by-products (Ca-rich, uncarbonated)
Cement from carbonated end-of-life cement paste	Partial cement substitute	Fine limestone and pozzolanic secondary materials (e.g. coal fly ash) and/or PC clinker	Reactivity and 2 and 28 days compressive strength of the concrete produced	Equivalent, potential higher reactivity of the $CaCO_3$, potential lower reactivity of the amorphous (alumino)-silicate ≈	End-of-life concrete, end-of-life mortar
**Inert additives**	-		-	-	-
Carbonated recycled concrete aggregate	Aggregate substitute (normal)	Normal (recycled) aggregate (coarse/fine)	Crushing strength, water uptake, porosity (etc.)	Higher crushing strength<br>Lower water uptake<br>Higher density	End-of-life concrete, end-of-life aggregates
Carbonated normal weight aggregate	Aggregate substitute (normal)	Normal (virgin) aggregate (coarse/fine)	Crushing strength, water uptake, density (etc.)	Lower crushing strength<br>Higher water uptake<br>Lower density	End-of-life concrete, end-of-life aggregates / Industrial by-products (Ca-rich, uncarbonated)
Carbonated lightweight aggregate	Aggregate substitute (lightweight)	Lightweight aggregate (coarse/fine)	Crushing strength	Similar or higher crushing strength	Virgin porous rocks, industrial by-products (Ca-rich, uncarbonated)
**CO_2 curing**	-		-	-	-
CO_2 injection (ready-mix Portland-based concrete)	Concrete curing (strength accelerator)	Fine limestone (ready-mix concrete)	2 and 28 days compressive strength	Similar properties	Ready-mix concrete
Carbonated calcium silicate cement concrete	Concrete curing	Hydration (concrete products)	2 and 28 days compressive strength	Similar properties excepted low pH values of the matrix, cannot be reinforced	Carbonated calcium silicate cement
Forced carbonation (concrete products, unreinforced)	Concrete curing	Hydration (concrete products, unreinforced)	28 days compressive strength	Similar properties excepted low pH values of the matrix, cannot be reinforced	Concrete products (unreinforced)

[a] CO_2-containing flue gas is additionally required for all the CO_2 mineralisation products

The scope of our LCA study starts at the point of generation (extraction from the ground, demolition at a construction site, etc.) of raw materials through to material produced at the product manufacturer gate, including curing (by water or CO_2) and transport where relevant, and generally represents production of cementitious materials in a generic European location in ca. 2020. For recycling of end-of-life carbonatable solid materials, we assume that they are burden free at their points of generation, and transportation from those points of generation to CO_2 mineralisation product factory is 300 km by lorry (>32 t capacity, type EURO5). For recycling of CO_2, we assume that transportation is negligible in all cases except for CO_2 injection into ready-mix concrete, which is consistent with sourcing CO_2 for carbonation processes from cement plant flue gas and carbonation processes occurring at cement plants. Due to the different scales of production (pilot/laboratory-to-industrial vs. industrial) of CO_2 mineralisation and conventional products in 2020, and the global scale that we use to model CO_2 mineralisation product-for-conventional product substitutions, our LCA study results provide theoretical potentials of CO_2 mineralisation products to reduce CO_2 emissions.

By-product inputs (including end-of-life materials) to the modelled CO_2 mineralisation processes were treated as avoided waste treatment (e.g. end-of-life concrete inputted into the cement from CCP system avoids inert waste landfilling) or emissions (e.g. CO_2-containing flue gas feedstock avoids CO_2 emissions), which are thus environmental credits. By-products output by the modelled product systems were modelled as avoided production of comparable products (e.g. secondary coarse aggregate produced by the cement from CCP system avoids production of primary gravel aggregate), which also represent environmental credits. The specific application of this allocation approach is described in each dataset (see Dataset S1).

We used datasets from ecoinvent v.3.8 (cut-off system model) or from the literature (e.g. [131]) to describe conventional processes if available, or otherwise developed our own datasets (using assumptions based upon our review of the literature, domain expertise, and process modelling). We use of the cut-off rather than the at-point-of-substitution system model of ecoinvent since it is simpler and consistent with the inventory data for supplementary cementitious materials that include treatment/recycling processes such as grinding in their scopes[131]. We note that the impacts of these recycling processes are usually small compared to the reductions in greenhouse gas emissions achieved by reducing Portland clinker content and valorisation of waste CO_2[16].

The following ecoinvent v3.8 datasets were used:

- Gypsum: “market for gypsum, mineral”, Europe (UUID 9e43a01c-7761-3e43-90d3-7c649c98d5da); producing gypsum, mineral (UUID dd0cbafd-b176-4ba4-860a-25a2e0663d14)
- PC clinker: “market for clinker”, Switzerland (UUID 218ea4b1-9fa2-38e6-a677-19adec63da07); producing clinker (UUID 1f41586d-0d8a-4c7c-8473-dd8351bab538)
- Portland cement (CEM I): “market for cement”, Switzerland (UUID 3fc1e4d3-d345-380a-978e-b1df2f7c4784); producing cement (UUID c5299a8e-d4b4-409c-9826-e8ae9e37c03d)
- Coarse (virgin) aggregate: “market for gravel, round”, Switzerland (UUID 7db49166-eb8a-34fe-acd1-2c23b7ddbb24); producing gravel, round (UUID fd3ee522-16db-41e4-967f-8bffe0b0ea87)

- Fine (virgin) aggregate: “market for sand”, Switzerland (UUID 0cffdb3d-84b3-34e1-b602-27780aa733c5); producing sand (UUID f51d7ccf-0bee-430d-98a3-8334adbe39fc)
- Tap water: “market group for tap water”, Switzerland (46034b1a-caf9-3228-aa1f-94aa4ed08f59); producing tap water (UUID c5adb1fb-872e-4446-a3bb-c4b61aa4bd45)
- Deionised water: “market for water, deionised”, Switzerland (UUID 6d1236af-2405-3bf3-a0e9-39d90edcc85a); producing water, deionised (UUID 360e2eb0-f81c-4e4b-ba6b-c7a690f31275)
- Superplasticiser: “market for plasticiser, for concrete, based on sulfonated melamine formaldehyde”, global (UUID 4bdecff2-b935-3b42-abda-1fca2ef70a93); producing plasticiser, for concrete, based on sulfonated melamine formaldehyde (UUID 46cb9cd6-9a86-4f77-bec3-3f946ed5e32f)
- Ammonium chloride: “market for ammonium chloride”, global (UUID 124cddb1-6b83-3d0b-b2cc-60f2b3baf08f); producing ammonium chloride (UUID e03dc076-23ea-4705-b9f5-7bba07abd0ae)
- Concrete blocks: “market for concrete block”, Germany (UUID 0572c6af-b8cb-39da-a063-4c8fffaab390); producing concrete block (UUID d4ee8f39-342b-4443-bbb9-c49b6801b5d6)
- Diesel: “market for diesel”, Switzerland (UUID 7615f5f8-2d72-33fa-98f7-dbac8699cfb1); producing diesel (UUID 291fc06d-1b3e-4077-aabb-346b588ed24b)
- Natural gas: “market for natural gas, high pressure, vehicle grade”, global (UUID 0232e332-064e-3039-af58-d14f6a6e19f7); producing natural gas, high pressure, vehicle grade (UUID f56d1311-8d94-40ca-a6ed-86dfb2dc63ea)
- Electricity: “market for electricity, medium voltage”, Switzerland (UUID 5a9a340e-3ab7-350e-a317-27fc04dd141a); producing electricity, medium voltage (UUID 759b89bd-3aa6-42ad-b767-5bb9ef5d331d)
- Transport: “market for transport, freight, lorry >32 metric ton, EURO5”, Europe (UUID a2be042d-d638-300b-a34d-5fab36b7dcc3); producing transport, freight, lorry >32 metric ton, EURO5 (UUID 93afc03f-5832-4ec0-8d76-cf6e62efff26)
- Lightweight aggregate: “market for expanded clay”, global (UUID 6ff0b772-9b13-37e6-b94b-90a88686e8b2); producing expanded clay (UUID 6968a565-8132-49c4-b3ff-1d0865af0fdc)
- Landfilling of inert waste: “treatment of inert waste, inert material landfill” Switzerland (UUID 336a605e-22fc-3a55-812e-aa01baf28fc2); treating inert waste, for final disposal (UUID 240c1a3c-1aba-4528-afc3-3f27f56583be)
- Steel reinforcing bar: “reinforcing steel production”, Austria (UUID be906a8d-7adb-30db-bb01-4a6a3eea9bf3); producing reinforcing steel (UUID 9ba48284-0f03-4fec-800d-de77833b12f6)

Unit process datasets were developed for the processes described in Section S4. Notably, data from the literature were used to model:

- Conventionally cured and CO_2 injected ready-mix concrete, from Monkman and MacDonald (2017)[117]
- Recycled concrete aggregate, from Marinkovic et al. (2010)[62]
- Precipitated calcium carbonate, from Hargis et al. (2021)[67]
- Cement from carbonated cement paste, from Zajac et al. (2020)[77]

- Fine limestone, ground granulated blast furnace slag, and coal fly ash from Miller et al. (2018)[131]

The properties of the datasets used for each LCA product system modelled here are summarised in Table S11 using pedigree matrix[132] factors. Although the datasets vary in terms of their temporal and geographical scopes, the technology-related uncertainty is low. This is important since technology-related uncertainty, specifically the Portland clinker content in the concrete materials studied here, is the factor that has the greatest effect on total uncertainty and the quantitative LCA results.

Table S11 | Properties of the inventory analysis datasets used in the LCA study reported using pedigree matrix factors.

Product	Reliability	Completeness	Temporal scope	Geographical scope	Technological scope
Reactive additives					
Conventional					
Fine limestone	Peer-reviewed data[131]	Representative, generic	ca. 2012	Global	Same process
Ground granulated blast furnace slag	Peer-reviewed data [131]	Representative, generic	ca. 2012	Global	Same process
Coal fly ash	Peer-reviewed data [131]	Representative, generic	ca. 2012	Global	Same process
Portland cement clinker	Ecoinvent verified data	Representative, industry association data	2005-2009	Europe	Same process
Gypsum	Ecoinvent verified data	Representative, reported data	2003-2021	Europe	Same process
Composite Portland cement (clinker-to-cement ratio = 0.75)	Verified data partly based on assumptions	Representative, generic	2005-2009	Europe, global	Same process
CO_2 mineralisation products					
Precipitated calcium carbonate	Verified data based on measurements [67]	Representative, measured data from industry (pilot scale)	ca. 2021	Europe	Same process
Composite Portland cement with precipitated calcium carbonate (clinker-to-cement ratio = 0.75)	Verified data partly based on assumptions	Representative, generic	ca. 2021	Europe	Same process
Carbonated cement paste (excl. carbonation)	Verified data partly based on assumptions	Representative, generic	2021	Europe	Same process

Carbonated cement paste (incl. carbonation)	Verified data partly based on assumptions	Representative, generic	2021	Europe	Same process
Cement from carbonated cement paste (clinker-to-cement ratio = 0.57)	Verified data partly based on assumptions	Representative, generic	2021	Europe	Same process
Cement from carbonated cement paste (clinker-to-cement ratio = 0.57)	Verified data partly based on assumptions	Representative, generic	2021	Europe	Same process
Portland cement clinker produced with carbonatable solid waste	Verified data partly based on assumptions	Representative, generic	2005-2009	Europe	Same process
Composite Portland cement (from Portland cement clinker produced with carbonatable solid waste) (clinker-to-cement ratio = 0.75)	Verified data partly based on assumptions	Representative, generic	2005-2009	Europe	Same process
Inert additives					
Conventional					
Recycled concrete aggregate	Peer-reviewed data [62]	Representative, data from industry	ca. 2010	Europe	Same process
Normal (virgin) aggregate	Ecoinvent verified data	Representative, measured data from four aggregate plants	1997-2020	Switzerland	Same process
Lightweight aggregate	Ecoinvent verified data	Representative, generic	2000-2021	Germany	Same process
CO_2 mineralisation products					

Carbonated (recycled concrete) aggregate	Verified data partly based on assumptions	Representative, generic	ca. 2010	Europe	Same process
Carbonated (normal) aggregate (low)	Non-verified data partly based on qualified estimates	Representative, generic	2017-2021	Europe, USA	Same process
Carbonated (normal) aggregate (typical)	Non-verified data partly based on qualified estimates	Representative, generic	2017-2021	Europe, USA	Same process
Carbonated (normal) aggregate (high)	Non-verified data partly based on qualified estimates	Representative, generic	2017-2021	Europe, USA	Same process
Carbonated (lightweight) aggregate (low)	Non-verified data partly based on qualified estimates	Representative, generic	2017-2021	Europe, USA	Same process
Carbonated (lightweight) aggregate (typical)	Non-verified data partly based on qualified estimates	Representative, generic	2017-2021	Europe, USA	Same process
Carbonated (lightweight) aggregate (high)	Non-verified data partly based on qualified estimates	Representative, generic	2017-2021	Europe, USA	Same process
CO_2 curing					
Conventional					
Hydrated PC concrete (ready-mix, 20% fly ash and slag cement)	Peer-reviewed data [117]	Representative, generic	2005-2009	Europe, USA	Same process
Hydrated PC concrete (concrete products, unreinforced)	Ecoinvent verified data	Representative, generic	2005-2009	Germany	Same process
CO_2 mineralisation products					

CCSC binder	Industry quantified data consistent with peer-reviewed data from [122]	Representative, data from industrial producer	ca. 2022	USA	Same process
CO_2 injected PC concrete (ready-mix, 20% fly ash and slag cement)	Peer-reviewed data [117]	Representative, data from industrial producer	ca. 2017	Europe, USA	Same process
Carbonated PC concrete (concrete products, unreinforced) (low)	Verified data partly based on assumptions	Representative, generic	2005-2009	Germany	Same process
Carbonated PC concrete (concrete products, unreinforced) (high)	Verified data partly based on assumptions	Representative, generic	2005-2009	Germany	Same process
CCSC concrete (concrete products, unreinforced)	Industry quantified data consistent with peer-reviewed data from [122]	Representative, data from industrial producer	ca. 2022	Europe, USA	Same process
Carbonate bonded compacts (concrete products, unreinforced) (low)	Non-verified data partly based on qualified estimates	Representative, generic	2017-2021	Europe, USA	Same process
Carbonate bonded compacts (concrete products, unreinforced) (typical)	Non-verified data partly based on qualified estimates	Representative, generic	2017-2021	Europe, USA	Same process
Carbonate bonded compacts (concrete products, unreinforced) (high)	Non-verified data partly based on qualified estimates	Representative, generic	2017-2021	Europe, USA	Same process

Other processes					
Treatment of inert waste, for final disposal	Ecoinvent verified data	Representative, from an industry survey of construction and demolition waste	1995-2021	Switzerland	Same process
Uncarbonated coarse aggregate	Ecoinvent verified data	Representative, measured data from four aggregate plants	1997-2020	Switzerland	Same process

We used the IPCC 2013 impact assessment methodology to determine climate change impacts for conventional and CCU products in 100-year global warming potential (greenhouse gas emissions in kg CO_2-eq.). LCA modelling was performed in OpenLCA v.1.11.0. Potential changes in greenhouse gas emissions resulting from substituting conventional products with CO_2 mineralisation products were calculated at the product level, i.e. on the functional unit basis of 1 kg material, using Eq.(S1):

$$\Delta i_j = \left(i_{j,\mathrm{CO_2\ mineralisation}} - i_{j,\mathrm{conventional}} \right) \tag{S1}$$

Where i_j is the environmental impact indicator value (i, greenhouse gas emissions in kg CO_2-eq.) per functional unit (e.g. 1 kg product) for a product of type j, the symbol Δ refers to a change in the associated parameter (in this case i_j), and the subscripts 'CO_2 mineralisation' and 'conventional' refer to the technology class. Therefore, a reduction in environmental impact is calculated for substitutions within a given product type (e.g. coarse normal weight aggregate) if the CO_2 mineralisation product has a smaller environmental impact than the conventional product per 1 kg product (the functional unit).

Product level environmental impact indicator values ('typical' or average values were used for upscaling when sensitivity analyses were conducted) were upscaled to the market level (i.e. global) using either generation rates for carbonatable solid materials (e.g. construction and demolition waste concrete) or market sizes for product types (e.g. ready-mix concrete) in ca. 2020, i.e. following Eq.(S2):

$$\Delta I_j = M_j \left(\Delta i_j \right) \tag{S2}$$

Where M_j is the generation rate of carbonatable solid materials or the market size for product type j. A similar calculation method (linear upscaling of environmental impact indicator values) was used by Miller et al. (2018)[131] in their assessment of CO_2 emissions reduction potentials for various cement decarbonisation measures.

S6.2. Sensitivity analysis

We performed sensitivity analyses for various CO_2 mineralisation technology datasets (production of cement from CCP, CNWA, CLWA, and carbonated concrete products (unreinforced)) to improve reliability of the LCA results and to accommodate the wide range of carbonatable solid materials considered in our study:

- For cement from CCP production, we calculated lower and upper values of CO_2 uptake by considering co-production of either uncarbonated (lower CO_2 uptake) or carbonated (higher CO_2 uptake) coarse and fine recycled aggregate.
- We modelled three scenarios for 'low', 'typical', and 'high' CO_2 uptake in the production of both CNWA and CLWA, to cover the range of carbonatable solid materials considered, which vary significantly in CaO content. The 'low', 'typical', and 'high' CO_2 uptake scenarios were respectively based on 100% carbonation of Class F coal fly ash (a relatively low CaO material), 50% carbonation of cement bypass dust (a relatively high CaO material), and 100% carbonation of cement bypass dust.

- Two scenarios were modelled for the uptake of CO_2 in the production of carbonated unreinforced concrete products, to cover a range of carbonation conditions (e.g. slight vs. extreme forced carbonation). These scenarios range from relatively superficial surface carbonation through to extensive carbonation throughout most of the product mass.

S6.3. Results and discussion

The product level LCA results (Fig. 1, main text) show that the substitution of CLWA for conventional lightweight aggregate (expanded clay) has high greenhouse gas emissions reduction potential (–0.46 to –0.57 [typically –0.49] kg CO_2-eq. emissions per kg lightweight aggregate) at this scale. It is a significantly greater emissions reduction potential than that calculated for the substitution of CNWA (carbonation of compacted carbonatable solid materials) for conventional normal weight aggregate (gravel, round) (–0.06 to –0.17 [typically –0.10] kg CO_2-eq. emissions per kg normal weight aggregate). This result arises from the much higher amount of greenhouse gas emissions that are generated from expanded clay production (0.43 kg CO_2-eq. emissions per kg lightweight aggregate) relative to gravel (round) production (0.0051 kg CO_2-eq. emissions per kg gravel (round)), despite the two processes being similar (granulation followed by carbonation vs. compaction followed by carbonation). Therefore, the result clearly shows a need to develop and use lower greenhouse gas emissive processes than conventional expanded clay production.

The LCA results show significant potential reductions in greenhouse gas emissions for all the reactive additive type CO_2 mineralisation technologies considered here. Substitution of PCC (from carbonatable solid materials) for fine limestone was calculated to have a greater greenhouse gas emissions reduction potential at the product level (–0.28 kg CO_2-eq. emissions per kg fine limestone product) than substitution of cement from CCP for conventional composite PC (75 mass% clinker) (–0.19 to –0.21 [mean –0.20] kg CO_2-eq. emissions per kg cement). This result is consistent with the nature of the two CO_2 mineralisation products: PCC is a completely carbonated material whereas cement from CCP is modelled to contain 38 mass% CCP, 57 mass% PC clinker, and 5 mass% gypsum (consistent with the start-of-the-art mix design[77]). The key insight from these results is that CO_2 mineralisation products have a greater potential to reduce greenhouse gas emissions when they are used to substitute more highly emissive conventional technologies.

In contrast to the product level LCA results for inert and reactive additives, relatively low reductions in greenhouse gas emissions were calculated for the CO_2 curing technologies. This result is consistent with the generally much lower relative content of CaO and thus CO_2 uptake in these CO_2 curing (concrete) products than in the inert and reactive additives. They show the relatively high potential for forced carbonation of conventional PC based concrete products to reduce greenhouse gas emissions, which is often overlooked in the literature relative to utilisation of alternative cement chemistries (such as CCSC), demonstrating a need to develop and apply technologies in this area (to achieve the extensive carbonation extents modelled, e.g. for carbonated unreinforced concrete products, resulting in a maximum greenhouse gas emissions reduction potential of –0.02 to –0.06 [mean –0.04] kg CO_2-eq. emissions per kg product). Given the vast excess of waste CO_2 that is available and the already proven commercial viability of CO_2 injection into ready-mix concrete, another key insight from this LCA study is that CO_2 injected ready-mix concrete should generally always be used unless there are specific

reasons not to do so (since the application of this technology results in a reduction in greenhouse gas emissions).

We upscaled our product level results to the market level using a scenario that maximises the total amount of greenhouse gas emissions that can potentially be avoided through CO_2 mineralisation for conventional product substitutions. This scenario prioritises such substitutions in product types that are currently cost-competitive, and then those that have the highest potential to reduce greenhouse gas emissions (Fig. S13), to maximise utilisation of carbonatable solid materials and fulfil demand in the most important market segments for decarbonisation (cement, etc.). We separately considered utilisation of end-of-life concrete and other carbonatable solid materials due to the unique potential of end-of-life concrete to be used as a feedstock for cement from CCP. Therefore, results calculated using Eq.(S2) and this scenario represent the maximum potential of CO_2 mineralisation technologies to reduce the greenhouse gas emissions at the global scale (ca. 2020). These results are shown in Fig. 3 in the main text.

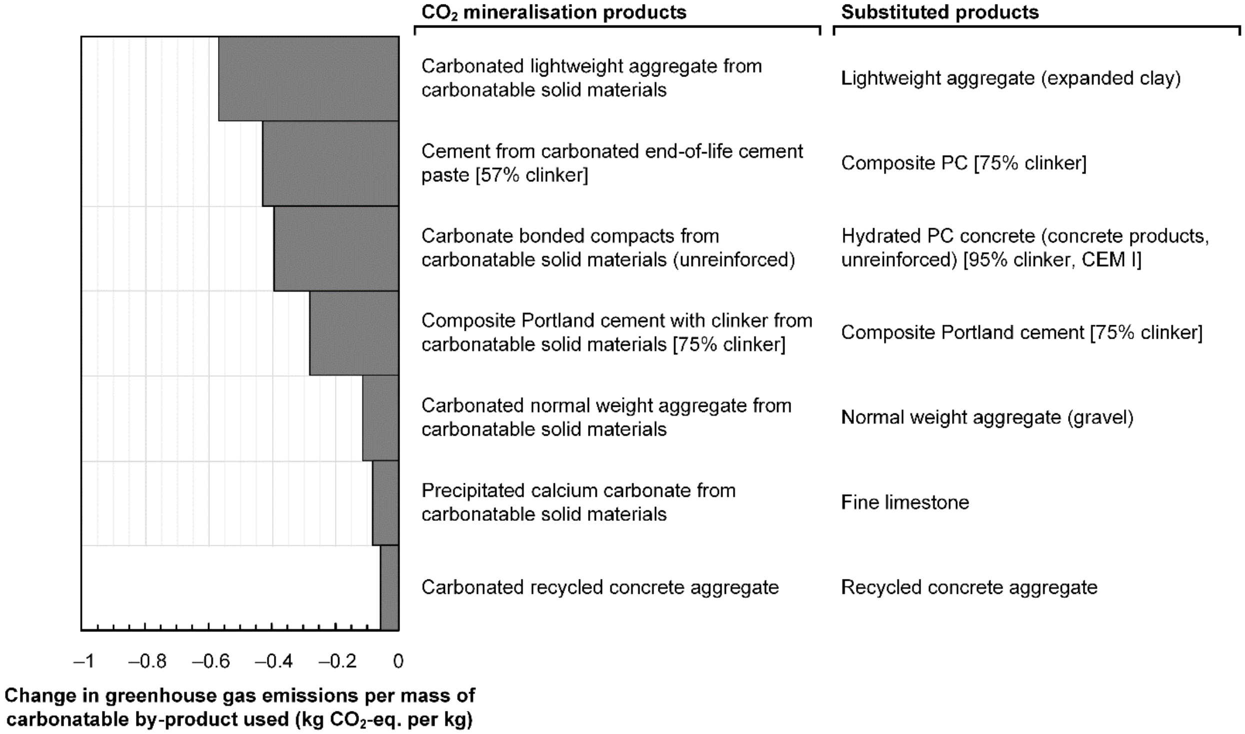

Fig. S13 | Greenhouse gas emissions reduction efficiency per mass of carbonatable solid material for the CO_2 mineralisation technology substitutions considered here. CO_2 mineralisation technology substitutions towards the top of this figure are more efficient i.e. reduce greenhouse gas emissions by more per mass of carbonatable solid material used. Composite Portland cement with clinker from carbonatable solid materials is not a CO_2 mineralisation technology but is included because it competes for the same resources i.e. carbonatable solid materials.

The scenario was modelled using the following procedure:

1. Use (1.06 Gt) of end-of-life cement paste to produce (1.99 Gt) cement from CCP. This approximates complete use of the supply of end-of-life cement paste and results in the avoidance of 0.39 Gt CO_2-eq. emissions.
2. Use (0.89 Gt) carbonatable solid materials excluding end-of-life concrete (e.g. coal fly ash) to produce (1.02 Gt) CLWA. This fulfils the global demand for lightweight aggregate (1.02 Gt in 2020) and results in the avoidance of 0.50 Gt CO_2-eq. emissions.
3. Use (0.74 Gt) carbonatable solid materials excluding end-of-life concrete to produce (2.2 Gt) composite PC containing clinker from carbonatable solid materials. This fulfils the global demand for cement (4.16 Gt in 2020) and results in the avoidance of 0.21 Gt CO_2-eq. emissions.
4. Use (0.91 Gt) carbonatable solid materials excluding end-of-life concrete to produce (1.03 Gt) carbonate bonded compacts (concrete products, unreinforced). This approximates complete use of all the carbonatable solid materials analysed here and results in the avoidance of 0.23 Gt CO_2-eq. emissions.
5. Use waste CO_2 to produce (2.02 Gt) carbonated calcium silicate concrete products (unreinforced) and (6.09 Gt) CO_2-injected ready-mix concrete. This step fulfils the global demand for concrete products and ready-mix concrete (waste CO_2 is available in excess) and results in the avoidance of 0.09 and 0.03 Gt CO_2-eq. emissions, respectively.

Our application of this scenario resulted in an overall potential reduction in greenhouse gas emissions of 1.46 Gt CO_2-eq. emissions. This value represents 33% of total CO_2-eq. emissions from production of non-metallic minerals (4.4 Gt CO_2-eq. in 2015)[133] or 57% of CO_2-eq. emissions from cement production (~2.6 Gt CO_2-eq. in 2020)[16], demonstrating the huge potential for CO_2 mineralisation as a climate change mitigation strategy. The results also highlight the significant opportunities that are available for organisations within the concrete value chain to implement CO_2 mineralisation to reduce greenhouse gas emissions, including application of the technologies in industrial plants as well as improved collection of carbonatable solid materials (which are generated in various locations, from construction sites through to industrial plants).

S7. Economics of CO_2 mineralisation products

We evaluated the economic performance of CO_2 mineralisation products (and composite PC with clinker from carbonatable solid materials) against comparable conventional products, combining them with our product level environmental life cycle assessment results (Fig. 1, main text). We also performed this analysis for calcium looping and amine scrubbing CCS, since CO_2 storage is the direct alternative to CO_2 utilisation, to compare cement plants with these technologies to those without, i.e., conventional cement production without any specific CO_2-eq. emissions mitigation measure implemented. The results are plotted in Fig. 2 (main text). Full details of the calculations are presented here, in Sections S7.1 and S7.2.

S7.1. Scenarios and assumptions

In our economic analysis we first estimated the costs of both conventional and CO_2 mineralisation products (€/t product), then, based on the results of the LCA study (detailed in Section S6; CO_2-eq. emissions avoided per technology substitution), we derived corresponding costs to avoid CO_2-eq. emissions (€/t CO_2-eq. avoided). The assumptions used in the economic analysis are shown in Table S12 and discussed below.

Table S12 | Cost assumptions for key energy and material inputs used in the economic analysis.

Raw materials	€/t product	Source/notes:
Fine limestone	3	[134]
GGBF/fly ash (Class F)	30	[135]
Fly ashes	30	[136]
Calcined clay	10.5	[137]
Sand and gravel	10	[138]
Gypsum	20	[137]
Recycled aggregates	14	[139]
Ammonium chloride	150	[140]
Natural gas	300	Value is in € per MWh, based on the average European market price in 2021
Electricity	40	Value is in € per MWh, based on the average European market price in 2021

Key assumptions that we adopted in the economic analysis include:

1. A minimal upfront investment cost for CO_2 injection into ready-mix concrete, based on a public declaration from industrial vendors[115]. As a result, we model a 2% increase in the cost of ready-mix concrete when the CO_2 injection process is adopted, as this only accounts for the additional cost associated with CO_2 separation.
2. For all carbonated products, we adopted the economic assumptions proposed in Pedraza et al. (2021)[141] to compute costs of the carbonation processes. That study estimated an investment cost for a carbonator reactor of €120M and operating costs of €29/t of carbonated product. The resulting carbonation cost is €41.7/t of product based on the energy prices reported in Table S12.

3. For cement from CCP, a maximum supply radius between the point of generation of end-of-life concrete to the cement plant of 50 km has been assumed, at a unit transport cost of €2/t, based on the economic analysis presented in Strunge et al. (2022)[140]. The final cost of cement from CCP is €55.7/t.
4. The final cost of precipitated calcium carbonate (from carbonatable solid materials) is €42/t. This cost accounts for the use of ammonium chloride (0.032 kg per kg of product) to extract calcium from carbonatable solid material feedstocks and for the additional energy required for feedstock/product drying (Fig. S4).
5. We consider ground granulated blast furnace slag as reference carbonatable solid material feedstocks for carbonated normal weight and carbonate bonded compacts, while for lightweight aggregates we adopted fly ashes (Class F).
6. The cost of carbonated concrete blocks (€28.8/t) assumes the concrete mix design presented in Table S8. The cost of CO_2 curing for concrete blocks is based on the cost increase for CO_2-cured precast concrete reported by Sick et al. (2022)[142].

In addition, for conventional products, the following assumptions were made:

1. The cost of composite PC is based on the cost figures proposed by De Lena et al. (2019)[134]. The study considers a greenfield cement kiln producing 2,800 t clinker per day and reports a capital cost of €203.7M following the main techno-economic assumptions proposed in the framework of the CEMCAP project[143]. These values result in a Portland clinker production cost of €60.8/t and corresponding cost of composite PC of €51.8/t, based on the mix designs presented in Table S8 and Section S5 and on the raw material costs presented in Table S12.
2. Based on these assumptions and considering 10% and 15% contents of clinker and cement in concrete, respectively (Table S8), we obtain a cost for the production of unreinforced concrete blocks of €21.3/t.
3. Costs of recycled aggregates, gravel, and expanded clay represent average values of publicly available market prices (Table S12).

Finally, we compared the costs of avoiding CO_2 emissions with CO_2 mineralisation products to the costs to produce cement using kilns retrofitted with carbon capture and storage technologies. We considered two archetypal carbon capture and storage technologies for this analysis: amine scrubbing and tail-end calcium looping. The final cost of producing composite PC (containing 75% Portland clinker) with carbon capture and storage are €93.3/t cement for amine scrubbing and €92.5/t cement for tail-end calcium looping. Detailed cost analyses and the main process parameters for these technologies can be found in IEAGHG (2021)[144].

Monte Carlo simulations were conducted to analyse the impacts of key uncertainties on the costs to produce conventional and CO_2 mineralisation products. To this end, 1,000 independent simulations were conducted for fuel and material inputs, as well as for the capital costs of carbonation and CCS processes. For each simulation, we used a normal distribution with a standard deviation of 20% with respect to the values reported in Table S11 and the capital costs of CCS and carbonation facilities [145,146]. The results of this statistical analysis were used to compute 1,000 observations of production cost increase and to derive the corresponding uncertainties, expressed as percentage errors (Table S13).

The results of this economic analysis are shown in Table S13.

Table S13 | Costs of conventional and comparable CO_2 mineralisation products. CCS is carbon capture and storage.

**Conventional product**	**CO_2 mineralisation substituting product**	**Increase in production cost** (€/t)	**Cost to avoid 1 t CO_2-eq.** (€/t CO_2-eq.)	**Uncertainty (±%)**
Portland cement (CEM I) concrete block, unreinforced	Carbonatable calcium silicate cement concrete block, unreinforced	7.47	161.6	5
Ready-mix composite Portland cement concrete [80% clinker]	CO_2 injected ready-mix composite Portland cement concrete [80% clinker]	0.82	151.5	16.4
Portland cement (CEM I) concrete block, unreinforced	Carbonated Portland cement concrete block, unreinforced	7.47	184.7	5
Composite Portland cement [75% clinker]	Cement from carbonated end-of-life cement paste [57% clinker]	4.4	22.4	45.3
Composite Portland cement [75% clinker]	Composite Portland cement with 75% clinker from carbonatable solid materials	3.5	37.1	32.2
Fine limestone	Precipitated calcium carbonate from carbonatable solid materials	39	139	3.8
Recycled aggregate	Carbonated recycled concrete aggregate	43.2	1323	5.6
Gravel	Carbonated normal weight aggregate from carbonatable solid materials	61.5	619.7	7.6
Expanded clay	Carbonated lightweight aggregate from carbonatable solid materials	53.5	108	8.7
Composite Portland cement [75% clinker]	Composite Portland cement with amine scrubbing (CCS)	42.0	101.3	11.9
Composite Portland cement [75% clinker]	Composite Portland cement with calcium looping (CCS)	41.3	73.9	10.4
Portland cement (CEM I) concrete block, unreinforced	Carbonate bonded compacts	50.1	221.2	7.8
Composite Portland cement [75% clinker]	Composite Portland cement with precipitated calcium carbonate from carbonatable solid materials [75% clinker]	3.8	54.8	39.5

S7.2. Economic analysis of cement from carbonated end-of-life cement paste

Due to the high product (Fig. 1, main text) and market (Fig. 3, main text) level decarbonisation potentials of cement from CCP and its high current economic competitiveness (Fig. 2, main text), we performed a sensitivity analysis on its economic performance in ca. 2020 to determine its practical potential more reliably. To serve as a reference in this analysis, we use the lower and upper ranges for the European emissions trading scheme (ETS) from early 2021 to 2022 (Fig. S14), as well as its lower (€32/t CO_2), mean (€60/t CO_2), and upper (€97/t CO_2) values over this period.

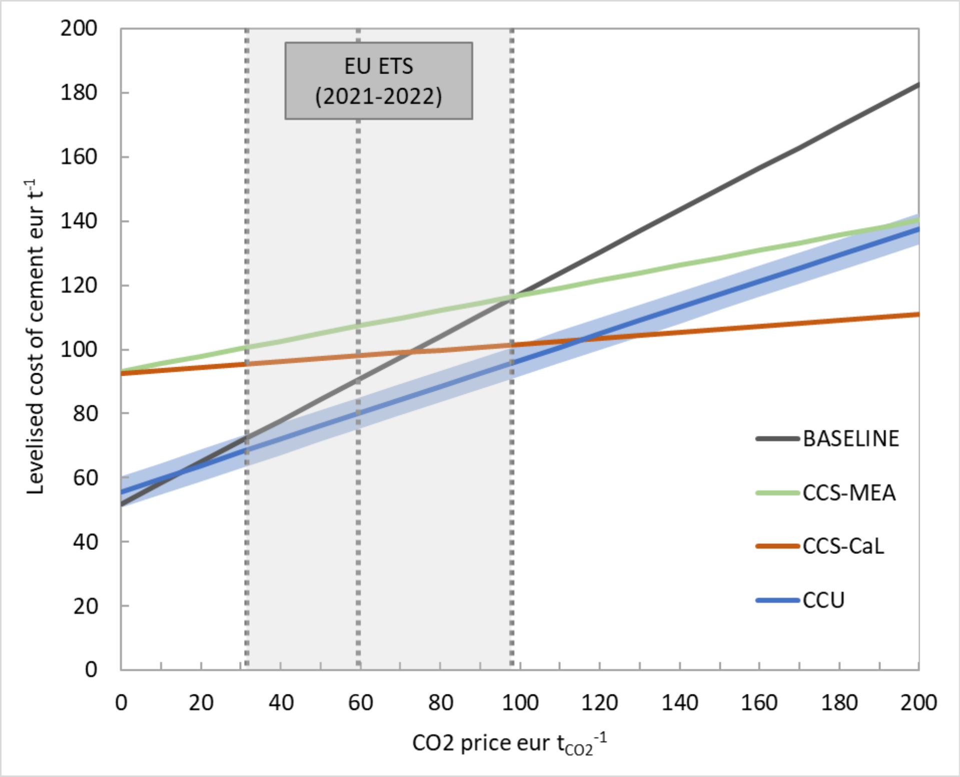

Fig. S14 | Comparison of the economic performance of conventional cement production ('BASELINE'), and cement from CCP ('CCU') and carbon capture and storage ('CCS-MEA', 'CCS-CaL') options for CO_2 mitigation from cement plants. This sensitivity analysis varies CO_2 price, showing its effect on the levelised cost of cement. The shaded region indicates the range of CO_2 prices for the EU emissions trading scheme (ETS) in the period from early 2021 to 2022.

An initial sensitivity analysis showed that cement from CCP is sensitive to (i) the cost to produce CCP, and (ii) the transport distance for end-of-life concrete, with reference values of €41.7/tonne [141] and 50 km, respectively. The blue region indicated in Fig. S14 shows upper and lower bounds for cement from CCP, assuming that end-of-life concrete is available on site (best case) or within a radius of 100 km (worst case), while the cost of CCP variates between ±20%. The results show that in the absence of a CO_2 price, the cost to produce cement from CCP in ca. 2020 was similar to conventional cement production (BASELINE), while retrofitting the cement plant with CCS results in an increase in cost to produce cement of €40.7/t and €41.5/t for tail-end calcium looping (CCS-CaL) and amine scrubbing (CCS-MEA), respectively.

Increasing the CO_2 price sees cement from CCP becoming cheaper, and carbon capture and storage decreasingly expensive, compared to conventional cement production in ca. 2020 (Fig. S14). Above a price of €25/tonne of CO_2, cement from CCP becomes the cheapest option, which continues until roughly €110/tonne of CO_2, after which the competitiveness of CCS-CaL begins to diminish the case for cement from CCP. By around €120/tonne CCS-CaL becomes the preferred option, while cement with CCS-MEA becomes cheaper than cement from CCP only with a sufficiently high carbon price , i.e. above €190/tonne, and for long end-of-life concrete

transport distances. These findings show that cement from CCP performs favourably compared to CCS at lower (or in the absence of) CO_2 prices. Hence, whilst the mitigation potential of cement from CCP is significantly lower than CCS, the higher capital cost of CCS processes compared to the cost of carbonation, makes cement from CCP the most competitive alternative to conventional cement production under current carbon market regimes.

S8. References in this Supplementary Information file

1. IEA. *Greenhouse gas emissions from energy data explorer*, <https://www.iea.org/reports/greenhouse-gas-emissions-from-energy-overview> (Accessed: 13 January 2023).

2. Naims, H. Economics of carbon dioxide capture and utilization—A supply and demand perspective. *Environmental Science and Pollution Research* **23**, 22226-22241 (2016). https://doi.org/10.1007/s11356-016-6810-2

3. IEA. *Industry*, <https://www.iea.org/reports/industry> (Accessed: 13 January 2023).

4. IEA. *Technology roadmap - Low-carbon transition in the cement industry*, <https://www.iea.org/reports/technology-roadmap-low-carbon-transition-in-the-cement-industry> (Accessed: 13 January 2023).

5. IEA. *Iron and steel technology roadmap*, <https://www.iea.org/reports/iron-and-steel-technology-roadmap> (Accessed: 13 January 2023).

6. Joint Research Centre, Institute for Prospective Technological Studies, Kourti, I., Delgado Sancho, L., Schorcht, F., Roudier, S. & Scalet, B. *Best available techniques (BAT) reference document for the production of cement, lime and magnesium oxide: Industrial emissions directive 2010/75/EU (integrated pollution prevention and control)*. (European Commission, 2013).

7. U.S. Environmental Protection Agency. Report to congress on cement kiln dust. (1993).

8. Hanein, T., Hayashi, Y., Utton, C., Nyberg, M., Martinez, J.-C., Quintero-Mora, N.-I. & Kinoshita, H. Pyro processing cement kiln bypass dust: Enhancing clinker phase formation. *Construction and Building Materials* **259**, 120420 (2020). https://doi.org/10.1016/j.conbuildmat.2020.120420

9. Kaliyavaradhan, S. K., Ling, T.-C. & Mo, K. H. CO_2 sequestration of fresh concrete slurry waste: Optimization of CO_2 uptake and feasible use as a potential cement binder. *Journal of CO_2 Utilization* **42**, 101330 (2020). https://doi.org/10.1016/j.jcou.2020.101330

10. Xuan, D., Zhan, B., Poon, C. S. & Zheng, W. Innovative reuse of concrete slurry waste from ready-mixed concrete plants in construction products. *Journal of Hazardous Materials* **312**, 65-72 (2016). https://doi.org/10.1016/j.jhazmat.2016.03.036

11. Paolini, M. & Khurana, R. Admixtures for recycling of waste concrete. *Cement and Concrete Composites* **20**, 221-229 (1998). https://doi.org/10.1016/S0958-9465(97)00066-8

12. Xuan, D., Zhan, B., Poon, C. S. & Zheng, W. Carbon dioxide sequestration of concrete slurry waste and its valorisation in construction products. *Construction and Building Materials* **113**, 664-672 (2016). https://doi.org/10.1016/j.conbuildmat.2016.03.109

13. Islam, R., Nazifa, T. H., Yuniarto, A., Shanawaz Uddin, A. S. M., Salmiati, S. & Shahid, S. An empirical study of construction and demolition waste generation and implication of recycling. *Waste Management* **95**, 10-21 (2019). https://doi.org/10.1016/j.wasman.2019.05.049

14. Akhtar, A. & Sarmah, A. K. Construction and demolition waste generation and properties of recycled aggregate concrete: A global perspective. *Journal of Cleaner Production* **186**, 262-281 (2018). https://doi.org/10.1016/j.jclepro.2018.03.085

15. Skocek, J., Zajac, M. & Ben Haha, M. Carbon capture and utilization by mineralization of cement pastes derived from recycled concrete. *Scientific Reports* **10**, 5614 (2020). https://doi.org/10.1038/s41598-020-62503-z

16. Shah, I. H., Miller, S. A., Jiang, D. & Myers, R. J. Cement substitution with secondary materials can reduce annual global CO_2 emissions by up to 1.3 gigatons. *Nature Communications* **13**, 5758 (2022). https://doi.org/10.1038/s41467-022-33289-7

17. Scrivener, K., John, V. M. & Gartner, E. M. Eco-efficient cements. (UNEP, 2016).

18. European Commission. *Cdw: Material recovery & backfilling*, <https://ec.europa.eu/environment/pdf/waste/studies/CDW%20Statistics%202011.pdf> (Accessed: 14 January 2023).

19. Zhang, C., Hu, M., van der Meide, M., Di Maio, F., Yang, X., Gao, X., Li, K., Zhao, H. & Li, C. Life cycle assessment of material footprint in recycling: A case of concrete recycling. *Waste Management* **155**, 311-319 (2023). https://doi.org/10.1016/j.wasman.2022.10.035

20. Zhang, C., Hu, M., Dong, L., Gebremariam, A., Miranda-Xicotencatl, B., Di Maio, F. & Tukker, A. Eco-efficiency assessment of technological innovations in high-grade concrete recycling. *Resources, Conservation and Recycling* **149**, 649-663 (2019). https://doi.org/10.1016/j.resconrec.2019.06.023

21. IEA. World energy outlook. (2019).

22. Yao, Z. T., Ji, X. S., Sarker, P. K., Tang, J. H., Ge, L. Q., Xia, M. S. & Xi, Y. Q. A comprehensive review on the applications of coal fly ash. *Earth-Science Reviews* **141**, 105-121 (2015). http://dx.doi.org/10.1016/j.earscirev.2014.11.016

23. Chindaprasirt, P., Jaturapitakkul, C., Chalee, W. & Rattanasak, U. Comparative study on the characteristics of fly ash and bottom ash geopolymers. *Waste Management* **29**, 539-543 (2009). https://doi.org/10.1016/j.wasman.2008.06.023

24. IEA. Energy technology perspectives. (2020).

25. World Steel Association. Steel industry co-products. (2010).

26. Quina, M. J., Bordado, J. C. & Quinta-Ferreira, R. M. Treatment and use of air pollution control residues from msw incineration: An overview. *Waste Management* **28**, 2097-2121 (2008). https://doi.org/10.1016/j.wasman.2007.08.030

27. Kaza, S., Yao, L. C., Bhada-Tata, P. & Van Woerden, F. What a waste 2.0: A global snapshot of solid waste management to 2050. (World Bank, 2018).

28. Massarutto, A. Economic aspects of thermal treatment of solid waste in a sustainable wm system. *Waste Management* **37**, 45-57 (2015). https://doi.org/10.1016/j.wasman.2014.08.024

29. Statista. *Paper consumption worldwide from 2021 to 2032*, <https://www.statista.com/statistics/1089078/demand-paper-globally-until-2030/> (Accessed: 1 May 2024).

30. Bajpai, P. Generation of waste in pulp and paper mills. In *Management of pulp and paper mill waste* (Ed. Bajpai, P.) 9-17 (Springer International Publishing, 2015).

31. Wang, S., Wen, Y., Hammarström, H., Jönsson, P. G. & Yang, W. Pyrolysis behaviour, kinetics and thermodynamic data of hydrothermal carbonization–treated pulp and paper mill sludge. *Renewable Energy* **177**, 1282-1292 (2021). https://doi.org/10.1016/j.renene.2021.06.027

32. Yoh, S., Sitepu, T. & Ambarita, H. Proximate, ultimate and calorific value analyses of paper industry sludge at different moisture content. *IOP Conference Series: Materials Science and Engineering* **851**, 012052 (2020). https://doi.org/10.1088/1757-899X/851/1/012052

33. Layr, K. & Hartlieb, P. Market analysis for urban mining of phosphogypsum. *BHM Berg- und Hüttenmännische Monatshefte* **164**, 245-249 (2019). https://doi.org/10.1007/s00501-019-0855-8

34. IFA. *Ifastat*, <https://www.ifastat.org/> (Accessed: 14 January 2023).

35. Cordell, D. & White, S. Life's bottleneck: Sustaining the world's phosphorus for a food secure future. *Annual Review of Environment and Resources* **39**, 161-188 (2014). https://doi.org/10.1146/annurev-environ-010213-113300

36. Statista. *Key figures on glass recycling worldwide as of 2018*, <https://www.statista.com/statistics/1055604/key-figures-glass-recycling-globally/> (Accessed: 14 January 2023).

37. UK Government: Department of Energy and Climate Change and the Department for Business Innovation and Skills. Industrial decarbonisation & energy efficiency roadmaps to 2050: Glass.

38. International Aluminium Institute. *Global aluminium cycle 2020*, <https://alucycle.world-aluminium.org/public/global/index.html> (Accessed: 14 January 2023).

39. Guo, Y., Li, J., Yan, K., Cao, L. & Cheng, F. A prospective process for alumina extraction via the co-treatment of coal fly ash and bauxite red mud: Investigation of the process. *Hydrometallurgy* **186**, 98-104 (2019). https://doi.org/10.1016/j.hydromet.2019.04.011

40. Balomenos, E., Panias, D. & Paspaliaris, I. Energy and exergy analysis of the primary aluminum production processes: A review on current and future sustainability. *Mineral Processing and Extractive Metallurgy Review* **32**, 69-89 (2011). https://doi.org/10.1080/08827508.2010.530721

41. Statista. *Projected water consumption from 2014 to 2040, by sector*, <https://www.statista.com/statistics/1012228/global-consumption-for-water-by-sector/> (Accessed: 14 January 2023).

42. Mininni, G., Laera, G., Bertanza, G., Canato, M. & Sbrilli, A. Mass and energy balances of sludge processing in reference and upgraded wastewater treatment plants. *Environmental Science and Pollution Research* **22**, 7203-7215 (2015). https://doi.org/10.1007/s11356-014-4013-2

43. Wang, Z., Chen, D., Song, X. & Zhao, L. Study on the combined sewage sludge pyrolysis and gasification process: Mass and energy balance. *Environmental Technology* **33**, 2481-2488 (2012). https://doi.org/10.1080/09593330.2012.683816

44. Almahbashi, N. M. Y., Kutty, S. R. M., Ayoub, M., Noor, A., Salihi, I. U., Al-Nini, A., Jagaba, A. H., Aldhawi, B. N. S. & Ghaleb, A. A. S. Optimization of preparation conditions of sewage sludge based activated carbon. *Ain Shams Engineering Journal* **12**, 1175-1182 (2021). https://doi.org/10.1016/j.asej.2020.07.026

45. Liang, S., Yang, L., Chen, H., Yu, W., Tao, S., Yuan, S., Xiao, K., Hu, J., Hou, H., Liu, B. & Yang, J. Phosphorus recovery from incinerated sewage sludge ash (ISSA) and reutilization of residues for sludge pretreated by different conditioners. *Resources, Conservation and Recycling* **169**, 105524 (2021). https://doi.org/10.1016/j.resconrec.2021.105524

46. International Standards Organisation. BS EN 197-1:2011: Cement: Part 1: Composition, specifications and conformity criteria for common cements. (British Standards Institution, London, 2011).

47. Machner, A., Zajac, M., Ben Haha, M., Kjellsen, K. O., Geiker, M. R. & De Weerdt, K. Portland metakaolin cement containing dolomite or limestone – similarities and differences in phase assemblage and compressive strength. *Construction and Building Materials* **157**, 214-225 (2017). https://doi.org/10.1016/j.conbuildmat.2017.09.056

48. Machner, A., Zajac, M., Ben Haha, M., Kjellsen, K. O., Geiker, M. R. & De Weerdt, K. Limitations of the hydrotalcite formation in portland composite cement pastes containing dolomite and metakaolin. *Cement and Concrete Research* **105**, 1-17 (2018). https://doi.org/10.1016/j.cemconres.2017.11.007

49. Scrivener, K., Martirena, F., Bishnoi, S. & Maity, S. Calcined clay limestone cements (LC3). *Cement and Concrete Research* **114**, 49-56 (2018). https://doi.org/10.1016/j.cemconres.2017.08.017

50. Hansen, B. S., Howard, I. L., Shannon, J., Cost, T. & Wilson, W. M. Portland-limestone cement fineness effects on concrete properties. *ACI Materials Journal* **117**, 157+ (2020). https://doi.org/10.14359/51720301

51. Sun, H., Hohl, B., Cao, Y., Handwerker, C., Rushing, T. S., Cummins, T. K. & Weiss, J. Jet mill grinding of portland cement, limestone, and fly ash: Impact on particle size, hydration rate, and strength. *Cement and Concrete Composites* **44**, 41-49 (2013). https://doi.org/10.1016/j.cemconcomp.2013.03.023

52. Bonavetti, V. L., Rahhal, V. F. & Irassar, E. F. Studies on the carboaluminate formation in limestone filler-blended cements. *Cement and Concrete Research* **31**, 853-859 (2001). https://doi.org/10.1016/S0008-8846(01)00491-4

53. Karen, S., François, A., Hamed, M., Franco, Z., Julien, S., Wilasinee, H. & Aurélie, F. Impacting factors and properties of limestone calcined clay cements (LC3). *Green Materials* **7**, 3-14 (2019). https://doi.org/10.1680/jgrma.18.00029

54. Lothenbach, B., Le Saout, G., Gallucci, E. & Scrivener, K. Influence of limestone on the hydration of portland cements. *Cement and Concrete Research* **38**, 848-860 (2008). http://dx.doi.org/10.1016/j.cemconres.2008.01.002

55. Stark, J., Freyburg, E. & Löhmer, K. Investigations into the influence of limestone additions to portland cement clinker phases on the early phase of hydration. In *Modern concrete materials: Binders, additions and admixtures*. 69-77.

56. Ramezanianpour, A. M. & Hooton, R. D. A study on hydration, compressive strength, and porosity of Portland-limestone cement mixes containing SCMs. *Cement and Concrete Composites* **51**, 1-13 (2014). https://doi.org/10.1016/j.cemconcomp.2014.03.006

57. Matschei, T., Lothenbach, B. & Glasser, F. P. The role of calcium carbonate in cement hydration. *Cement and Concrete Research* **37**, 551-558 (2007). https://doi.org/10.1016/j.cemconres.2006.10.013

58. Zajac, M., Rossberg, A., Le Saout, G. & Lothenbach, B. Influence of limestone and anhydrite on the hydration of portland cements. *Cement and Concrete Composites* **46**, 99-108 (2014). https://doi.org/10.1016/j.cemconcomp.2013.11.007

59. International Standards Organisation. ISO 19595:2017 natural aggregates for concrete. (2017).

60. Ren, P., Ling, T.-C. & Mo, K. H. Recent advances in artificial aggregate production. *Journal of Cleaner Production* **291**, 125215 (2021). https://doi.org/10.1016/j.jclepro.2020.125215

61. Hansen, T. C. *Recycling of demolished concrete and masonry*. 1st Edn. (CRC Press, 1992).

62. Marinković, S., Radonjanin, V., Malešev, M. & Ignjatović, I. Comparative environmental assessment of natural and recycled aggregate concrete. *Waste Management* **30**, 2255-2264 (2010). https://doi.org/10.1016/j.wasman.2010.04.012

63. Jimoh, O. A., Otitoju, T. A., Hussin, H., Ariffin, K. S. & Baharun, N. Understanding the precipitated calcium carbonate (PCC) production mechanism and its characteristics in the liquid-gas system using milk of lime (MOL) suspension. *South African Journal of Chemistry* **70**, 01-07 (2017). http://dx.doi.org/10.17159/0379-4350/2017/v70a1

64. Jimoh, O. A., Ariffin, K. S., Hussin, H. B. & Temitope, A. E. Synthesis of precipitated calcium carbonate: A review. *Carbonates and Evaporites* **33**, 331-346 (2018). https://doi.org/10.1007/s13146-017-0341-x

65. Zevenhoven, R., Legendre, D., Said, A. & Järvinen, M. Carbon dioxide dissolution and ammonia losses in bubble columns for precipitated calcium carbonate (PCC) production. *Energy* **175**, 1121-1129 (2019). https://doi.org/10.1016/j.energy.2019.03.112

66. Mun, M., Cho, H. & Kwon, J. Study on characteristics of various extractants for mineral carbonation of industrial wastes. *Journal of Environmental Chemical Engineering* **5**, 3803-3821 (2017). https://doi.org/10.1016/j.jece.2017.05.048

67. Hargis, C. W., Chen, I. A., Devenney, M., Fernandez, M. J., Gilliam, R. J. & Thatcher, R. P. Calcium carbonate cement: A carbon capture, utilization, and storage (CCUS) technique. *Materials* **14** (2021). https://doi.org/10.3390/ma14112709

68. Lee, M.-G., Kang, D., Yoo, Y., Jo, H., Song, H.-J. & Park, J. Continuous and simultaneous CO_2 absorption, calcium extraction, and production of calcium carbonate using ammonium nitrate. *Industrial & Engineering Chemistry Research* **55**, 11795-11800 (2016). https://doi.org/10.1021/acs.iecr.6b02880

69. Czaplicka, N. & Konopacka-Łyskawa, D. Utilization of gaseous carbon dioxide and industrial Ca-rich waste for calcium carbonate precipitation: A review. *Energies* **13**, 6239 (2020). https://doi.org/10.3390/en13236239

70. Sun, Y., Yao, M.-S., Zhang, J.-P. & Yang, G. Indirect CO_2 mineral sequestration by steelmaking slag with NH_4Cl as leaching solution. *Chemical Engineering Journal* **173**, 437-445 (2011). https://doi.org/10.1016/j.cej.2011.08.002

71. Eloneva, S., Teir, S., Salminen, J., Fogelholm, C.-J. & Zevenhoven, R. Steel converter slag as a raw material for precipitation of pure calcium carbonate. *Industrial & Engineering Chemistry Research* **47**, 7104-7111 (2008). https://doi.org/10.1021/ie8004034

72. Mattila, H.-P., Grigaliūnaitė, I. & Zevenhoven, R. Chemical kinetics modeling and process parameter sensitivity for precipitated calcium carbonate production from steelmaking slags. *Chemical Engineering Journal* **192**, 77-89 (2012). https://doi.org/10.1016/j.cej.2012.03.068

73. Jo, H., Young Jo, H. & Jang, Y.-N. Effect of extraction solutions on carbonation of cementitious materials in aqueous solutions. *Environmental Technology* **33**, 1391-1401 (2012). https://doi.org/10.1080/09593330.2011.630422

74. Iizuka, A., Sasaki, T., Honma, M., Yoshida, H., Hayakawa, Y., Yanagisawa, Y. & Yamasaki, A. Pilot-scale operation of a concrete sludge recycling plant and simultaneous production of calcium carbonate. *Chemical Engineering Communications* **204**, 79-85 (2017). https://doi.org/10.1080/00986445.2016.1235564

75. Iizuka, A., Sakai, Y., Yamasaki, A., Honma, M., Hayakawa, Y. & Yanagisawa, Y. Bench-scale operation of a concrete sludge recycling plant. *Industrial & Engineering Chemistry Research* **51**, 6099-6104 (2012). https://doi.org/10.1021/ie300620u

76. Katsuyama, Y., Yamasaki, A., Iizuka, A., Fujii, M., Kumagai, K. & Yanagisawa, Y. Development of a process for producing high-purity calcium carbonate ($CaCO_3$) from waste cement using pressurized CO_2. *Environmental Progress* **24**, 162-170 (2005). https://doi.org/10.1002/ep.10080

77. Zajac, M., Skocek, J., Durdzinski, P., Bullerjahn, F., Skibsted, J. & Ben Haha, M. Effect of carbonated cement paste on composite cement hydration and performance. *Cement and Concrete Research* **134**, 106090 (2020). https://doi.org/10.1016/j.cemconres.2020.106090

78. Zajac, M., Skibsted, J., Durdzinski, P., Bullerjahn, F., Skocek, J. & Ben Haha, M. Kinetics of enforced carbonation of cement paste. *Cement and Concrete Research* **131**, 106013 (2020). https://doi.org/10.1016/j.cemconres.2020.106013

79. Zajac, M., Lechevallier, A., Durdzinski, P., Bullerjahn, F., Skibsted, J. & Ben Haha, M. Co_2 mineralisation of portland cement: Towards understanding the mechanisms of enforced carbonation. *Journal of CO_2 Utilization* **38**, 398-415 (2020). https://doi.org/10.1016/j.jcou.2020.02.015

80. Zajac, M., Skocek, J., Skibsted, J. & Ben Haha, M. Co_2 mineralization of demolished concrete wastes into a supplementary cementitious material – A new CCU approach for the cement industry. *RILEM Technical Letters* **6**, 53-60 (2021). https://doi.org/10.21809/rilemtechlett.2021.141

81. Zajac, M., Skibsted, J. & Ben Haha, M. Effect of alkalis on enforced carbonation of cement paste: Mechanism of reaction. *Journal of the American Ceramic Society* **104**, 1076-1087 (2021). https://doi.org/10.1111/jace.17481

82. Zajac, M., Skibsted, J., Durdzinski, P. & Ben Haha, M. Effect of alkalis on products of enforced carbonation of cement paste. *Construction and Building Materials* **291**, 123203 (2021). https://doi.org/10.1016/j.conbuildmat.2021.123203

83. Torrenti, J. M., Amiri, O., Barnes-Davin, L., Bougrain, F., Braymand, S., Cazacliu, B., Colin, J., Cudeville, A., Dangla, P., Djerbi, A., Doutreleau, M., Feraille, A., Gueguen, M., Guillot, X., Hou, Y., Izoret, L., Jacob, Y.-P., Jeong, J., Hiu Hoong, J. D. L., Mahieux, P.-Y., Mai-Nhu, J., Martinez, H., Meyer, V., Morin, V., Pernin, T., Potier, J.-M., Poulizac, L.,

Rougeau, P., Saadé, M., Schmitt, L., Sedran, T., Sereng, M., Soive, A., Dos Reys, G. S. & Turcry, P. The Fastcarb project: Taking advantage of the accelerated carbonation of recycled concrete aggregates. *Case Studies in Construction Materials* **17**, e01349 (2022). https://doi.org/10.1016/j.cscm.2022.e01349

84. Izoret, L., Pernin, T., Potier, J.-M. & Torrenti, J.-M. Impact of industrial application of fast carbonation of recycled concrete aggregates. *Applied Sciences* **13**, 849 (2023).

85. Pu, Y., Li, L., Wang, Q., Shi, X., Fu, L., Zhang, G., Luan, C. & Abomohra, A. E.-F. Accelerated carbonation treatment of recycled concrete aggregates using flue gas: A comparative study towards performance improvement. *Journal of CO_2 Utilization* **43**, 101362 (2021). https://doi.org/10.1016/j.jcou.2020.101362

86. Zajac, M., Skocek, J., Gołek, Ł. & Deja, J. Supplementary cementitious materials based on recycled concrete paste. *Journal of Cleaner Production* **387**, 135743 (2023). https://doi.org/10.1016/j.jclepro.2022.135743

87. Tiefenthaler, J., Braune, L., Bauer, C., Sacchi, R. & Mazzotti, M. Technological demonstration and life cycle assessment of a negative emission value chain in the Swiss concrete sector. *Frontiers in Climate* **3** (2021). https://doi.org/10.3389/fclim.2021.729259

88. Liang, C., Pan, B., Ma, Z., He, Z. & Duan, Z. Utilization of CO_2 curing to enhance the properties of recycled aggregate and prepared concrete: A review. *Cement and Concrete Composites* **105**, 103446 (2020). https://doi.org/10.1016/j.cemconcomp.2019.103446

89. Leemann, A. CO_2 adsorption of concrete based on the boundary conditions of switzeralnd, in *International Workshop on the CO_2 Storage in Concrete – CO2STO2019.* (RILEM) (2019).

90. Pade, C. & Guimaraes, M. The CO_2 uptake of concrete in a 100 year perspective. *Cement and Concrete Research* **37**, 1348-1356 (2007). https://doi.org/10.1016/j.cemconres.2007.06.009

91. Bravo, M., de Brito, J., Pontes, J. & Evangelista, L. Mechanical performance of concrete made with aggregates from construction and demolition waste recycling plants. *Journal of Cleaner Production* **99**, 59-74 (2015). https://doi.org/10.1016/j.jclepro.2015.03.012

92. Xiao, J., Li, W., Fan, Y. & Huang, X. An overview of study on recycled aggregate concrete in China (1996–2011). *Construction and Building Materials* **31**, 364-383 (2012). https://doi.org/10.1016/j.conbuildmat.2011.12.074

93. Shi, M., Ling, T.-C., Gan, B. & Guo, M.-Z. Turning concrete waste powder into carbonated artificial aggregates. *Construction and Building Materials* **199**, 178-184 (2019). https://doi.org/10.1016/j.conbuildmat.2018.12.021

94. Xuan, D., Zhan, B. & Poon, C. S. Durability of recycled aggregate concrete prepared with carbonated recycled concrete aggregates. *Cement and Concrete Composites* **84**, 214-221 (2017). https://doi.org/10.1016/j.cemconcomp.2017.09.015

95. Singh, M., Danie Roy, A. B., Waseem, S. & Singh, H. Feasibility and performance analysis of carbonated recycled aggregate concrete. *International Journal of Sustainable Engineering* **14**, 761-775 (2021). https://doi.org/10.1080/19397038.2020.1856967

96. Ajdukiewicz, A. & Kliszczewicz, A. Influence of recycled aggregates on mechanical properties of hs/hpc. *Cement and Concrete Composites* **24**, 269-279 (2002). https://doi.org/10.1016/S0958-9465(01)00012-9

97. Poon, C. S., Shui, Z. H., Lam, L., Fok, H. & Kou, S. C. Influence of moisture states of natural and recycled aggregates on the slump and compressive strength of concrete. *Cement and Concrete Research* **34**, 31-36 (2004). https://doi.org/10.1016/S0008-8846(03)00186-8

98. Batayneh, M., Marie, I. & Asi, I. Use of selected waste materials in concrete mixes. *Waste Management* **27**, 1870-1876 (2007). https://doi.org/10.1016/j.wasman.2006.07.026

99. Xuan, D., Zhan, B. & Poon, C. S. Assessment of mechanical properties of concrete incorporating carbonated recycled concrete aggregates. *Cement and Concrete Composites* **65**, 67-74 (2016). https://doi.org/10.1016/j.cemconcomp.2015.10.018

100. Zhang, J., Shi, C., Li, Y., Pan, X., Poon, C.-S. & Xie, Z. Performance enhancement of recycled concrete aggregates through carbonation. *Journal of Materials in Civil Engineering* **27**, 04015029 (2015). https://doi.org/10.1061/(ASCE)MT.1943-5533.0001296

101. Li, X. Recycling and reuse of waste concrete in China: Part I. Material behaviour of recycled aggregate concrete. *Resources, Conservation and Recycling* **53**, 36-44 (2008). https://doi.org/10.1016/j.resconrec.2008.09.006

102. Domingo-Cabo, A., Lázaro, C., López-Gayarre, F., Serrano-López, M. A., Serna, P. & Castaño-Tabares, J. O. Creep and shrinkage of recycled aggregate concrete. *Construction and Building Materials* **23**, 2545-2553 (2009). https://doi.org/10.1016/j.conbuildmat.2009.02.018

103. Levy, S. M. & Helene, P. Durability of recycled aggregates concrete: A safe way to sustainable development. *Cement and Concrete Research* **34**, 1975-1980 (2004). https://doi.org/10.1016/j.cemconres.2004.02.009

104. Otsuki, N., Miyazato, S.-i. & Yodsudjai, W. Influence of recycled aggregate on interfacial transition zone, strength, chloride penetration and carbonation of concrete. *Journal of Materials in Civil Engineering* **15**, 443-451 (2003). https://doi.org/10.1061/(ASCE)0899-1561(2003)15:5(443)

105. Ann, K. Y., Moon, H. Y., Kim, Y. B. & Ryou, J. Durability of recycled aggregate concrete using pozzolanic materials. *Waste Management* **28**, 993-999 (2008). https://doi.org/10.1016/j.wasman.2007.03.003

106. Thomas, J., Thaickavil, N. N. & Wilson, P. M. Strength and durability of concrete containing recycled concrete aggregates. *Journal of Building Engineering* **19**, 349-365 (2018). https://doi.org/10.1016/j.jobe.2018.05.007

107. Gunning, P. J., Hills, C. D. & Carey, P. J. Production of lightweight aggregate from industrial waste and carbon dioxide. *Waste Management* **29**, 2722-2728 (2009). https://doi.org/10.1016/j.wasman.2009.05.021

108. Nielsen, P., Baciocchi, R., Costa, G., Quaghebeur, M. & Snellings, R. Carbonate-bonded construction materials from alkaline residues. *RILEM Technical Letters* **2**, 53-58 (2017). https://doi.org/10.21809/rilemtechlett.2017.50

109. Quaghebeur, M., Nielsen, P., Horckmans, L. & Van Mechelen, D. Accelerated carbonation of steel slag compacts: Development of high-strength construction materials. *Frontiers in Energy Research* **3** (2015). https://doi.org/10.3389/fenrg.2015.00052

110. Di Maria, A., Snellings, R., Alaerts, L., Quaghebeur, M. & Van Acker, K. Environmental assessment of CO_2 mineralisation for sustainable construction materials. *International Journal of Greenhouse Gas Control* **93**, 102882 (2020). https://doi.org/10.1016/j.ijggc.2019.102882

111. Baciocchi, R., Costa, G., Di Bartolomeo, E., Polettini, A. & Pomi, R. Carbonation of stainless steel slag as a process for CO_2 storage and slag valorization. *Waste and Biomass Valorization* **1**, 467-477 (2010). https://doi.org/10.1007/s12649-010-9047-1

112. Jiang, Y., Ling, T.-C. & Shi, M. Strength enhancement of artificial aggregate prepared with waste concrete powder and its impact on concrete properties. *Journal of Cleaner Production* **257**, 120515 (2020). https://doi.org/10.1016/j.jclepro.2020.120515

113. Srivastava, S., Snellings, R. & Cool, P. Clinker-free carbonate-bonded (CFCB) products prepared by accelerated carbonation of steel furnace slags: A parametric overview of the process development. *Construction and Building Materials* **303**, 124556 (2021). https://doi.org/10.1016/j.conbuildmat.2021.124556

114. Librandi, P., Nielsen, P., Costa, G., Snellings, R., Quaghebeur, M. & Baciocchi, R. Mechanical and environmental properties of carbonated steel slag compacts as a function of mineralogy and CO_2 uptake. *Journal of CO_2 Utilization* **33**, 201-214 (2019). https://doi.org/10.1016/j.jcou.2019.05.028

115. CarbonCure. <https://www.carboncure.com/> (Accessed: 8 July 2021).

116. Monkman, S., MacDonald, M., Hooton, R. D. & Sandberg, P. Properties and durability of concrete produced using CO_2 as an accelerating admixture. *Cement and Concrete Composites* **74**, 218-224 (2016). https://doi.org/10.1016/j.cemconcomp.2016.10.007

117. Monkman, S. & MacDonald, M. On carbon dioxide utilization as a means to improve the sustainability of ready-mixed concrete. *Journal of Cleaner Production* **167**, 365-375 (2017). https://doi.org/10.1016/j.jclepro.2017.08.194

118. Zhang, D., Ellis, B. R., Jaworska, B., Hu, W.-H. & Li, V. C. Carbonation curing for precast engineered cementitious composites. *Construction and Building Materials* **313**, 125502 (2021). https://doi.org/10.1016/j.conbuildmat.2021.125502

119. Hilal El-Hassan, Y. S. & Zaid, G. Effect of initial curing on carbonation of lightweight concrete masonry units. *ACI Materials Journal* **110** https://doi.org/10.14359/51685791

120. Young, J. F., Berger, R. L. & Breese, J. Accelerated curing of compacted calcium silicate mortars on exposure to CO_2. *Journal of the American Ceramic Society* **57**, 394-397 (1974). https://doi.org/10.1111/j.1151-2916.1974.tb11420.x

121. Meyer, V., Sahu, S. & Dunster, A. Properties of Solidia cement and concrete, in *1st International Conference on Innovation in Low-Carbon Cement & Concrete Technology*. (2019).

122. Miller, S. A. & Myers, R. J. Environmental impacts of alternative cement binders. *Environmental Science & Technology* **54**, 677-686 (2020). https://doi.org/10.1021/acs.est.9b05550

123. Atakan, V., Sahu, S., Quinn, S., Hu, X. & DeCristofaro, N. Why CO_2 matters – Advances in a new class of cement. *ZKG International* **3** (2014).

124. Gartner, E. & Sui, T. Alternative cement clinkers. *Cement and Concrete Research* **114**, 27-39 (2018). https://doi.org/10.1016/j.cemconres.2017.02.002

125. Solidia. <https://www.solidiatech.com/> (Accessed: 31 December 2021).

126. Farnam, Y., Villani, C., Washington, T., Spence, M., Jain, J. & Jason Weiss, W. Performance of carbonated calcium silicate based cement pastes and mortars exposed to NaCl and $MgCl_2$ deicing salt. *Construction and Building Materials* **111**, 63-71 (2016). https://doi.org/10.1016/j.conbuildmat.2016.02.098

127. Dekeukelaere, A. Evaluation of a new low CO_2 cement introduction within the European Emission Trading System. (2020).

128. Cembureau. Activity report. (2020).

129. Favier, A., De Wolf, C., Scrivener, K. & Habert, G. *A sustainable future for the European cement and concrete industry: Technology assessment for full decarbonisation of the industry by 2050* (2018).

130. Cembureau. Cements for a low-carbon Europe. (2012).

131. Miller, S. A., John, V. M., Pacca, S. A. & Horvath, A. Carbon dioxide reduction potential in the global cement industry by 2050. *Cement and Concrete Research* **114**, 115-124 (2018). https://doi.org/10.1016/j.cemconres.2017.08.026

132. Weidema, B. P., Bauer, C., Hischier, R., Mutel, C., Nemecek, T., Reinhard, J., Vadenbo, C. O. & Wernet, G. Overview and methodology. Data quality guideline for the ecoinvent database version 3. Ecoinvent report no.1 (v3). (The ecoinvent Centre, St. Gallen, Switerland, 2013).

133. Hertwich, E. G. Increased carbon footprint of materials production driven by rise in investments. *Nature Geoscience* **14**, 151-155 (2021). https://doi.org/10.1038/s41561-021-00690-8

134. De Lena, E., Spinelli, M., Gatti, M., Scaccabarozzi, R., Campanari, S., Consonni, S., Cinti, G. & Romano, M. C. Techno-economic analysis of calcium looping processes for low CO_2 emission cement plants. *International Journal of Greenhouse Gas Control* **82**, 244-260 (2019). https://doi.org/10.1016/j.ijggc.2019.01.005

135. Department for Business, Energy & Industrial Strategy. Fly ash and blast furnace slag for cement manufacturing: BEIS research paper No. 19. (2017).

136. Robl, T. L. & McCormick, C. J. We are running out of fly ash: The nature of regional supply problems. (2015).

137. Scrivener, K., Dekeukelaere, A., Avet, F. & Grimmeissen, L. Financial attractiveness of LC3. (2019).

138. Statista. *Average price of construction sand and gravel in the U.S. from 2010 to 2021*, <https://www.statista.com/statistics/219381/sand-and-gravel-prices-in-the-us/> (Accessed:

139. Ohemeng, E. A. & Ekolu, S. O. Comparative analysis on costs and benefits of producing natural and recycled concrete aggregates: A South African case study. *Case Studies in Construction Materials* **13**, e00450 (2020). https://doi.org/10.1016/j.cscm.2020.e00450

140. Strunge, T., Renforth, P. & Van der Spek, M. Towards a business case for CO_2 mineralisation in the cement industry. *Communications Earth & Environment* **3**, 59 (2022). https://doi.org/10.1038/s43247-022-00390-0

141. Pedraza, J., Zimmermann, A., Tobon, J., Schomäcker, R. & Rojas, N. On the road to net zero-emission cement: Integrated assessment of mineral carbonation of cement kiln dust. *Chemical Engineering Journal* **408**, 127346 (2021). https://doi.org/10.1016/j.cej.2020.127346

142. Sick, V., Stokes, G. & Mason, F. C. CO_2 utilization and market size projection for CO_2-treated construction materials. *Frontiers in Climate* **4** (2022). https://doi.org/10.3389/fclim.2022.878756

143. De Lena, E., Spinelli, M., Romano, M. C., Voldsund, M., Gardarsdottir, S. O. & Roussanaly, S. Cemcap economic model spreadsheet: Annex of cemcap deliverable d4.6. (2018).

144. IEAGHG. Assessing the techno-economic performance, opportunities and challenges of mature and nearly-mature negative emissions technologies (NETS). (2021).

145. Freire Ordóñez, D., Halfdanarson, T., Ganzer, C., Shah, N., Dowell, N. M. & Guillén-Gosálbez, G. Evaluation of the potential use of e-fuels in the European aviation sector: A comprehensive economic and environmental assessment including externalities. *Sustainable Energy & Fuels* **6**, 4749-4764 (2022). https://doi.org/10.1039/D2SE00757F

146. Sinnott, R. & Towler, G. *Chemical engineering design*. 6th Edn. (2020).
